# DEAD-box helicase 17 (DDX17) protects cardiac function by promoting mitochondrial homeostasis in heart failure

**DOI:** 10.1038/s41392-024-01831-2

**Published:** 2024-05-24

**Authors:** Mingjing Yan, Junpeng Gao, Ming Lan, Que Wang, Yuan Cao, Yuxuan Zheng, Yao Yang, Wenlin Li, Xiaoxue Yu, Xiuqing Huang, Lin Dou, Bing Liu, Junmeng Liu, Hongqiang Cheng, Kunfu Ouyang, Kun Xu, Shenghui Sun, Jin Liu, Weiqing Tang, Xiyue Zhang, Yong Man, Liang Sun, Jianping Cai, Qing He, Fuchou Tang, Jian Li, Tao Shen

**Affiliations:** 1 The Key Laboratory of Geriatrics, Beijing Institute of Geriatrics, Institute of Geriatric Medicine, Chinese Academy of Medical Sciences, Beijing Hospital/National Center of Gerontology of National Health Commission, Beijing, 100730 China; 2 Peking University Fifth School of Clinical Medicine, Beijing, 100730 China; 3 Department of Laboratory Medicine, Huashan Hospital, Shanghai Medical College, Fudan University, Shanghai, 200040 China; 4 Biomedical Pioneering Innovation Center, School of Life Sciences, Peking University, Beijing, 100871 China; 5 Emergency Center, Zhongnan Hospital of Wuhan University, Wuhan, 430071 China; 6 Department of Cardiology, Beijing Hospital, National Center of Gerontology, Institute of Geriatric Medicine, Chinese Academy of Medical Sciences & Peking Union Medical College, Beijing, 100730 China; 7 Graduate School of Peking Union Medical College, Beijing, 100730 China; 8 Department of Health Care, Beijing Hospital, National Center of Gerontology, Institute of Geriatric Medicine, Chinese Academy of Medical Sciences, Beijing, 100730 China; 9 Peking-Tsinghua Center for Life Sciences, Academy for Advanced Interdisciplinary Studies, Peking University, Beijing, 100871 China; 10 Department of Pathology and Pathophysiology and Department of Cardiology at Sir Run Run Shaw Hospital, Zhejiang University School of Medicine, Hangzhou, 310058 China; 11 Department of Cardiovascular Surgery, Peking University Shenzhen Hospital, Shenzhen, 518036 China; 12 Experimental Technology Center for Life Sciences at Beijing Normal University, Beijing, 100875 China

**Keywords:** Molecular biology, Cardiology

## Abstract

DEAD-box helicase 17 (DDX17) is a typical member of the DEAD-box family with transcriptional cofactor activity. Although DDX17 is abundantly expressed in the myocardium, its role in heart is not fully understood. We generated cardiomyocyte-specific *Ddx17-*knockout mice (*Ddx17*-cKO), cardiomyocyte-specific *Ddx17* transgenic mice (*Ddx17*-Tg), and various models of cardiomyocyte injury and heart failure (HF). DDX17 is downregulated in the myocardium of mouse models of heart failure and cardiomyocyte injury. Cardiomyocyte-specific knockout of *Ddx17* promotes autophagic flux blockage and cardiomyocyte apoptosis, leading to progressive cardiac dysfunction, maladaptive remodeling and progression to heart failure. Restoration of DDX17 expression in cardiomyocytes protects cardiac function under pathological conditions. Further studies showed that DDX17 can bind to the transcriptional repressor B-cell lymphoma 6 (BCL6) and inhibit the expression of dynamin-related protein 1 (DRP1). When DDX17 expression is reduced, transcriptional repression of BCL6 is attenuated, leading to increased DRP1 expression and mitochondrial fission, which in turn leads to impaired mitochondrial homeostasis and heart failure. We also investigated the correlation of DDX17 expression with cardiac function and DRP1 expression in myocardial biopsy samples from patients with heart failure. These findings suggest that DDX17 protects cardiac function by promoting mitochondrial homeostasis through the BCL6-DRP1 pathway in heart failure.

## Introduction

Heart failure is a decline in cardiac function caused by a variety of conditions that reduce myocardial contractility and can lead to cardiovascular events with poor prognosis.^1^ With the development of an aging society, heart failure has become a major cause of hospitalization in patients over the age of 65, thus causing serious health and economic problems.^1,2^ Coronary atherosclerosis, hypertension, diabetes, cardiomyopathy, and chemotherapeutic drugs all contribute to the development of cardiac dysfunction and heart failure. Therefore, it is important to discover and elucidate new mechanisms of heart failure development. Emerging evidence suggests that transcriptional regulation is involved in the pathogenesis of heart failure. Transcriptional regulation includes the synergistic action of transcription factors and cofactors, which regulate the expression of various structural genes, energy metabolism genes, calcium-regulated genes and cell survival and death genes in cardiomyocytes and are involved in the maintenance of normal cardiac function, or lead to the development of heart failure in pathological conditions.

DDX17 is a typical member of the DEAD box family with the ability to regulate RNA metabolism. Recent studies have shown that as a transcriptional cofactor, DDX17 involves in modulating the activity of transcription factors and the transcriptional regulation of their downstream gene expression. Research on DDX17 has mainly focused on oncology studies, including tumorigenesis and tumor progression in colon cancer and breast cancer.^3–5^ DDX17 is abundantly expressed in cardiomyocytes and has been shown to be potentially involved in the development of cardiac hypertrophy and doxorubicin-induced cardiomyocyte injury in vitro.^6,7^ However, the function of DDX17 in cardiomyocytes and its role in the development of heart failure remain unclear.

Transcriptional repressor BCL6 was originally thought to regulate the differentiation and immune response of B lymphocytes.^8–10^ Recent studies have shown that BCL6 also plays an important role in many diseases.^11–13^ BCL6 acts on multiple targets, including signal transducers, transcriptional activators and microRNAs, to promote the development of many types of cancer.^12^ BCL6 binds to specific DNA sequences in the promoter regions of target genes and exerts transcriptional repression that regulates cell proliferation, cell differentiation, DNA damage repair, maintenance of genome stability and cell cycle.^9^ Inhibition of BCL6 can lead to cell cycle arrest, gene instability and apoptosis.^14^ In the cardiovascular system, BCL6 can inhibit doxorubicin-induced cardiomyocyte senescence by activating PPARδ.^13^ *Bcl6*-knockout mice develop severe Th2-type inflammatory diseases, including myocarditis and pulmonary vasculitis, which suggesting that BCL6 negatively regulates the inflammatory response in myocardium.^15^ Knockdown of *Bcl6* under hypoxia can significantly increase the levels of the pro-inflammatory cytokines TNF-α, IL-1 and IL-6, which promote myocardial inflammatory responses.^16^ However, the regulatory mechanism of BCL6 in cardiomyocytes remains unclear.

The failing heart has been described as an “engine without fuel”. Mitochondria are the main source of energy for cardiomyocytes, and dysregulated mitochondrial homeostasis and impaired myocardial bioenergetic metabolism are considered to be key factors in the onset and progression of heart failure. DRP1-dependent mitochondrial fission plays an important role in mitochondrial homeostasis, as evidenced by the initial recruitment and assembly of DRP1 at mitochondrial fission foci, and increased DRP1 activity promotes mitochondrial fission.^17^ Excessive mitochondrial fission leads to reduced mitochondrial mass and dysfunction, resulting in structural and functional impairment of cardiomyocytes.^18^ In fact, many studies have confirmed the increased expression of DRP1 in ischemia-reperfusion injury and heart failure caused by excessive mitochondrial fission and cardiomyocyte injury.^19^ Therefore, DRP1 is one of the key regulators of cardiomyocyte injury and heart failure by controlling the balance of mitochondrial fission and fusion.

Here, we found that DDX17 is one of the pivotal regulators that maintain mitochondrial homeostasis and cardiac function, and its decreased expression is closely correlated with cardiac dysfunction. Our results also revealed the mechanism of the DDX17-BCL6-DRP1 signaling pathway in heart failure, which could be used as a potential therapeutic target for the mitochondrial dysregulation-induced cardiovascular diseases, including myocardial ischemia-reperfusion injury, myocardial infarction, and heart failure.

## Results

### DDX17 expression is reduced in models of heart failure and cardiomyocyte injury

**Fig. 1:**
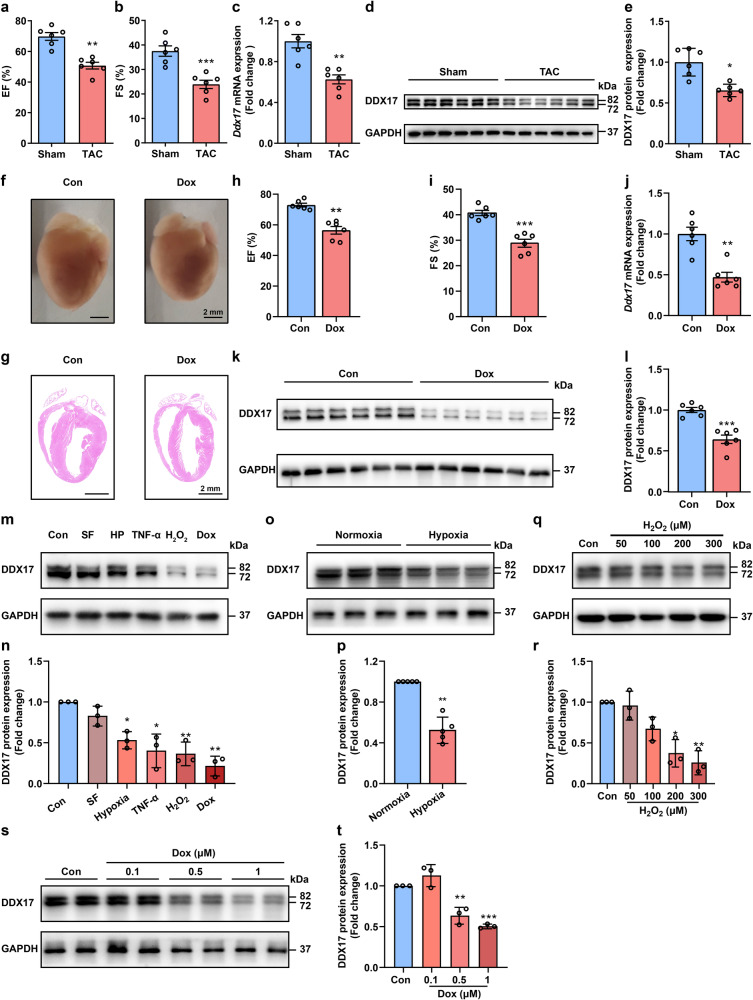
Heart failure and cardiomyocyte injury caused by various pathological factors lead to decreased DDX17 expression. **a**, **b** The average data of mouse cardiac function as shown by the left ventricular EF and FS of the sham and TAC-induced chronic heart failure mice (*n* = 6). **c**–**e** *Ddx17* mRNA and protein expression in the myocardium from the sham or TAC-induced chronic heart failure mouse models (*n* = 6). **f** Representative images of hearts showing the cardiac morphology of the control (Con) and Dox-treated (Dox) mice (*n* = 6); scale bar, 2 mm. **g** Representative images of H&E-stained heart sections from the Con and Dox-treated mice (*n* = 6); scale bar, 2 mm. **h**, **i** Mouse cardiac function as shown by the left ventricular EF and FS of Con and Dox-treated mice (*n* = 6). **j**–**l** *Ddx17* mRNA (*n* = 6) and protein (*n* = 6) expression in Con and Dox-treated mouse hearts. **m**, **n** DDX17 expression in NMVMs treated with different injury factors: serum-free medium (SF), hypoxia for 16 h (HP), 2 μg/mL TNF-α, 100 μM H_2_O_2_, and 0.5 μM Dox (*n* = 3). **o**, **p** DDX17 protein expression in NMVMs treated with normoxia or hypoxia for 16 h (*n* = 5). **q**, **r** DDX17 protein levels and the average data of NMVMs treated with different concentrations of H_2_O_2_ for 24 h (*n* = 3). **s**, **t** DDX17 protein levels and the average data of NMVMs treated with 0.1, 0.5, and 1 μM Dox for 24 h (*n* = 3). *P < 0.05, **P < 0.01, and ***P < 0.001

To investigate the expression of DDX17 in the mouse heart failure model, we established a chronic heart failure mouse model with transverse aortic constriction (TAC) and an acute heart failure mouse model with doxorubicin (Dox). Twelve weeks after the TAC surgery, the left ventricular ejection fraction (EF) and fractional shortening (FS) of the mice were significantly reduced (Fig. 1a, b). Western blot showed that TAC-induced heart failure caused cardiomyocyte apoptosis (Supplementary Fig. 1a–c) and significantly reduced *Ddx17* mRNA and protein expression in the myocardium (Fig. 1c–e).

The acute heart failure mouse model was established by intraperitoneal injection of 7.5 mg/kg Dox three times every other day to 10-week-old male C57BL/6 J mice (Supplementary Fig. 1d). Six days after the first intraperitoneal injection of Dox, mice in the Dox group had significantly lower body weight (Supplementary Fig. 1e), decreased heart weight/tibial length ratio (Supplementary Fig. 1f), smaller heart volume and thinner ventricular walls compared to controls (Fig. 1f, g). Left ventricular EF and FS (Fig. 1h, i), left ventricular mass (LV mass) (Supplementary Fig. 1g) and left ventricular anterior wall thickness at end-systole (LVAW;s) (Supplementary Fig. 1h), as well as ventricular wall thickness and interventricular septum thickness analyzed by whole heart HE staining (Fig. 1g, Supplementary Fig. 1i, j) in Dox-treated mice were significantly reduced compared to those in control mice. In addition, H&E staining showed that Dox resulted in cytosolic vacuolation of cardiomyocytes (Supplementary Fig. 1k, l). Based on the western blot results, including increased LC3B-II/I and p62 expression, a significant blockade of autophagic flux was detected in the hearts of Dox-treated mice compared with that of the control mice (Supplementary Fig. 1m–o). Terminal deoxynucleotidyl transferase dUTP nick end labeling (TUNEL) staining also revealed an increased rate of cell apoptosis in the hearts of Dox-treated mice (Supplementary Fig. 1p, q). Consistent with the TUNEL staining results, the western blot data also revealed an increased cleaved caspase-3/caspase-3 (c-CASP-3/CASP-3) ratio and a decreased B-cell lymphoma 2/BCL2-associated X protein (BCL2/BAX) ratio in the myocardial tissue of Dox-treated mice (Supplementary Fig. 1r–t). Interestingly, DDX17 mRNA and protein downregulation was also observed in the hearts of Dox-treated mice (Fig. 1j–l). Therefore, the decreased expression of DDX17 may be closely correlated with heart failure caused by various pathological stimuli in vivo.

Next, we isolated and cultured neonatal mouse ventricular myocytes (NMVMs) and analyzed the expression of DDX17 in cardiomyocyte injury models with different stimuli: serum-free medium culture (SF), hypoxia for 16 h (HP), 2 μg/mL TNF-α, 100 μM H_2_O_2_, and 0.5 μM Dox. A variety of severe cardiomyocyte injuries, particularly those induced by Dox, hypoxia, H_2_O_2_ and inflammation, led to decreased expression of DDX17. Among them, Dox injury led to the most significant DDX17 reduction in cardiomyocytes (Fig. 1m, n). More importantly, we observed a similar decrease in DDX17 expression in hypoxia-treated cardiomyocytes (Fig. 1o, p) and H_2_O_2_-treated cardiomyocytes in a concentration-dependent manner by western blot (Fig. 1q, r). Furthermore, we also found a concentration-dependent decrease in DDX17 expression after Dox treatment (Fig. 1s, t), and this effect also led to a significant increase in cardiomyocyte autophagic flux blockage as evidenced by increased autophagic vesicles and increased LC3bII/I and p62 (Supplementary Fig. 2a–e). There was also a significant increase in cardiomyocyte apoptosis in Dox-treated NMVMs compared to the control by TUNEL staining (Supplementary Fig. 2f, g) and western blot (Supplementary Fig. 2h–j). Therefore, these data suggest that the decreased expression of DDX17 may be one of the common phenotypes of cardiomyocyte injury caused by various pathological stimuli.

From the results of in vivo and in vitro experiments, it was found that aberrant reduction of DDX17 expression may be one of the common pathways in the development of cardiomyocyte injury and heart failure by many pathological factors. Therefore, in the following experiments, we used a stable in vivo model of acute heart failure established by intraperitoneal injection of Dox into mice and cellular injury models such as doxorubicin, hypoxia, and H_2_O_2_ to explore the role and mechanism of DDX17 in the development of heart failure.

### Deletion of DDX17 in cardiomyocytes leads to cardiac dysfunction and promotes the progression of heart failure under pathological conditions

**Fig. 2:**
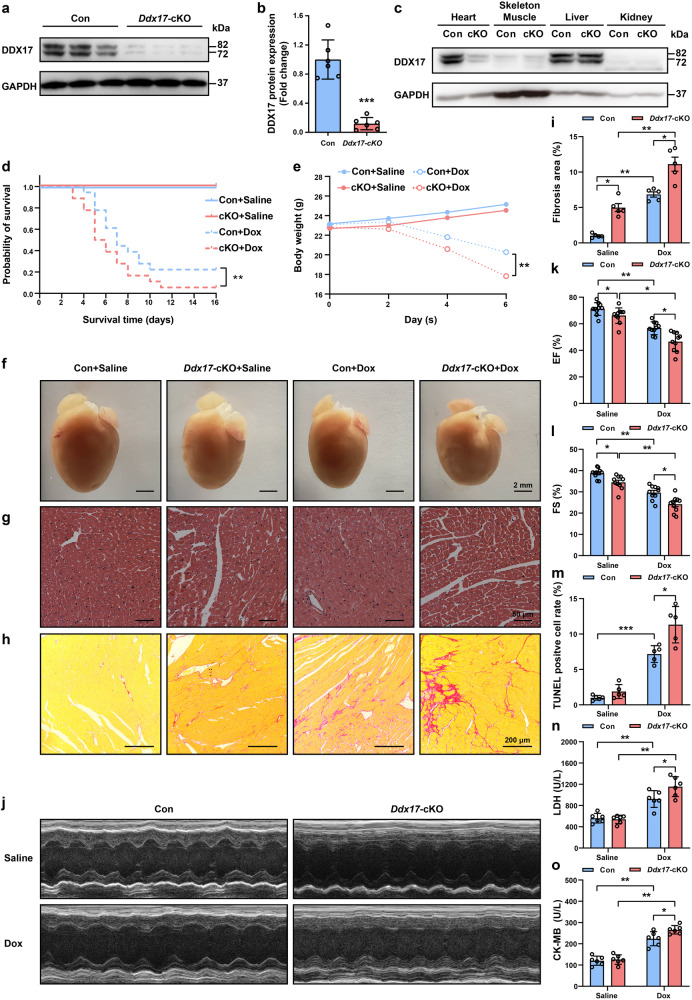
Cardiomyocyte-specific *Ddx17* knockout leads to reduced cardiac function and exacerbates Dox-induced heart failure in mice. **a**, **b** Western blot and the average data of DDX17 in isolated NMVMs of control (Con) and *Ddx17-*cKO mice (*n* = 6). **c** Western blot of DDX17 levels in heart, skeletal muscle, liver and kidney of Con and *Ddx17*-cKO mice (*n* = 3). **d** Mice were injected intraperitoneally with saline or 7.5 mg/kg Dox 3 times every other day. Survival curve of the mice in the four groups (*n* = 18). **e** Body weight of mice in the control + saline (Con + Saline), *Ddx17-*cKO + saline (*Ddx17-*cKO + Saline), control + doxorubicin (Con + Dox) and *Ddx17-*cKO + doxorubicin (*Ddx17-*cKO + Dox) groups (*n* = 5). **f** Representative images of the hearts from the mice in Con + Saline, *Ddx17-*cKO + Saline, Con + Dox, and *Ddx17-*cKO + Dox groups, the scale bar represents 2 mm. **g** Representative H&E-stained heart sections from mice in the four groups (*n* = 5); scale bar, 50 μm. **h**, **i** Representative images of Sirius Red-stained hearts from mice in the four groups and semiquantitative analysis of the fibrosis area ratio (*n* = 5); scale bar, 200 μm. **j**–**l** Representative images of echocardiography of mouse hearts and the average data of cardiac function of left ventricular EF (**k**) and FS (**l**) in the four groups; *n* = 10 for each group. **m** TUNEL staining quantification results of myocardial tissue in the four groups; *n* = 5 for each group. **n**, **o** Mouse serum LDH (**n**) and CK-MB (**o**) levels in the four groups (*n* = 6). *P < 0.05, **P < 0.01, and ***P < 0.001

To further investigate the function of DDX17 in cardiomyocytes, exon 3 of the *Ddx17* gene was targeted for deletion by insertion of two *loxP* sequences using CRISPR/Cas9 technology (Supplementary Fig. 3a, b). We then generated cardiomyocyte-specific *Ddx17*-knockout mice (*Ddx17*-cKO) by crossing *Ddx17*^*f/f*^ mice with mice expressing Cre recombinase under the control of the α-myosin heavy chain (*α-MHC*) promoter (Supplementary Fig. 3c). Immunofluorescence staining for DDX17 from control and *Ddx17*-cKO mice showed that the number of DDX17 positive cells was significantly lower in cardiac tissues of *Ddx17*-cKO mice (*Ddx1*7 was only knocked out in cardiomyocytes; other cell types still expressed DDX17 protein) (Supplementary Fig. 3d). Compared with the control group, the DDX17 expression was significantly decreased in the left ventricular myocardium of *Ddx17*-cKO mice. Because non-cardiac myocytes still express DDX17, some DDX17-positive cells were also present in myocardial tissue sections. Therefore, we isolated NMVMs (cardiomyocyte purity is ~85–90%) from the control and *Ddx17*-cKO mice, respectively, and found that DDX17 expression was further reduced in the *Ddx17*-cKO group, demonstrating the specificity and efficiency of *Ddx17* cardiomyocyte knockout (Fig. 2a, b). Furthermore, DDX17 expression was not altered in other organs or tissues, such as skeletal muscle, liver and kidney in *Ddx17*-cKO mice (Fig. 2c).

In *Ddx17*-cKO mice, the mortality rate, body weight, and cardiac morphology were not significantly different from those of control mice under basal conditions (Fig. 2d–f). H&E staining revealed the loss of myocardial orientation and disorganization of structures in *Ddx17*-cKO mice (Fig. 2g). Sirus red staining revealed a slight increase in the area of myocardial fibrosis in *Ddx17-*cKO mice compared to controls (Fig. 2h, i). And cardiac echocardiographic analysis showed that *Ddx17*-cKO mice had reduced left ventricular EF and FS (Fig. 2j–l). These results suggest that under basal conditions, cardiomyocyte-specific knockout of *Ddx17* did not significantly change the mortality rate, but there was a mild decrease in cardiac function and an increase in the area of cardiac tissue fibrosis compared with the control. This suggests that DDX17 plays an important regulatory role in maintaining normal cardiac structure and function.

The cardiomyocyte-specific *Ddx17* knockout exacerbated the Dox-induced increase in death rate and body weight loss compared with control mice (Fig. 2d, e). In addition, cardiac morphological abnormalities were more pronounced in the *Ddx17*-cKO mice after Dox-induced heart failure (Fig. 2f, g). Cardiac fibrosis levels (Fig. 2h, i) and cell apoptosis rates (Fig. 2m and Supplementary Fig. 3e) of the *Ddx17*-cKO mice were also significantly higher than those of the control mice after Dox treatment. Cardiomyocyte-specific *Ddx17* knockout further reduced Dox-induced left ventricular EF and FS (Fig. 2j–l), increased serum cardiac enzymes lactate dehydrogenase (LDH) and creatine kinase-MB (CK-MB) (Fig. 2n, o).

Embryonic and fibrosis genes were also altered (Supplementary Fig. 3f–i). Under basal conditions, *Ddx17*-cKO mice showed slightly increased expression of natriuretic peptide type A (*Nppa*) and collagen type III alpha 1 chain (*Col3a1*) mRNA compared to control mice. After Dox treatment, the expression of *Nppa*, natriuretic peptide type B (*Nppb*) and collagen type I alpha 1 chain (*Col1a1*) mRNA was also increased in *Ddx17*-cKO mice compared to control mice.

Thus, DDX17 plays a very important role in maintaining normal cardiac structure and function, and its cardiomyocyte-specific knockout can lead to abnormalities in cardiac structure and function and aggravate Dox-induced heart failure.

### Overexpression of *Ddx17* in cardiomyocytes reduces myocardial injury and improves cardiac function under pathological conditions

**Fig. 3:**
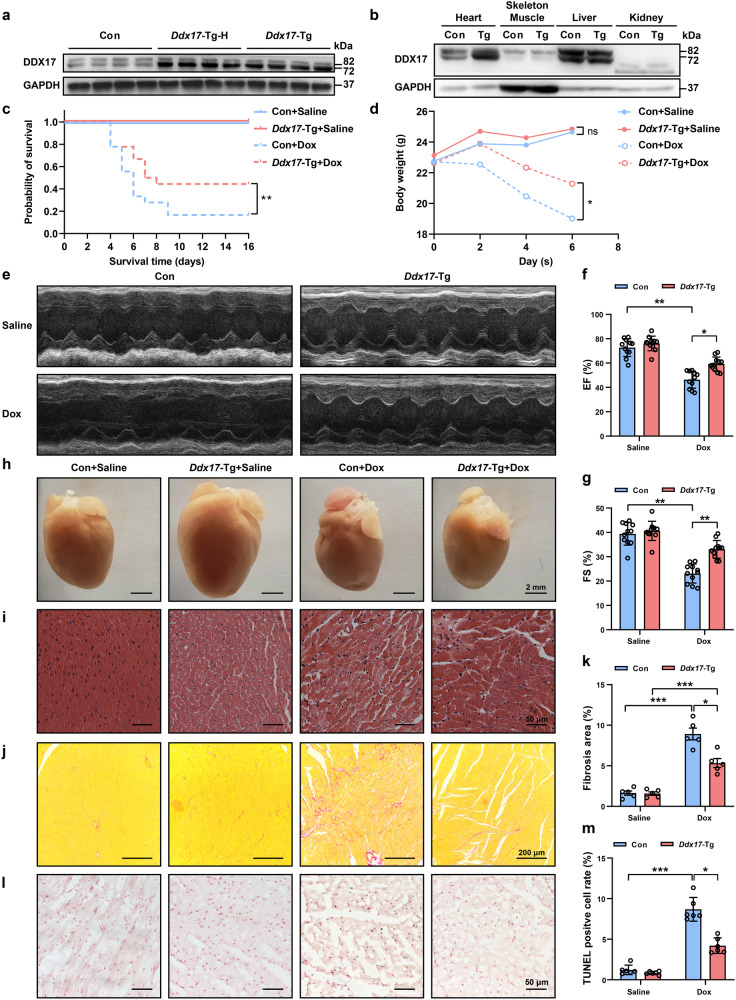
Overexpression of *Ddx17* in cardiomyocytes attenuates myocardial injury and improves cardiac function under pathological conditions. **a** Western blot of DDX17 protein expression from control (Con) and two cardiac-specific *Ddx17*-overexpressing mouse lines (*Ddx17*-Tg-H and *Ddx17*-Tg) (*n* = 5). **b** DDX17 expression in heart, skeletal muscle, liver and kidney of control (Con) and *Ddx17*-Tg mice (*n* = 3). **c** Doxorubicin (7.5 mg/kg) or an equivalent volume of saline was administered to mice by intraperitoneal injection every other day for a total of three injections in the control (Con) and *Ddx17*-Tg mice. Survival curves of mice in the control + saline (Con + Saline), *Ddx17*-transgene + saline (*Ddx17*-Tg + Saline), control + doxorubicin (Con + Dox), and *Ddx17*-transgene + doxorubicin (*Ddx17*-Tg + Dox) groups (*n* = 18). **d** Body weight of the mice in the Con + Saline, *Ddx17*-Tg + Saline, Con + Dox, and *Ddx17*-Tg + Dox groups (*n* = 6). **e**–**g** Representative images of echocardiography of mouse heart and the average data of cardiac function of left ventricle EF (**f**) and FS (**g**) in the four groups (*n* = 11). **h**, **i** Representative images of heart morphologies and H&E-stained heart sections in the four groups (*n* = 6). **j**–**k** Representative images of Sirius Red-stained mouse heart sections and quantification of myocardial fibrosis area ratio in the four groups (*n* = 5); scale bar, 200 μm. **l** TUNEL staining of myocardial tissue in Con + Saline, *Ddx17*-Tg + Saline, Con + Dox, and *Ddx17*-Tg + Dox, (*n* = 5); scale bar, 50 μm. **m** Quantification of TUNEL staining of myocardial tissue in the four groups (*n* = 5). *P < 0.05, **P < 0.01, and ***P < 0.001

To further analyze the function of DDX17 in vivo, *Ddx17*-Tg mice were generated by overexpressing *Ddx17* driven by the *α-MHC* promoter (Supplementary Fig. 4a, b). We developed two different *Ddx17*-transgenic mouse lines with different DDX17 protein levels: the *Ddx17*-Tg and *Ddx17*-Tg-H lines showed 2.53-fold and 3.38-fold increase, respectively, compared to the wild-type littermate control (Con) (Fig. 3a and Supplementary Fig. 4c). We then selected *Ddx17*-Tg mice with moderate DDX17 expression for follow-up studies. We also determined the specificity of *Ddx17* overexpression in cardiac myocytes and found that the expression of DDX17 in other tissues was not significantly different from that in the wild-type control group (Fig. 3b).

*Ddx17*-Tg mice had an increased survival rate and body weight compared to control mice after Dox treatment (Fig. 3c, d). In addition, the cardiomyocyte-specific overexpression of *Ddx17* significantly attenuated the decrease in cardiac function (Fig. 3e–g) and cardiac structure abnormality (Fig. 3h, i) after Dox treatment compared with that in the controls. Cardiomyocyte-specific overexpression of *Ddx17* decreased cardiac fibrosis (Fig. 3j, k) and cell apoptosis (Fig. 3l, m) after Dox treatment compared to that in controls. Thus, these data confirm that DDX17 overexpression in cardiomyocytes can attenuate Dox-induced myocardial structural abnormalities and cardiac dysfunction, which may inhibit the development of heart failure.

### DDX17 replenishment in cardiomyocytes attenuates autophagic flux blockage and apoptosis induced by pathological stimuli

Previously, our and other research teams have demonstrated that Dox treatment can cause cardiomyocyte autophagic flux blockage and apoptosis, leading to cardiac dysfunction and heart failure.^20^ Interestingly, cardiomyocyte-specific knockout of *Ddx17* also exacerbated Dox-induced autophagic flux blockage and apoptosis in myocardial tissue in vivo (Supplementary Fig. 5a–f). In contrast, the cardiomyocyte-specific overexpression of *Ddx17* significantly attenuated Dox-induced autophagic flux blockage and cell apoptosis compared to that in the control group in vivo (Supplementary Fig. 5g–l).

To determine whether replenishing DDX17 can reduce cardiomyocyte injury, we isolated and cultured primary NMVMs and transfected them with empty vector adenovirus (Con) or *Ddx17*-overexpressing adenovirus (*Ddx17*-OE). *Ddx17* overexpression also significantly inhibited H_2_O_2_-induced autophagic flux blockage and apoptosis, as indicated by the LC3B-II/I, p62 and c-CASP-3 expression levels (Supplementary Fig. 6a–f). In addition, we demonstrated the protective effects of DDX17 on cardiomyocytes using hypoxia-induced cardiomyocyte injury models (Supplementary Fig. 6g–j). Therefore, our results confirm that replenishment of DDX17 can inhibit cardiomyocyte injury caused by various pathological factors (Dox, H_2_O_2_ and hypoxia) by alleviating cell autophagic dysfunction and apoptosis.

### DDX17 is a key regulator of myocardial mitochondrial homeostasis

Mitochondrial oxidative phosphorylation is the major source of energy for the normal heart. Therefore, mitochondrial dysfunction or imbalance in mitochondrial homeostasis is one of the key mechanisms responsible for reduced myocardial contractile function, and is closely related to the onset and progression of heart failure. Mitochondrial quality control can protect cardiomyocytes from injury and preserve cardiac function. Deregulation of mitochondrial fusion, fission, and mitophagy can lead to loss of cardiomyocyte function or death, resulting in the onset and progression of heart failure.^17,21^

**Fig. 4:**
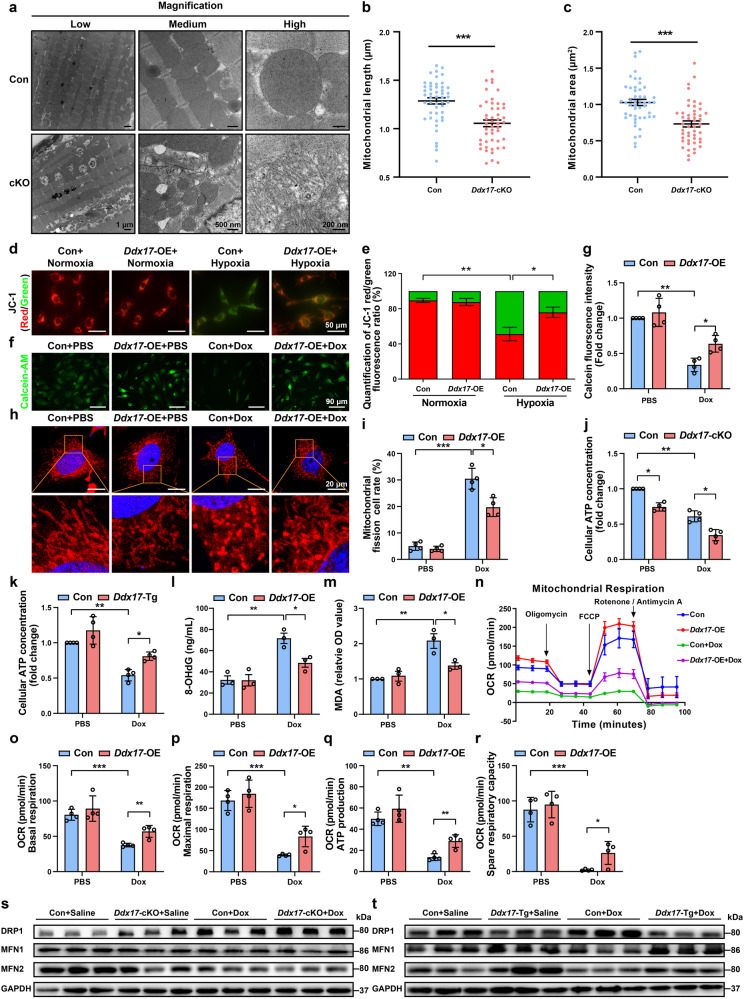
DDX17 plays an important role in maintaining mitochondrial morphology and function in cardiomyocytes. **a** Transmission electron microscopy (TEM) of LVs from control (Con) and *Ddx17*-cardiomyocyte-specific knockout (cKO) mice (scale bars: low-1 μm, medium-500 nm, high-200 nm) (*n* = 5). **b**, **c** Statistical analysis of mitochondrial length and mitochondrial area in control (Con) and *Ddx17*-cKO mice (*n* = 48). **d**, **e** Mitochondrial membrane potential (ΔΨm) analyzed by JC-1 red/green fluorescence intensity in control (Con) and *Ddx17*-overexpressing (*Ddx17*-OE) HL-1 cells treated with normoxic and hypoxic conditions (*n* = 3); scale bar: 50 μm. **f**, **g** Mitochondrial permeability transition pore (mPTP) analyzed by calcein-AM fluorescence intensity in control (Con) and *Ddx17*-overexpressing (*Ddx17*-OE) HL-1 cells (*n* = 4); scale bar: 90 μm. **h**, **i** NMVMs were infected with *Ddx17*-overexpressing adenovirus (*Ddx17*-OE) or its control (Con) for 24 h and then treated with PBS or Dox for 24 h. Mitochondria of cardiomyocytes were stained with Mito-Tracker Red and nuclei were stained with DAPI, and the rate of mitochondrial fission was analyzed by confocal microscopy (*n* = 4); scale bar corresponds to 20 μm. **j** Cellular ATP concentration in the NMVMs of control (Con) and *Ddx17*-cKO mice treated with PBS or Dox (*n* = 4)**. k** Cellular ATP concentration in the NMVMs of control (Con) and *Ddx17*-Tg mice treated with PBS or Dox (*n* = 4)**. l**, **m** 8-OHdG and MDA levels in NMVMs from each group (*n* = 3). **n**–**r** HL-1 cardiomyocytes were infected with *Ddx17* overexpressing adenovirus (*Ddx17*-OE) or its control (Con) for 24 h and then treated with PBS or 0.5 μM Dox for 24 h. Based on the measured mitochondrial OCR of HL-l cells in response to 1 μM oligomycin, 1 μM FCCP and 0.5 μM rotenone/antimycin A, the basal respiration, maximal respiration, ATP production and spare respiratory capacity were measured using a Seahorse flux analyser (*n* = 4). **s** Western blot of DRP1, MFN1 and MFN2 in the left ventricle of mice in the control + saline (Con + Saline), *Ddx17*-cKO + saline *(Ddx17*-cKO + Saline), control + doxorubicin (Con + Dox) and *Ddx17*-cKO + doxorubicin (*Ddx17*-cKO + Dox) groups (*n* = 6). **t** Western blot of DRP1, MFN1 and MFN2 in LVs from mice in the control + saline (Con + Saline), *Ddx17*-transgene + saline (*Ddx17*-Tg + Saline), control + doxorubicin (Con + Dox) and *Ddx17*-transgene+doxorubicin (*Ddx17*-Tg + Dox) groups (*n* = 6). *P < 0.05, **P < 0.01, and ***P < 0.001

*Ddx17* knockout results in reduced cardiac function and exacerbates cardiac dysfunction induced by various pathological stimuli. Transmission electron microscopy results showed significant structural abnormalities in the myocardial tissue of *Ddx17-*cKO mice, which mainly manifested as disorganized myocardial tissue structure, markedly increased myocardial fibrosis, disorganized mitochondrial arrangement, smaller mitochondria, and loose mitochondrial structure (Fig. 4a). Further analysis of mitochondrial morphology showed that *Ddx17*-cardiomyocyte knockout caused a significant reduction in mitochondrial length and mitochondrial area in cardiomyocytes compared to controls (Fig. 4b, c). To investigate the effect of DDX17 on mitochondrial function in cardiomyocytes, we overexpressed DDX17 in the HL-1 cell line. After exposure to hypoxia for 5 h, the mitochondrial membrane potential was detected by JC-1 staining. As shown in Fig. 4d, e, the mitochondrial membrane potential (reflected by JC-1 red/green fluorescence) was significantly higher in *Ddx17*-overexpressing cardiomyocytes than in control cardiomyocytes. We also used the fluorescent probe calcein acetoxymethyl ester (calcein AM) and CoCl_2_ to analyze the opening of the mitochondrial permeability transition pore (mPTP). After Dox treatment for 12 h, the mitochondrial membrane permeability and mPTP opening were significantly reduced in *Ddx17*-overexpressing (*Ddx17*-OE) cardiomyocytes compared to the control group (Fig. 4f, g). Overexpression of *Ddx17* attenuated Dox-induced abnormal mitochondrial distribution and morphology, and reduced the proportion of mitochondrial fissions in NMVMs (Fig. 4h, i). Interestingly, after Dox treatment, *Ddx17* knockout exacerbated the decrease in ATP concentration in NMVMs, whereas *Ddx17* overexpression significantly upregulated ATP concentration and protected cardiomyocyte function (Fig. 4j, k). Meanwhile, *Ddx17* overexpression inhibited Dox-induced increases in 8-hydroxy-2’-deoxyguanosine (8-OHdG) and malondialdehyde (MDA) in NMVMs (Fig. 4l, m), suggesting that the replenishment of *Ddx17* expression inhibited Dox-induced mitochondrial damage and oxidative stress in cardiomyocytes. Overexpression of *Ddx17* in HL-1 cardiomyocyte reversed Dox-induced decreases in basal respiration, maximal respiration, ATP production and respiratory reserve as analyzed by mitochondrial oxygen consumption rate (OCR) and improved mitochondrial function (Fig. 4n–r). Taken together, our results suggest that loss of DDX17 can lead to the deregulation of mitochondrial homeostasis and loss of cardiomyocyte function, whereas replenishment of DDX17 protects cardiomyocytes and inhibits the development of heart failure.

Mitochondrial fusion and fission are essential for maintaining of mitochondrial number, structure and function. An imbalance between fusion and fission leads to mitochondrial dysfunction, excessive oxidative stress and myocardial injury.^21^ As expected, the cardiomyocyte-specific knockout of *Ddx17* resulted in upregulation of the mitochondrial fission protein DRP1 compared to control mice. In contrast, *Ddx17* knockout decreased the expression of the mitochondrial fusion proteins mitofusin1 (MFN1) and mitofusin2 (MFN2) (Fig. 4s, Supplementary Fig. 7a–c). Notably, the cardiomyocyte-specific overexpression of *Ddx17* effectively inhibited DRP1 expression and reversed the Dox-induced upregulation of DRP1 expression and downregulation of mitochondrial fusion genes (Fig. 4t, Supplementary Fig. 7d–f). Consistent with the Dox-induced cardiomyocyte injury model, we observed similar DRP1, MFN1 and MFN2 expression in *Ddx17*-overexpressing NMVMs treated with hypoxia and 100 μM H_2_O_2_ (Supplementary Fig. 7g–n). In addition, cardiomyocyte *Ddx17*-cKO resulted in a significant increase in the mitochondrial fission 1 (FIS1), but there was no significant difference in the expression of the mitochondrial fission factor (MFF) and the mitochondrial fusion protein optic atrophy 1 (OPA1) (Supplementary Fig. 7o–r). Taken together, these data suggest that DDX17 plays an important role in maintaining mitochondrial homeostasis and function and is involved in the maintenance of normal cardiomyocyte function.

### DDX17 regulates mitochondrial fission and homeostasis by interacting with the transcription suppressor BCL6

Previous studies have found that DDX17 has a transcriptional coregulator function and is involved in regulating the activity of a variety of transcription factors.^3^ We analyzed the subcellular distribution of DDX17 in cardiomyocytes using immunofluorescence staining. The results showed that more than 95% of DDX17 was expressed in the nucleus of cardiomyocytes under basal conditions, while the amount of DDX17 in the cytoplasm of the cells was <5% (Supplementary Fig. 8a). This suggests that DDX17 may exert its major biological functions mainly in the cardiomyocyte nucleus.

**Fig. 5:**
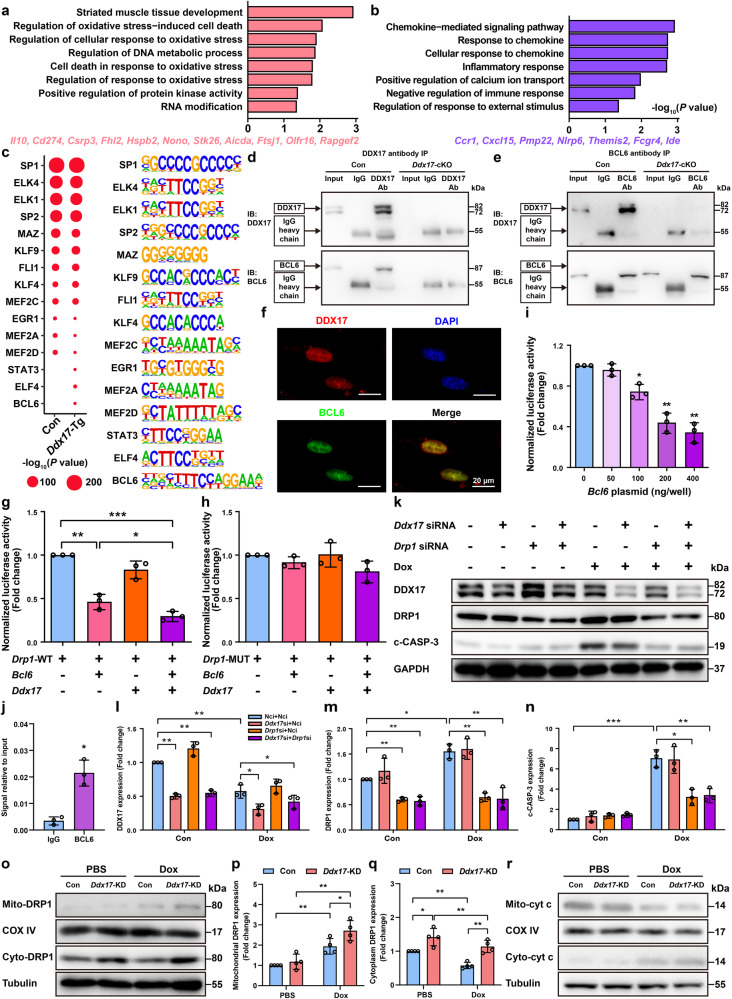
DDX17 coordinates with BCL6 in the transcriptional repression of the *Drp1* gene in cardiomyocytes. **a**, **b** GO terms responding to promoters with higher (**a**) and lower (**b**) methylation levels in NMVMs from *Ddx17*-Tg mice compared to controls (Con) (*n* = 3). Representative genes are indicated below. **c** Motif enrichment analysis of proximal NDRs in *Ddx17*-Tg and control (Con) NMVMs (*n* = 3). **d**, **e** Co-IP experiments were performed with DDX17 (**d**) and BCL6 (**e**) antibodies to analyze the interaction of DDX17 and BCL6 in control (Con) and *Ddx17*-cKO NMVMs (*n* = 4). **f** Immunofluorescence staining of DDX17 (red), BCL6 (green) and nuclei (DAPI, blue) in cultured wild-type NMVMs; scale bar, 20 μm (*n* = 3). **g** To investigate the regulatory effects of DDX17 and BCL6 overexpression on *Drp1* promoter activity, HEK293A cells were transfected with *Ddx17* (pcDNA-*Ddx17*) and/or *Bcl6* (pcDNA-*Bcl6*) expression plasmids and simultaneously cotransfected with the *Drp1* 0.8-kb wild-type promoter (pGL3-*Drp1*-WT) luciferase reporter plasmid, and *Drp1* promoter activity was analyzed by luciferase assay (*n* = 3). **h** HEK293A cells were transfected with *Ddx17* (pcDNA-*Ddx17*) and/or *Bcl6* (pcDNA-*Bcl6*) expression plasmids and simultaneously cotransfected with the 0.8 kb *Drp1* promoter mutation plasmid (pGL3-*Drp1*-MUT) with mutated BCL6 binding sites using the pGL3-basic plasmid (*n* = 3). **i** HEK293A cells were cotransfected with the *Drp1* promoter plasmid (pGL3-*Drp1*-WT) and different concentrations of the *Bcl6* expression plasmid (pcDNA-*Bcl6*) to detect *Drp1* promoter activity (*n* = 3). **j** ChIP analysis of NMVMs revealed the recruitment of BCL6 to regions containing BCL6 binding sites within the promoter region of *Drp1* by quantitative real-time PCR (*n* = 3). **k**–**n** HL-1 cells were transfected with *Ddx17* siRNA and/or *Drp1* siRNA for 24 h and then treated with doxorubicin for 24 h. Expression of DDX17, DRP1 and c-CASP-3 was analyzed by western blot, and GAPDH was used as a protein loading control (*n* = 3). In all co-transfection experiments, pcDNA3.1 was used as the equilibrium plasmid in the different transfection mixtures to balance the total amount of DNA, and NCi was used as the equilibrium RNA in the different transfection mixtures to balance the total amount of RNA (*n* = 3). **o**–**q** HL-1 cardiomyocytes were transfected with NCi (Con) or *Ddx17* siRNA (*Ddx17*-KD) for 24 h and then treated with PBS or 0.5 μM Dox for 24 h. Cardiomyocyte mitochondria and cytoplasm were isolated and the expression of mitochondrial DRP1 (Mito-DRP1) and cytoplasmic DRP1 (Cyto-DRP1) was analyzed by western blot. COX IV and β-tubulin were used as protein loading controls for mitochondria and cytoplasm, respectively (*n* = 4). **r** HL-1 cells were transfected with NCi (Con) and *Ddx17* siRNA (*Ddx17*-KD) for 24 h, then treated with PBS or 0.5 μM Dox for 24 h and divided into Con, *Ddx17*-KD, Con + Dox and *Ddx17*-KD + Dox groups. Cardiomyocyte mitochondria and cytoplasm were extracted separately using a mitochondrial isolation kit. Cytochrome c levels in mitochondria and cytoplasm were analyzed by western blot. COX IV was used as a protein loading control for mitochondria and β-tubulin as a protein loading control for cytoplasmic proteins (*n* = 4). *P < 0.05, **P < 0.01, and ***P < 0.001

To explore the molecular mechanisms by which DDX17 inhibits mitochondrial fission and promotes mitochondrial fusion, we applied the nucleosome occupancy and methylome sequencing (NOMe-seq) to NMVMs from *Ddx17*-Tg mice and controls. Based on the methylation levels of cytosines at WCG (ACG/TCG) sites according to the NOMe-seq data, we compared genome-wide promoter methylation profiles in cardiomyocytes between *Ddx17*-Tg mice and controls. The promoters of 175 hypermethylated genes and 114 hypomethylated genes were identified in *Ddx17*-Tg mice. The hypermethylated genes in *Ddx17*-Tg mice were mainly related to GO terms such as striated muscle tissue development, regulation of DNA metabolism process and the cellular response to oxidative stress; and the hypomethylated genes were mainly involved in chemokine and immune response (Fig. 5a, b). We then calculated chromatin accessibility based on cytosine methylation levels at GCH (GCA/GCT/GCC) sites. To map the open chromatin regions in the *Ddx17*-Tg mice and controls, we identified proximal nucleosome-depleted regions (NDRs) and searched for the binding motifs of TFs in these NDRs. Interestingly, only NDRs in *Ddx17*-Tg mice were enriched for binding motifs of STAT3, ELF4 and BCL6 (Fig. 5c).

Co-Immunoprecipitation (Co-IP) results showed that DDX17 could interact with the transcriptional repressor BCL6 in NMVMs (Fig. 5d, e). BCL6 is a well-known and important transcriptional regulator that represses >1200 target genes. Immunofluorescence co-localization of DDX17 and BCL6 in primary mouse cardiomyocytes also showed that they are both located in the nucleus of cardiomyocytes (Fig. 5f). Previous studies have shown that BCL6 plays an important role in the differentiation and development of hematopoietic stem cells by affecting both mitochondrial function and apoptosis.^22^ Bioinformatic analysis revealed potential BCL6 binding sites in the promoter region of the *Drp1* gene. We constructed expression plasmids for *Ddx17* (pcDNA-*Ddx17*) and *Bcl6* (pcDNA-*Bcl6*) using the pcDNA3.1 plasmid vector and the 0.8 kb wild-type *Drp1* promoter vector (pGL3-*Drp1*-WT) using the pGL3-basic plasmid vector (Supplementary Table 1). We tested the effect of DDX17 and/or BCL6 overexpression on *Drp1* promoter activity. Simultaneous overexpression of DDX17 and BCL6 significantly inhibited *Drp1* reporter luciferase activity, and the inhibition efficiency was significantly higher than that of BCL6 overexpression alone (Fig. 5g). Thus, DDX17 promoted the inhibitory effect of BCL6 on *Drp1* promoter activity. At the same time, we constructed a 0.8 kb *Drp1* promoter mutation plasmid (pGL3-*Drp1*-MUT) with mutated BCL6 binding sites using the pGL3-basic plasmid vector. Using this plasmid, overexpression of DDX17 alone or BCL6 alone and co-expression of DDX17 and BCL6 had no significant effect on *Drp1* promoter activity (Fig. 5h), indicating that the *Drp1* promoter region contains BCL6 binding sites. We next examined the inhibitory effect of BCL6 on *Drp1* promoter activity. HEK293A cells were cotransfected with wild-type *Drp1* promoter plasmid (pGL3-*Drp1*-WT) and different concentrations of *Bcl6* expression plasmid (0, 50, 100, 200, 300 ng/well), and it was found that BCL6 could inhibit *Drp1* promoter activity in a dose-dependent manner (Fig. 5i). Thus, these experiments confirmed that BCL6 binds to the promoter region of the *Drp1* gene and represses its transcription in a dose-dependent manner, whereas DDX17 can function as a transcriptional coregulator and promote the transcriptional repressive activity of BCL6 on *Drp1* in cardiomyocytes.

To further investigate the repression of *Drp1* transcription by BCL6, chromatin immunoprecipitation (ChIP) was performed on NMVMs using a BCL6 antibody. We found that the sequence from −449 bp to −436 bp of the transcription start site (TSS) of the mouse *Drp1* gene could be a transcription binding site for BCL6 (Supplementary Fig. 8b). The results showed that the amount of immunoprecipitated DNA (BCL6 binding sites in the *Drp1* promoter region) was significantly enriched by BCL6 antibody compared to control IgG (Fig. 5j). These results indicate that the BCL6 antibody can enrich the promoter region of the *Drp1* gene, suggesting that BCL6 can bind to the *Drp1* transcription start region and regulate the expression of the *Drp1* gene.

In addition, we performed rescue experiments by transfecting the cardiomyocyte cell line HL-1 with *Ddx17* siRNA and/or *Drp1* siRNA and then treated the cells with doxorubicin to analyze cell viability. Western blot results showed that *Ddx17* siRNA and *Drp1* siRNA effectively reduced DDX17 and DRP1 expression in cardiomyocytes. Dox treatment induced decreased expression of DDX17 and increased expression of DRP1. *Drp1* knockdown significantly inhibited the Dox-induced increase of DRP1 and cardiomyocyte apoptosis but did not alter the expression of *Ddx17* (Fig. 5k–n). Next, we knocked down *Ddx17* in HL-1 cardiomyocytes with *Ddx17* siRNA, isolated mitochondrial and cytoplasmic components of cardiomyocytes and analyzed DRP1 expression. Western blot assay showed that knockdown of *Ddx17* in basal condition promoted the expression of DRP1 in the cytoplasm, while in Dox treatment condition, knockdown of *Ddx17* simultaneously increased the expression of DRP1 in both cytosol and mitochondria at the same time, which promoted mitochondrial fission and cardiomyocyte injury (Fig. 5o–q). We also analyzed DRP1 expression in NMVMs from cardiomyocyte-specific *Ddx17*-knockout and cardiomyocyte-specific *Ddx17* transgenic mice and demonstrated that DDX17 could negatively regulate DRP1 expression (Supplementary Fig. 8c–f). Detection of myocardial cytochrome c (cyt c) expression by western blot revealed that *Ddx17* knockdown in HL-1 cardiomyocytes significantly increased the release of mitochondrial cytochrome c into the cytoplasm after Dox treatment (Fig. 5r, Supplementary Fig. 8g, h). These results suggest that the cardioprotective effect of DDX17 is mainly due to the regulation of DRP1 expression. *Ddx17* knockdown may contribute to the occurrence of mitochondrial homeostasis imbalance and cardiomyocyte dysfunction by increasing DRP1 expression, mitochondrial cytochrome c release and cell apoptosis.

Therefore, DDX17 coordinated with the transcriptional repressor BCL6 to repress *Drp1* gene transcription.

### DDX17 may be used in the diagnosis of heart failure and as a novel therapeutic target in clinical settings

**Fig. 6:**
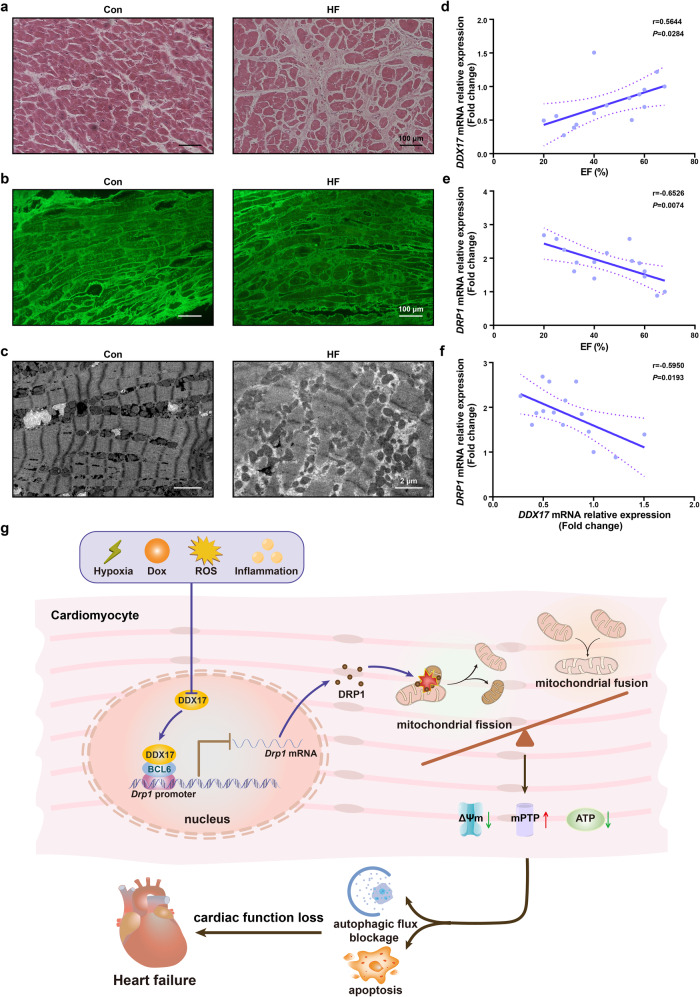
DDX17 expression levels are positively correlated with cardiac function in patients with different stages of heart failure. **a** H&E staining of myocardial tissue from the control (Con) and heart failure (HF) patients (*n* = 3); scale bar, 100 μm. **b** Wheat germ agglutinin staining of tissue samples isolated from Con and HF patients (*n* = 3); scale bar, 100 μm. **c** Transmission electron microscopy images of myocardial tissue from Con and HF patients (*n* = 3); scale bar, 2 μm. **d** Correlation of the *DDX17* mRNA levels with left ventricular EF in myocardial tissue from heart failure patients. **e** Correlation of *DRP1* mRNA levels with left ventricular EF in myocardial tissue from heart failure patients. **f** Correlation of *DDX17* with *DRP1* mRNA levels in the myocardium of heart failure patients. **g** Graphic summary of DDX17 protecting cardiac function by promoting mitochondrial homeostasis through the BCL6-DRP1 pathway in heart failure

To verify the correlation between the DDX17 level and heart failure and its clinical diagnostic value, we collected LV myocardial biopsy samples from 15 male patients (see Supplementary Table 2 for detailed clinical information) with different stages of heart failure. Consistent with the results observed in mouse models of heart failure, the cardiac tissue structure was disturbed in patients after heart failure, and the fibrosis level was significantly increased (Fig. 6a, b). In addition, to understand the changes in the mitochondrial structure at different stages of heart failure, we analyzed myocardial samples from patients with different heart functions by transmission electron microscopy. As cardiac function declined, the structural abnormalities of the mitochondria in cardiomyocytes became more apparent, and these abnormalities included a disordered mitochondrial arrangement, different sizes and morphologies, and disruption of cristae (Fig. 6c).

Using multivariate ANOVA correction, mRNA expression of *DRP1* or *DDX17* was not correlated with age in this small sample size of myocardial biopsy samples. Consistent with these structural changes, the left ventricular EF of patients with heart failure showed a significant positive correlation with the mRNA level of *DDX17* (*r* = 0.5644, *P* = 0.0284) and a negative correlation with the mRNA level of *DRP1* (*r* = −0.6526, *P* = 0.0074) in myocardial tissue (Fig. 6d, e). We also demonstrated that the mRNA level of *DDX17* was negatively correlated with that of *DRP1* in human heart failure samples (*r* = −0.5950, *P* = 0.0193) (Fig. 6f). Therefore, the expression of *DDX17* in myocardial biopsy samples may have important clinical application value in diagnosing and evaluating the stage of heart failure.

## Discussion

DDX17, a member of the DEAD-box RNA helicase family, is a nuclear and cytoplasmic shuttle protein. DDX17 is involved in almost all RNA metabolic processes, such as mRNA alternative splicing, miRNA and ribosome biosynthesis, mRNA degradation, interactions with lncRNAs, and regulators of transcriptional activity.^3,23^ Studies conducted in recent years have shown that DDX17 has a transcriptional regulatory function and can bind to various TFs to promote or inhibit the regulation of TFs on downstream genes.^3,4,23^ Studies in tumors have found that DDX17 has a wide range of biological functions, such as regulating cellular oxidative stress, autophagy, and apoptosis.^3^ Many studies have found that abnormal DDX17 expression is closely correlated with tumorigenesis and tumor development.^3,5^ In addition, reduced DDX17 expression may lead to defective growth and differentiation of brain development in fetal brains with Down syndrome.^24^

Previous studies have shown that DDX17 can inhibit doxorubicin-induced cardiomyocyte injury in vitro and is involved in the pathogenesis of cardiac hypertrophy by regulating the expression of the long non-coding RNA cardiac physiological hypertrophy-associated regulator.^6,7^ Although DDX17 is highly expressed in cardiomyocytes, the function of DDX17 in the cardiovascular system, particularly in the development of heart failure, is still not fully understood. Therefore, the aim of this study was to investigate the role of DDX17 in the maintenance of normal cardiac function and in the pathological conditions of cardiomyocyte injury and heart failure. Our study found that DDX17 expression was positively correlated with left ventricular EF in myocardial biopsy samples from heart failure patients and in Dox-induced acute heart failure and TAC-induced chronic heart failure mouse models. Next, using a *Ddx17*-cKO mouse model, we found that *Ddx17* cardiomyocyte knockout could reduce cardiac function, suggesting that DDX17 may be involved in maintaining normal cardiac function under physiological conditions. Under the pathological conditions caused by various stimuli, cardiomyocyte-specific *Ddx17* knockout can aggravate the loss of cardiac function, myocardial fibrosis and myocardial remodeling, resulting in heart failure. Interestingly, the cardiomyocyte-specific overexpression of *Ddx17* can restore DDX17 expression levels and attenuate cardiomyocyte injury and cardiac dysfunction under pathological conditions, demonstrating a significant cardioprotective effect.

Heart failure is a process in which cardiac function gradually changes from compensation to decompensation, and its mechanisms are mainly myocardial energy metabolism disorders and myocardial systolic/diastolic dysfunction.^25^ When cardiomyocytes are subjected to various injuries (oxidative stress, hypoxia-reoxygenation, doxorubicin), cardiomyocyte autophagy is significantly increased and autophagic flux is blocked.^20,26^ Damaged organelles, abnormal folding and dysfunctional proteins are unable to form normal autophagy‒lysosomes, further exacerbating myocardial mitochondrial damage and oxidative phosphorylation dysfunction, thereby increasing the level of myocardial apoptosis and causing irreversible damage to cardiac function.^27,28^ In recent years, the regulation of autophagy homeostasis and the inhibition of apoptosis have become new directions to alleviate myocardial injury and inhibit heart failure.^29,30^ Our study found that cardiomyocyte-specific *Ddx17* knockout resulted in blockage of autophagic flux, mitochondrial dysfunction and cardiomyocyte apoptosis. DDX17 homeostasis can promote cardiac function and protect cardiomyocytes from many pathological stimuli. Therefore, regulating the expression of DDX17 in cardiomyocytes, maintaining mitochondrial homeostasis and normal autophagy function, and reducing cardiomyocyte apoptosis under pathological conditions are potential clinical diagnosis and treatment targets for myocardial injury and heart failure.

BCL6 is a special transcriptional repressor that was originally thought to regulate B lymphocyte development and growth.^8,9^ BCL6 acts on multiple targets, including signal transducers, transcriptional activators (STAT3 and STAT5B), and microRNAs, promoting the development of many cancers.^12,31^ BCL6 binds to specific DNA sequences in the promoter regions of target genes and exerts transcriptional repression, that regulates cell proliferation, cell differentiation, DNA damage repair, maintenance of genome stability, and the cell cycle.^9^ Inhibition of BCL6 function can lead to cell cycle arrest, gene instability and apoptosis.^14,32^ In the cardiovascular system, BCL6 can inhibit doxorubicin-induced cardiomyocyte senescence by activating PPARδ.^13^ Other research teams have also found that Bcl6 knockout mice develop severe Th2-type inflammatory diseases, including myocarditis and pulmonary vasculitis, suggesting that BCL6 negatively regulates the inflammatory response in vivo.^15^ In addition, BCL6 may play an important role in the pathophysiological process of ischemia-hypoxic injury in cardiomyocytes and protect cardiomyocytes by inhibiting oxidative stress, inflammation, and cell apoptosis.^16,33,34^ However, the regulatory mechanisms of BCL6 in cardiomyocytes are not fully understood. In this study, using the mouse heart failure model and the cardiomyocyte injury model, DDX17 was found to bind to BCL6 and jointly participate in inhibiting the expression of the downstream gene *Drp1*, which results in blocking the excessive fission of myocardial mitochondria and maintaining their homeostasis and function. When cardiomyocytes are injured, the expression of DDX17 is decreased, and the transcriptional repressive function of BCL6 is also decreased. This in turn leads to abnormally elevated DRP1 expression, increased mitochondrial fission, and disruption of normal mitochondrial homeostasis, resulting in cardiomyocyte loss and reduced cardiac function. Thus, under pathological conditions, aberrantly downregulated DDX17 expression can lead to reduced BCL6 transcriptional repression, resulting in cardiomyocyte injury and the development of heart failure.

Our study found that DDX17 is involved in the regulation of mitochondrial homeostasis in cardiomyocytes mainly through the regulation of DRP1 expression and mitochondrial fission, which are involved in the maintenance of normal cardiac function and inhibition of heart failure. The high-capacity mitochondrial system in cardiomyocytes is dynamically regulated, producing and consuming large amounts of ATP to support the pumping function of the heart. The coupling between contractions also generates a large amount of reactive oxygen species. However, cardiomyocytes have a number of regulators of mitochondrial fusion, fission and mitophagy that protect cardiomyocyte function through quality control mechanisms. However, impaired function or aberrant expression of these regulators can lead to mitochondrial dysfunction in cardiomyocytes, resulting in cardiomyocyte dysfunction.^18,35^ Thus, the mitochondrial quality control system is essential for the maintaining of mitochondrial function. At the organelle level, mitochondrial mass is maintained by mitochondrial synthesis, fusion and fission, and removal of damaged mitochondria.^36^ In experiments with pressure overload-induced myocardial hypertrophy and heart failure mouse model, the results showed that the increased autophagy in response to pressure overload occurs concurrently with DRP1 mitochondrial translocation, and upregulation of DRP1 aggravates pressure overload induction of cardiac dysfunction.^37^ Excessive mitochondrial fission is mainly caused by the upregulation or activation of DRP1. During myocardial ischemia/reperfusion injury, the increased expression and activation of DRP1 can cause increased mitochondrial fission and disruption of mitochondrial homeostasis, leading to cell apoptosis and loss of cardiac function.^17,38^ Our study also revealed that DDX17/BCL6-DRP1 is a novel regulatory pathway of mitochondrial homeostasis and cardiac function with important functions in maintaining cardiomyocyte function under physiological conditions and inhibiting the development of heart failure under pathological conditions (Fig. 6g).

In conclusion, our study suggests that DDX17 protects cardiac function by promoting mitochondrial homeostasis through the BCL6-DRP1 pathway. Abnormal DDX17 expression may be one of the common pathways of myocardial injury and heart failure, and DDX17 may also be potentially valuable for the diagnosis and treatment of heart failure induced by a variety of pathological factors in clinical practice.

## Materials and methods

### Ethics approval statements

All animal experiments were performed according to the protocols (LA2021576) approved by the Laboratory Animal Welfare Ethics Branch of Peking University Biomedical Ethics Committee Institutional Animal Care Program and adhered to the Guide for the Care and Use of Laboratory Animals (NIH Publication #85-23, revised 1996) and ARRIVE guidelines.

For human samples, the study protocol (2018BJYYEC-121-03) was approved by the Ethics Committee of Beijing Hospital and conformed to the principles outlined in the Declaration of Helsinki for the Use of Human Tissue or Subjects. Written informed consent was provided by all participants. The data were anonymized and deidentified prior to analysis.

### Endomyocardial biopsy (EMB)

EMB samples were obtained from 15 male patients with cardiomyopathy at Beijing Hospital. A modified Seldinger method was used to puncture the right radial artery. EMB was performed using the radial approach reported previously.^39^ All myocardial biopsy specimens were stored in liquid nitrogen immediately after the procedure until total RNA was extracted or placed in fixative solution for immunofluorescence and transmission electron microscopy analysis. Detailed information on all patients, such as age, sex, cardiac function, brain natriuretic peptide (BNP), and diagnosis, is presented in Supplementary Table 2.

### Mouse TAC-induced chronic heart failure

TAC was performed on 10 week-old male C57BL/6 J mice purchased from SPF (Beijing) Biotechnology Co., Ltd. in China. Sham-operated mice underwent identical procedures except for aortic constriction. Twelve weeks after TAC, mice were anesthetized with 3-4% isoflurane and euthanized by cervical dislocation, and the hearts were harvested for further analysis as previously reported.^40^

### Mouse model of doxorubicin-induced acute heart failure

Ten-week-old male C57BL/6 J mice were used in a model of doxorubicin-induced heart failure. Doxorubicin was purchased from Cell Signaling Technology (Cat. No. 5927, USA) and dissolved in saline. Doxorubicin (7.5 mg/kg) or an equivalent volume of saline was administered to mice by intraperitoneal injection every other day for a total of three injections. On day 6 after the first doxorubicin injection, the mice were euthanized and heart samples were collected.

### Generation of cardiomyocyte-specific *Ddx17*-Tg mice

*Ddx17*-Tg mice were generated using CRISPR/Cas9 techniques. The *Ddx17* (Gene ID: 67040) transgenic construct was generated by cloning the *Ddx17* cDNA into an expression vector containing the *α-MHC* promoter. The *Ddx17*-Tg construct was injected into 1-cell embryos of C57BL/6 J mice. The transgenic founders were further crossed with C57BL/6 J mice. Age-matched male *Ddx17*-Tg mice and their wild-type (WT) littermates (C57BL/6 J) were used in this study.

### Generation of cardiomyocyte-specific *Ddx17*-knockout mice

The *α-MHC-Cre* mice were obtained from the Jackson Laboratory. For conditional knockout, *loxP* fragments were inserted into exon 3 (ENSMUST00000054014.9) using the CRISPR/Cas9 method. Heterozygous *Ddx17*^*fl/+*^ mice were backcrossed to generate homozygous *Ddx17*^*fl/fl*^ mice. To generate cKO mice (*α-MHC-Cre/Ddx17*^*f/f*^), *Ddx17*^*fl/fl*^ mice were crossed with *α-MHC-Cre* mice. Control male mice with the *Ddx17*^*fl/fl*^ *α-MHC-Cre* negative or *α-MHC-Cre/Ddx17*^*+/+*^ genotype were subjected to the same treatment.

### Echocardiography

Mice were anaesthetized with 1%-1.5% isoflurane in oxygen and body temperature was maintained with a heating pad. Cardiac function was measured at a steady heart rate of 400–500 beats/min. Echocardiography was performed by investigators using a Vevo3100 imaging system (VisualSonics, Canada) and was blinded to animal group, as previously reported.^40^

### Histology, immunofluorescence and immunohistochemistry

Tissues were processed to obtain paraffin-embedded sections and then analyzed by hematoxylin and eosin staining according to the manufacturer’s protocol (Cat. No. G1100, Solarbio, China). For immunofluorescence analysis, 7 μm sections were incubated with primary antibodies overnight at 4 °C, washed with 0.25% Triton X-100 in PBS, and incubated with fluorescence-labeled secondary antibodies for 2 h. Sections were imaged using a GENERTION2 microscope (Discover Echo Inc., USA).

Cardiomyocytes were rinsed with PBS, fixed with 4% paraformaldehyde for 10 min and then permeabilized with 0.1% Triton X-100 for 10 min. The cells were incubated with rabbit anti-DDX17 antibody for 12 h. The cells were washed three times with PBS for 5 min and then mouse anti-BCL6 antibody was added for 12 h. The cells were then incubated with goat anti-rabbit secondary antibody (red) and goat anti-mouse secondary antibody (green) for 1 h at 37 °C. The nuclei were then counterstained with Hoechst 33342 (Cat. No. 875756-97-1, Merck, USA) or DAPI (Cat. No. D9542, Merck, USA). Cardiomyocytes were then examined by fluorescence microscopy (Olympus BX51, USA). Images were merged using Adobe Photoshop CS6 (Adobe Systems, USA).

### Quantitative real-time PCR

Total RNA was extracted from heart tissue and cardiomyocytes using TRIzol reagent (Cat. No. 10296028CN, Invitrogen, USA). cDNA was synthesized from equal amounts of total RNA from each sample using a cDNA First Strand Synthesis Kit (Cat. No. E6300L, New England Biolabs, USA). Quantitative real-time PCR was performed using reaction mixture containing SYBR Green (Cat. No. RR8820A, TaKaRa, Japan). All primers were synthesized by Sangon Biotech in China. The mouse primers used in the experiment included the following: *Gapdh*, 5′-AACTTTGGCATTGTGGAAGG-3′ and 5′-ACACATTGGGGGTAGGAACA-3′; *Ddx17*, 5′-TATGGAAGCCCAAATTCTGC-3′ and 5′-CAGATCGGCCTATCCCACTA-3′; *Nppa*, 5′-GATAGATGAAGGCAGGAAGCCGC-3′ and 5′-AGGATTGGAGCCCAGAGTGGACTAGG-3′; *Nppb*, 5′-ATTCAAGATGCAGAAGCTG-3′ and 5′-GAATTTTGAGGTCTCTGCTG-3′; *Col1a1*, 5′-TGACTGGAAGAGCGGAGAGT-3′ and 5′-GACGGCTGAGTAGGGAACAC-3′; *Col3a1*, 5′-CGTAAGCACTGGTGGACAGA-3′ and 5′-AGCTGCACATCAACGACATC-3′. The following human primers were used: *GAPDH*, 5′-CCATGGGGAAGGTGAAGGTC-3′ and 5′-GCGCCCAATACGACCAAATC-3′; *DDX17*, 5′-TGGACGAAGCTGACAGAATG-3′ and 5′-GCCTACGTTGATCTGGGTGT-3′; *DRP1*, 5′-GATGCCATAGTTGAAGTGGTGAC′ and 5′-CCACAAGCATCAGCAAAGTCTGG-3′. Quantitative PCR were performed as previously reported.^41^

### Cell lines, isolation of NMVMs, and cell culture condition

HEK293A was purchased from the American Type Culture Collection (ATCC), and cultured in Dulbecco’s modified Eagle’s medium (Cat. No. D5796, Sigma-Aldrich, USA), supplemented with 10% fetal bovine serum (FBS, Cat. No. SH30071, Hyclone, USA) and antibiotics (100 U/mL penicillin G and 0.1 mg/mL streptomycin). The adult murine cardiomyocyte cell line HL-1 was purchased from Sigma-Aldrich, and cultured in Claycomb’s medium (Cat. No. 51800 C, Sigma-Aldrich, USA), supplemented with 10% fetal bovine serum and antibiotics.

NMVMs were isolated from 1- to 2-day-old C57BL/6 J mice, *Ddx17*-Tg and *Ddx17-*cKO mice. Prior to isolation of NMVMs from neonatal *Ddx17*-Tg and *Ddx17*-cKO mice, rapid mouse tail gene genotyping was performed and cardiomyocytes were grouped according to genotype. Neonatal mice were euthanized by decapitation. Cardiomyocytes were plated at a density of 6.6 × 10^4^ cells/cm^2^ in a cell culture medium consisting of Dulbecco’s modified Eagle’s medium and Medium 199 (Cat. No. M2154, Sigma-Aldrich, USA) at a volume ratio of 3:1, and with supplemented with 5% fetal bovine serum and 10% equine serum (Cat. No. SH30074, Hyclone, USA) in the presence of 0.1 mM 5-bromo-2-deoxyuridine (Cat. No. B5002, Sigma‒Aldrich, USA) and antibiotics (100 U/mL penicillin G and 0.1 mg/mL streptomycin).^41^ All the cells were cultured at 37 °C in an incubator with 5% CO_2_ and 95% air (v/v).

### Adenovirus transfection

Recombinant adenovirus containing mouse *Ddx17* cDNA (NM_199080.2) was prepared using the AdEasy adenovirus vector system from Agilent (USA) as previously reported.^42^ HL-1 cells or NMVMs were transfected with empty vector adenovirus (Con) or *Ddx17*-overexpressing adenovirus (*Ddx17*-OE) at a multiplicity of infection (M.O.I.) of 30 for 24 h.

### Cardiomyocyte hypoxia culture

The NMVMs or cardiomyocyte cell line HL-1 cells were cultured in hypoxic medium and placed in a hypoxic chamber equilibrated with 94% nitrogen, 5% carbon dioxide, and 1% oxygen (v/v/v) for 16 h. The composition of the hypoxic medium was 125 mM NaCl, 8 mM KCl, 1.2 mM KH_2_PO_4_, 1.25 mM MgSO_4_, 1.2 mM CaCl_2_, 6.25 mM NaHCO_3_, 5 mM sodium lactate and 20 mM HEPES.

### Detection of ATP levels in cardiomyocytes

Intracellular ATP levels were determined using a UV spectrophotometer assay kit (Cat. No. BC0300, Solabio, China).^43^ Collect 1 × 10^4^ cells and add 1 mL extraction buffer, sonicate for 1 min on ice. Then centrifuge the cells at 1 × 10^4^g for 10 min at 4 °C, and measure ATP in the supernatant according to the manufacturer’s recommendations. Luminescence intensity was normalized to the total amount of protein.

### Assessment of mitochondrial membrane potential by JC-1 in HL-1 cells

At high mitochondrial membrane potential, JC-1 (Cat. No. C2006, Beyotime, China) can produce red fluorescence; at low mitochondrial membrane potential, JC-1 can produce green fluorescence.^43^ Cardiomyocytes were incubated with 2 μg/mL JC-1 at 37 °C for 20 min. The ratio of red fluorescence to green fluorescence of cardiomyocytes was observed by fluorescence microscopy and calculated using Image J software.

### Determination of mitochondrial permeability transition pore (mPTP) opening in HL-1 cells

Live cells can be stained with the fluorescent probe calcein acetoxymethyl ester (calcein AM) and CoCl_2_ to analyze mitochondrial permeability transition pore. Opening of the mPTP allows free efflux of calcein and influx of Co^2+^ from the mitochondrial membrane, resulting in a reduction of calcein fluorescence intensity in the mitochondria.^43^ Cardiomyocytes were incubated with 1 μM calcein-AM and 2 mM CoCl_2_ (Cat. No. C2009S, Beyotime, China) for 20 min at 37 °C in the dark. Images were captured by fluorescence microscopy after washing with PBS and quantified using Image J software.

### Terminal deoxynucleotidyl transferase-mediated dUTP nick-end labeling (TUNEL) staining

TUNEL staining (Cat. No. 4810-30-CK, R&D System, USA) of heart sections: TUNEL staining of heart sections was performed according to previously reported methods with modifications. Briefly, heart sections were fixed in 4% paraformaldehyde for 20 min at room temperature and TUNEL staining was performed using the Apoptosis Detection Kit. The rate of apoptotic nuclei was determined in 800–1000 cells in 10 randomly selected fields of view in each culture dish.

TUNEL staining of cultured cardiomyocytes was performed by a previously reported method, briefly: cultured cardiomyocytes were grown on slides, fixed in 4% paraformaldehyde for 20 min at room temperature and TUNEL stained according to the instructions of the Roche Cell Death Assay Kit (Cat No. 12156792910, Roche, Switzerland). Cell nuclei were counterstained with 10 mM Hoechst 33342 for 2-3 min and then visualized under a fluorescence microscope. The percentage of TUNEL-positive cells was calculated from 10 randomly selected areas of cells from each dish.

### Co-Immunoprecipitation (Co-IP)

Co-IP was performed using a Pierce Classic IP Kit (Cat. No. 26149, Thermo Scientific, USA) after the manufacturer’s instructions. NMVMs (10-cm dishes; 5 × 10^6^ cells/dish; 1 dish per IP) were used for each reaction. Proteins were separated by sodium dodecyl sulfate-polyacrylamide gel electrophoresis.

### Nucleosome occupancy and methylome sequencing (NOMe-seq)

NMVMs from *Ddx17*-Tg and WT mice were lysed in lysis buffer (10 mM Tris·HCl at pH 7.5, 10 mM NaCl, 3 mM MgCl_2_, 0.1 mM EDTA, and 0.5% NP-40) containing phenylmethyl sulfonyl fluoride and a protease inhibitor cocktail for 60 min. Genomic DNA was purified by phenol: chloroform: isoamyl alcohol extraction and ethanol precipitation. First- and second-strand synthesis was performed using the random Oligo1 (5′-biotin-CTACACGACGCTCTTCCGATCTNNNNNNNNN-3′) and Oligo2 (5′-AGACGTGTGCTCTTCCGATCTNNNNNNNNN-3′) primers, respectively. The final NOMe-seq libraries were amplified through 12 cycles of PCR using KAPA HiFi Hot Start Ready Mix (Cat. No. KK2602, KAPA Biosystems, USA) and sequenced on the HiSeq 4000 platform (Novogene, China). The NOMe-seq data was processed based on previous study.^44^

### Chromatin immunoprecipitation (ChIP) analysis

ChIP analyses were performed using the SimpleChIP® Enzymatic Chromatin IP Kit (Magnetic Beads) (Cat. No. 9003S, Cell Signaling Technology, USA) and BCL6 antibody. A total of 5×10^6^ NMVMs were used per reaction. Real-time quantitative PCR primers were designed based on the binding site of the transcriptional repressor BCL6 in the *Drp1* promoter region (5′-GGTTCGAGACAGGTTTCT-3′, 5′-GCAGGCGAATTTCTGAGTTC-3′). Immunoprecipitated DNA from each sample was purified and the binding efficiency of TF to the regulatory region of the transcriptional promoter of the *Drp1* gene was analyzed by real-time quantitative PCR. The amount of immunoprecipitated DNA in each sample was expressed as the amount of signal relative to the total amount of input chromatin (equivalent to 1).^45^

### Plasmids construction, co-transfection and luciferase assays

Luciferase assays were performed using the pGL3-basic promoter reporter construct as previously reported.^46^ Mus musculus *Bcl6* and *Ddx17* expression plasmids were constructed using the pcDNA3.1 plasmid vector. For co-transfection and luciferase assays, HEK-293A cells were grown in 24-well plates (70-80% confluence) and transfected with 400 ng/well of the pGL3-*Drp1* promoter reporter construct plasmid (pGL3-*Drp1*-WT) or the *Drp1* promoter reporter gene plasmid mutated with the BCL6 binding site (pGL3-*Drp1*-MUT) (Supplementary Table 1), 300 ng of pcDNA-*Bcl6* (Mus musculus *Bcl6*, NM_001348026.2) and/or 300 ng pcDNA-*Ddx17* (Mus musculus *Ddx17*, NM_199080.2) expression plasmids, and 50 ng Renilla luciferase reporter plasmid pRL-TK using VigoFect (Cat. No. T001, Vigorous Biotechnology, China) according to the manufacturer’s instructions. The pGL3-*Drp1* promoter-reporter plasmid (pGL3-*Drp1*-WT) was cotransfected with different doses (50, 100, 200, 400 ng) of *Bcl6* expression plasmid (pcDNA-*Bcl6*) in HEK-293A cells. In all these cotransfection experiments, pcDNA3.1 vector plasmids were used as equilibrium plasmids in different transfection mixtures to equalize the total amount of DNA. Luciferase assays were performed using a dual luciferase assay kit (Cat. No. E1960, Promega, USA) according to the manufacturer’s instruction.^46^

### Transfection of siRNA

Negative control (NCi, Cat. No. sc-37007, Santa Cruz, USA), mouse *Ddx17* siRNA (Cat. No. sc-142922, Santa Cruz, USA) and *Drp1* siRNA (Cat. No. sc-45953, Santa Cruz, USA) were purchased from Santa Cruz Co. When the cell density reached 70%, NCi and *Ddx17* siRNA or *Drp1* siRNA were transfected into HL-1 cells using RNAiMax Transfection Reagent (Cat. No. 13778150, Thermo Fisher, USA). NCi was used as the equilibrium RNA in the different transfection mixtures to balance the total amount of RNA. After 24 h of transfection, the old medium was removed from the transfected cells, replaced with fresh medium, and treated with PBS or Dox for 24 h, and the analysis was performed as previously reported.^41^

### Western blot

Western blot was performed using antibodies against BCL2-associated X protein (BAX), B-cell lymphoma 2 (BCL2), BCL6, caspase-3 (CASP3), cleaved-caspase-3 (c-CASP3), COX IV, cytochrome c (cyt c), DDX17, DRP1, FIS1, microtubule-associated protein 1 light chain 3 beta (LC3B), MFF, MFN1, MFN2, OPA1, p62 (detailed information in Supplementary Table 3). β-tubulin was used as a cytoplasmic protein loading control. COX IV was used as a mitochondrial protein loading control. GAPDH antibody was used as a total cell protein loading control for normalization.^47^ Briefly, cultured cells were lysed in lysis buffer and tissue samples were lysed using RIPA. Samples were subjected to SDS-PAGE and transferred to PVDF membranes. Blots were probed with primary antibodies. The blot was then probed with horseradish peroxidase-conjugated secondary antibodies. Antigen-antibody complexes were detected using enhanced chemiluminescence reagent. Finally, quantitative analysis was performed using ImageJ software (http://rsb.info.nih.gov/ij/) as previously reported.^47^ Each cell experiment may contain 1–6 parallel cell samples, which are averaged to represent the results of a single experiment for statistical analysis.

### Mitochondrial oxygen consumption rate (OCR) analysis

Mitochondrial OCR analysis was performed after optimization of the experiments according to the manufacturer’s instructions (Seahorse Bioscience, USA) with reference to previously published literature. Briefly, mouse atrial myocyte HL-1 cells (5 × 10^4^ cells per well) were seeded on XF24 cell culture microtiter plates (Cat. No. 101122-100, Seahorse Bioscience, USA) and incubated in Claycomb’s medium. After 24 h of culture, fetal bovine serum was removed and the control adenovirus and the *Ddx17* overexpression adenovirus were transfected separately for 24 h at 37 °C in a CO_2_-humidified incubator, followed by treatment with 0.4 μM Dox for 24 h. The sensors in the sensor cassette were hydrated with XF calibration solution for 18 h. A solution containing 10 mM D-glucose, 1 mM sodium pyruvate and 2 mM L-glutamine was added to XF Base DMEM. The treated cells were washed twice with the prepared XF Base DMEM medium and then equilibrated for 60 min at 37 °C in a CO_2_-free incubator. The sensor cassette ports were loaded with 1.0 μM oligomycin, 1.0 μM FCCP and 0.5 μM rotenone/antimycin A (Rot/AA). We used the XF Cellular Mitochondrial Stress Test Kit (Cat. No. 103010-100, Seahorse Bioscience, USA) to assess mitochondrial OCR and calculated OCR using Wave software (Seahorse Bioscience).^48^

### Measurement of lactate dehydrogenase (LDH) activity and creatine kinase-MB (CK-MB) level

After the mice were anesthetized, blood was collected from the right ventricle. Whole blood was allowed to stand at room temperature for 1 h and then centrifuged at 4 °C for 15 min at 1000 g to obtain serum. Serum levels of LDH and CK-MB were measured using the following mouse ELISA kits: Lactate Dehydrogenase Assay Kit (Cat. No. S03034, Rayto Life and Analytical Sciences, China) and Creatine Kinase Isozyme Kit (Cat. No. C060, Changchun Huili Biotech, China). All ELISA experiments were performed according to the manufacturer’s protocol.

### Cytoplasmic and mitochondrial extraction and protein immunoblotting

Cytoplasmic and mitochondrial fractions were prepared according to the instructions of the Mitochondrial Extraction Kit (Cat. No. SM0020, Solarbio, China). Briefly, cardiomyocytes were digested with trypsin, washed 3 times with PBS and centrifuged at 800 g for 10 min to collect cells. For each extraction, 1.0 mL of ice-cold lysis buffer was added to 5 × 10^7^ cells to resuspend the cells. The cell suspension was ground 30–40 times with a glass homogenizer in a 0 °C ice bath. The homogenate was centrifuged twice at 1000 g for 5 min at 4 °C to collect nuclei and debris. The supernatant was centrifuged at 12,000 g for 10 min at 4 °C to obtain the cytoplasmic and mitochondrial components. The mitochondrial component was resuspended in cell lysate to obtain the mitochondrial protein fraction. For protein immunoblotting, COX IV and β-tubulin were used as protein loading controls as mitochondrial and cytoplasmic protein loading controls, respectively.^49^

### Mitochondrial staining and analysis of mitochondrial fission

Mitochondrial staining was performed as previously described with modifications.^49,50^ Briefly, cardiomyocytes were cultured on coverslips coated with 0.01% poly-L-lysine. After treatment, they were stained with 50 nM Mito-Tracker Red CMXRos (Cat. No. C1035, Beyotime, China) for 15 min. Cardiomyocytes were mounted with anti-fade mounting medium. Images were analyzed with a Nikon A1 immunofluorescence microscope. Mitochondrial morphological changes were assessed by single-blind quantification of confocal microscopy images, with at least 4 repetitions of the experiment, each assessing mitochondrial morphology of ~100–140 randomly selected cells. Cardiomyocytes with >50% punctate mitochondrial morphology were considered mitochondrial fission positive cells. The mitochondrial fission rate was defined as the number of mitochondrial fission positive cells divided by the total number of cells.

### Statistical analysis

Data are presented as mean ± SEM. Statistical analysis was performed using GraphPad Prism 8.0.1. The Kolmogorov-Smirnov test was used to test the normality of the distribution of the data sets. Student’s *t*-test was used to compare two groups of data. Comparisons between multiple groups were performed using one-way ANOVA followed by Bonferroni’s multiple comparison post hoc test. Data differences between groups were separated into two independent factors using two-way ANOVA followed by Tukey’s post hoc tests. Changes in mouse survival were statistically evaluated using the log-rank (Mantel-Cox) test. Correlation analysis was used to analyze the correlation between two groups of data. *P* < 0.05 was considered statistically significant.

### Supplementary information

Supplementary Materials

Original image of Western blot

## Data Availability

The NOMe-seq raw sequencing data and processed data have been deposited in the Gene Expression Omnibus (GEO) under the accession code GSE157135. In the processed data, file names including “WCG” correspond to the DNA methylation levels, and file names including “GCH” correspond to the chromatin accessibility levels. All data needed to evaluate the conclusions described in the paper are presented in the paper and/or in the Supplementary Material. Additional data related to this paper can be requested from the corresponding authors.
