# Supplementary material for: DEAD-box helicase 17 (DDX17) protects cardiac function by promoting mitochondrial homeostasis in heart failure
Source: Signal Transduct Target Ther. 2024 May 24;9:127. doi: 10.1038/s41392-024-01831-2 (PMC11116421; doi:10.1038/s41392-024-01831-2)
Supplement: Supplementary file 1 — Supplementary Materials [file 41392_2024_1831_MOESM1_ESM.docx]

Supplementary Materials for

DEAD-box helicase 17 (DDX17) protects cardiac function by promoting mitochondrial homeostasis in heart failure

Mingjing Yan^#^, Junpeng Gao^#^, Ming Lan^#^, Que Wang^#^, Yuan Cao^#^, Yuxuan Zheng, Yao Yang, Wenlin Li, Xiaoxue Yu, Xiuqing Huang, Lin Dou, Bing Liu, Junmeng Liu, Hongqiang Cheng, Kunfu Ouyang, Kun Xu, Shenghui Sun, Jin Liu, Weiqing Tang, Xiyue Zhang, Yong Man, Liang Sun, Jianping Cai, Qing He, Fuchou Tang^*^, Jian Li^*^, Tao Shen^*^

Correspondence to: Tao Shen (shentao4189@bjhmoh.cn) or Jian Li (lijian@bjhmoh.cn) or Fuchou Tang (tangfuchou@pku.edu.cn)

**This PDF file includes:**

Supplementary Fig. 1 to Supplementary Fig. 8

Supplementary Table 1 to Supplementary Table 3


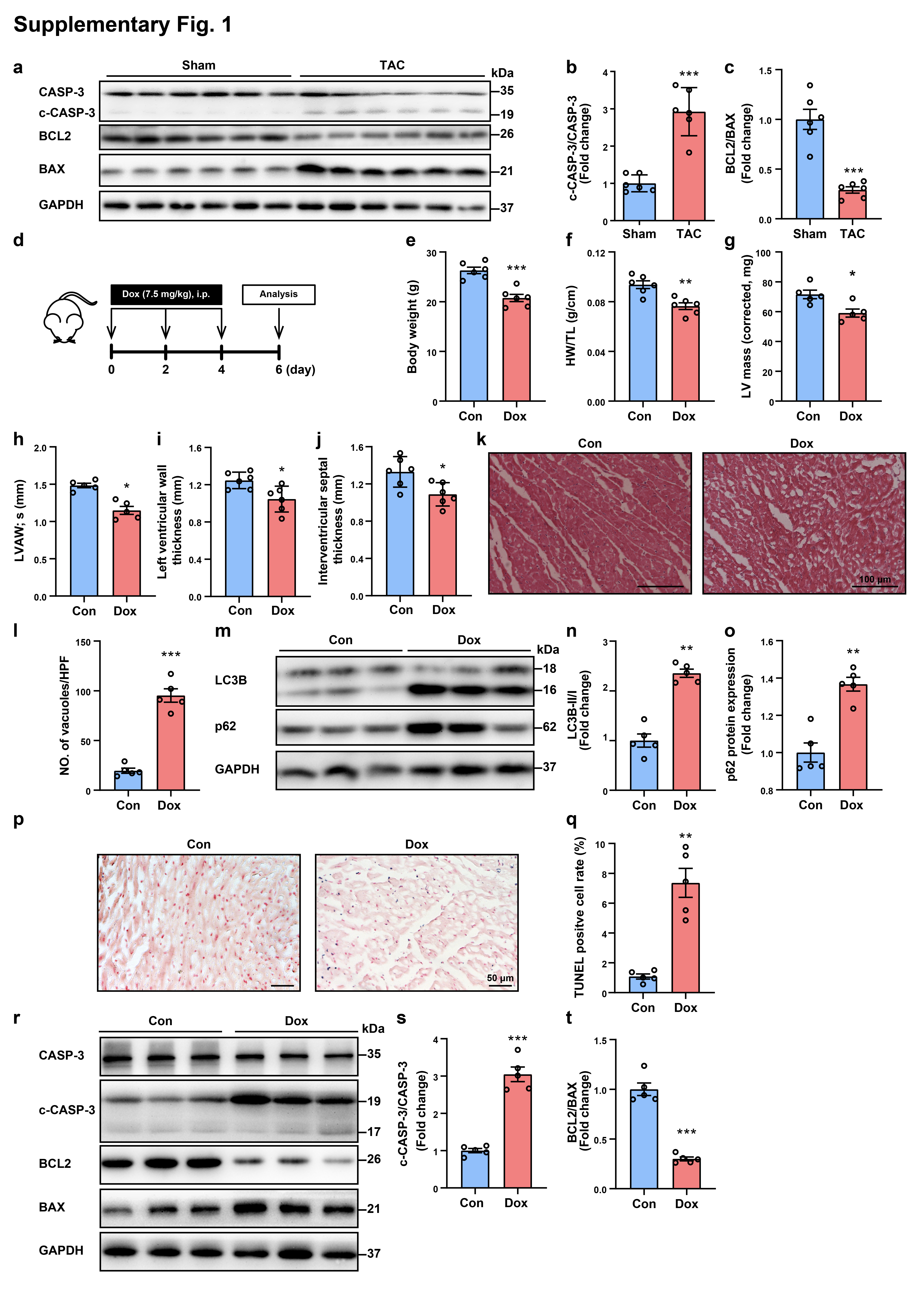


Supplementary Fig. 1. Dox induces autophagic flux blockage and cell apoptosis in mouse heart tissue.

**a-c** Western blot and the average data of the expression of apoptosis-related proteins such as CASP-3, c-CASP-3, BCL2 and BAX in the left ventricular myocardial tissue from sham-operated (Sham) mice and TAC-induced (TAC) heart failure mouse models (n=6). **d** Schematic outline of the experimental protocol for establishing the Dox-induced heart failure mouse model. 10-week-old male C57BL/6J mice were injected intraperitoneally with saline (Con) or 7.5 mg/kg Dox (Dox) 3 times every other day. **e-f** Mouse body weight **(e)** and heart weight (HW) and tibia length (TL) ratio **(f)** of the control (Con) and Dox-treated (Dox) mice (n=6). **g-h** Average data of the echocardiographic parameters of control (Con) and Dox-treated (Dox) mice: LV mass, LVAW;s (n=5). **i-j** Left ventricular wall thickness and interventricular septum thickness between Con and Dox groups (n=6). **k-l** Representative images of H&E-stained heart sections from mice in the Con and Dox groups and quantification of cytoplasmic vacuolation (n=5). **m-o** Western blot and the average data of the expression of the autophagy-related proteins LC3B and p62 in the myocardium of the Con and Dox groups of mice (n=5). **p-q** Representative images and the average data of TUNEL staining showing the apoptotic cells in the heart sections of the Con and Dox groups (n=5). **r-t** Western blot and statistical analysis of the expression of apoptosis-related proteins such as CASP-3, c-CASP-3, BCL2 and BAX in the left ventricular myocardial tissue of Con and Dox-treated mice (n=5). * *P*< 0.05, ** *P*< 0.01, and *** *P*< 0.001.


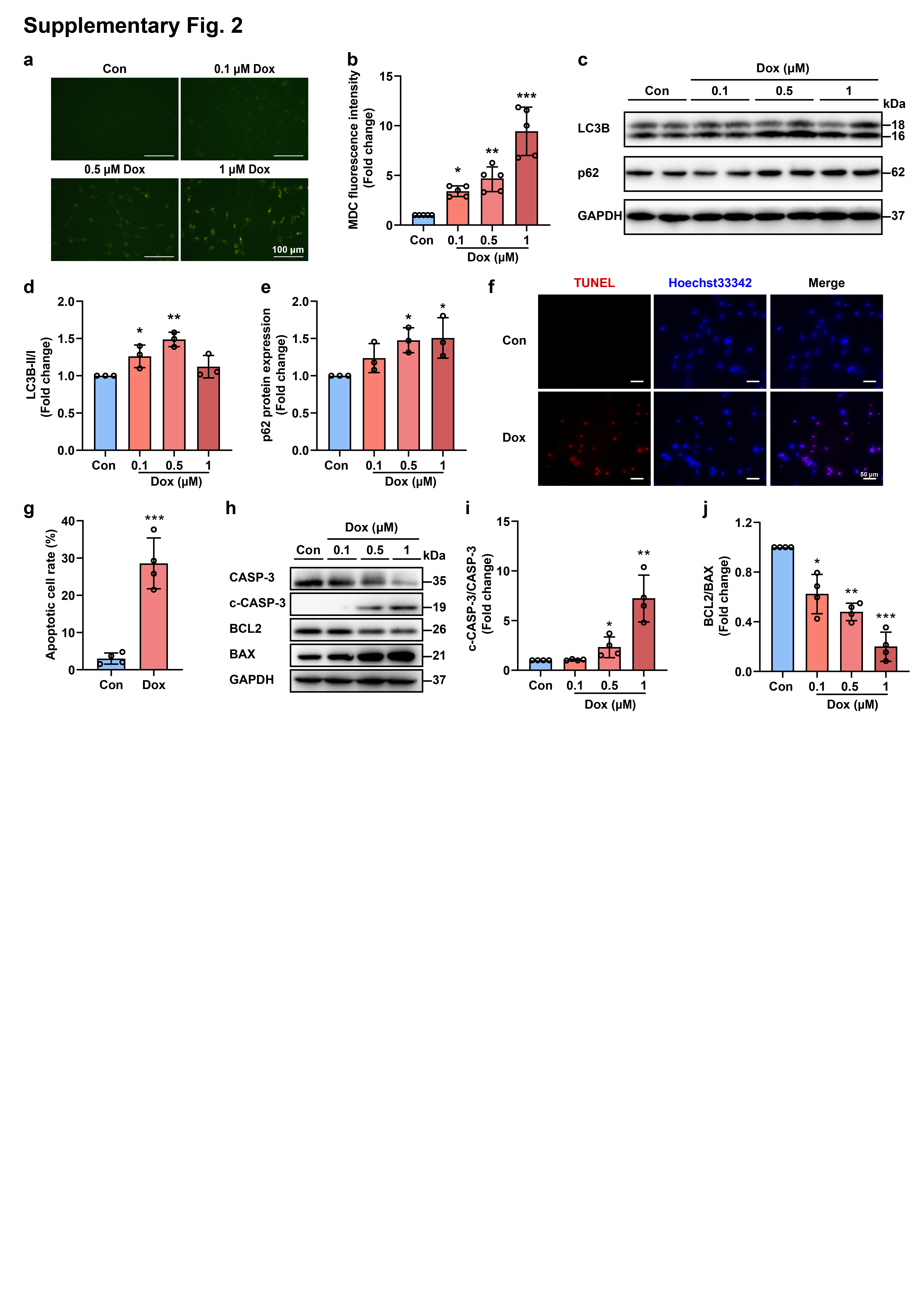


Supplementary Fig. 2. Dox mediates autophagic flux blockage and apoptosis in cardiomyocytes in vitro.

**a-b** Monodansylcadaverine (MDC) staining and the average data of cardiomyocytes treated with 0.1, 0.5, and 1 μM Dox for 24 hours; scale bar, 100 μm (n=5). **c-e** Western blot and the average data of the autophagy-related proteins LC3B and p62 in control (Con) and Dox-treated (Dox) cardiomyocytes (n=3). **f-g** Cell apoptosis and the average data of cardiomyocytes treated with 0.5 μM Dox for 24 hours and analyzed by TUNEL and Hoechst33342 staining; scale bar, 50 μm (n=4). **h-j** Western blot and the average data of the apoptosis-related proteins CASP-3, c-CASP-3, BCL2 and BAX in cardiomyocytes after treatment with 0.1, 0.5 and 1 μM Dox for 24 hours (n=4). * *P*< 0.05, ** *P*< 0.01, and *** *P*< 0.001.


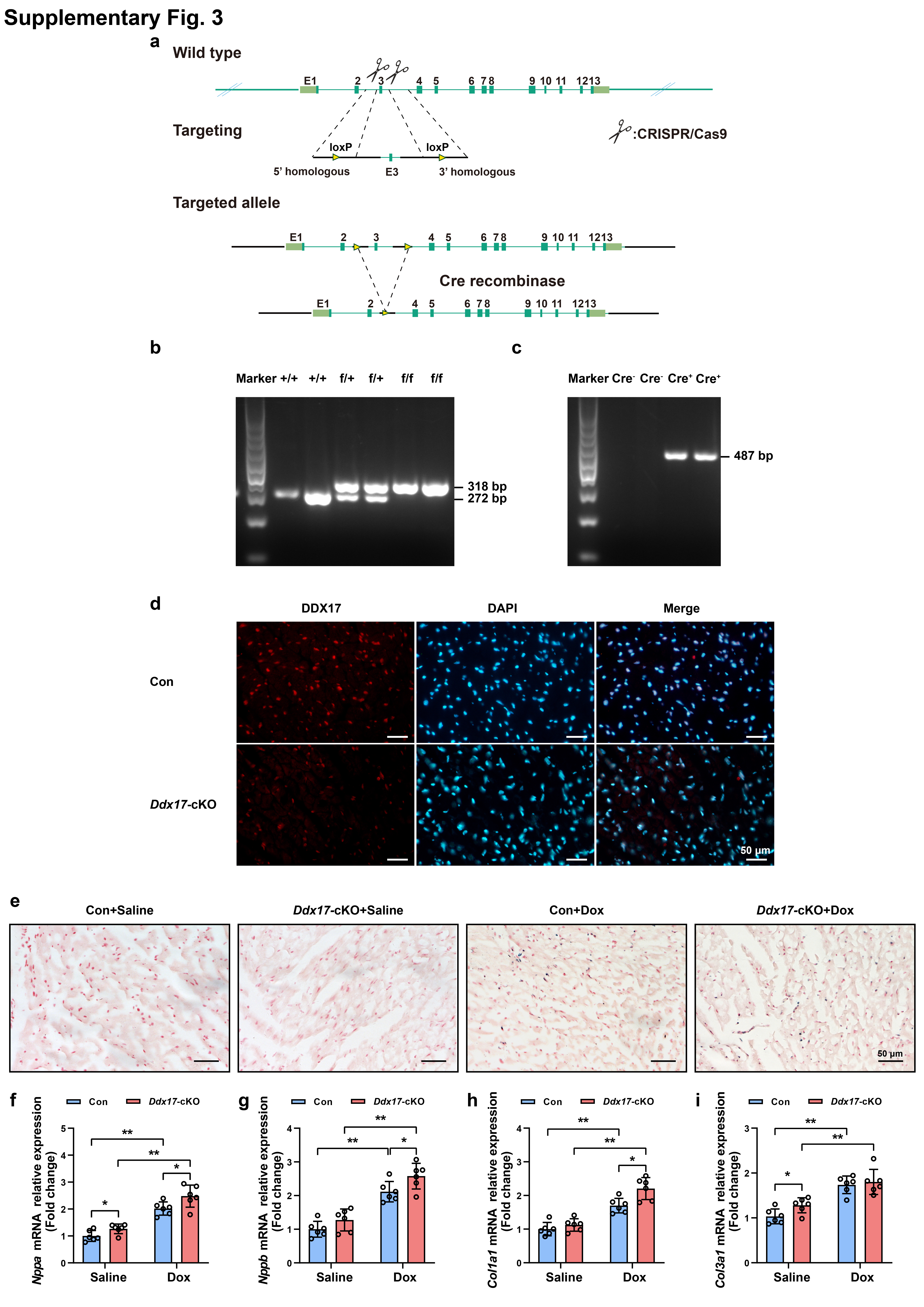


Supplementary Fig. 3. Cardiomyocyte-specific knockout of *Ddx17* in mice using the *loxP* and *α-MHC-Cre* systems and functional analysis.

**a** Schematic experimental outline of the generation of *Ddx17-*cKO mice. The flox mice were generated by separate insertion of two *loxP* sequences flanking the third exon of mouse *Ddx17* using CRISPR/Cas9 technology. Homozygous *Ddx17* flox mice were then crossed with *α-MHC-Cre* mice, resulting in the cardiomyocyte-specific knockout of *Ddx17.* **b** Representative images of genotyping results of wild-type (+/+), flox heterozygous (f/+) and homozygous (f/f) *Ddx17* flox mice. **c** Representative images of genotyping results of *α-MHC-Cre*^-^ and *α-MHC-Cre*^+^ mice. **d** Immunofluorescence staining for DDX17 (red) and DAPI (blue) in the frozen sections of heart tissue from control (Con) and *Ddx17-*cKO (cKO) mice (n=5); scale bar, 50 μm. **e** TUNEL staining of myocardial tissue from control+saline (Con+Saline), *Ddx17*-cKO+saline (*Ddx17*-cKO+Saline), control+doxorubicin (Con+Dox) and *Ddx17*-cKO+doxorubicin (*Ddx17*-cKO+Dox) groups (n=5); scale bar, 50 μm. **f-i** Expression of fetal genes *Nppa*, *Nppb*, and fibrosis genes *Col1a1* and *Col3a1* mRNA in the myocardium of the four groups of mice by RT real-time PCR (n=6). * *P*< 0.05 and ** *P*< 0.01.


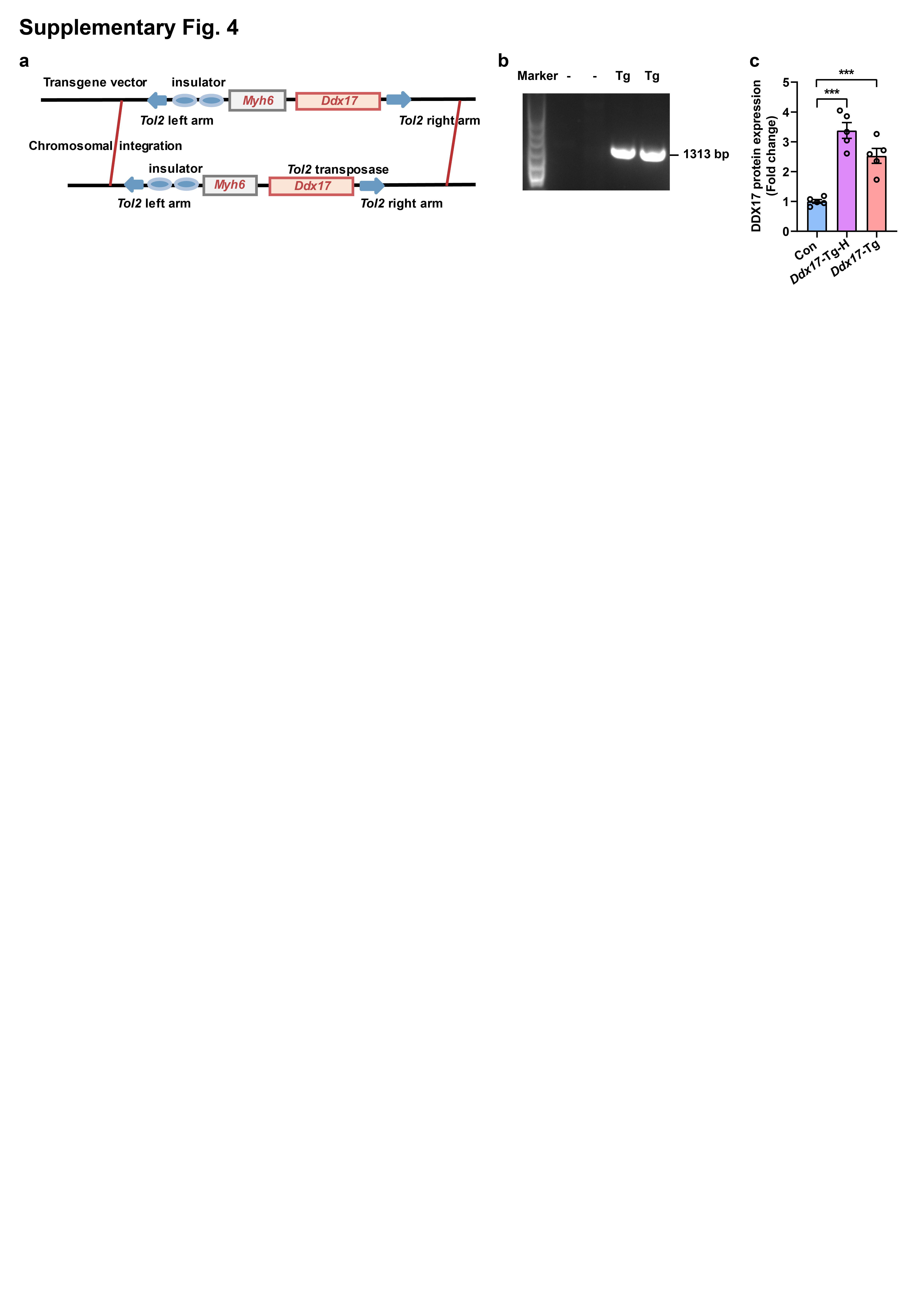


Supplementary Fig. 4. Construction and verification of cardiomyocyte-specific overexpression of *Ddx17* in mice and functional analysis.

**a** Schematic representation of the construction of cardiomyocyte-specific *Ddx17* transgenic mice. **b** Representative images of genotyping results of wild-type (-), and *Ddx17-*Tg (Tg) mice. **c** Average data of DDX17 protein expression from control (Con) and two cardiac-specific *Ddx17*-overexpressing mouse lines (*Ddx17*-Tg-H and *Ddx17*-Tg) (n=5). *** *P*< 0.001.


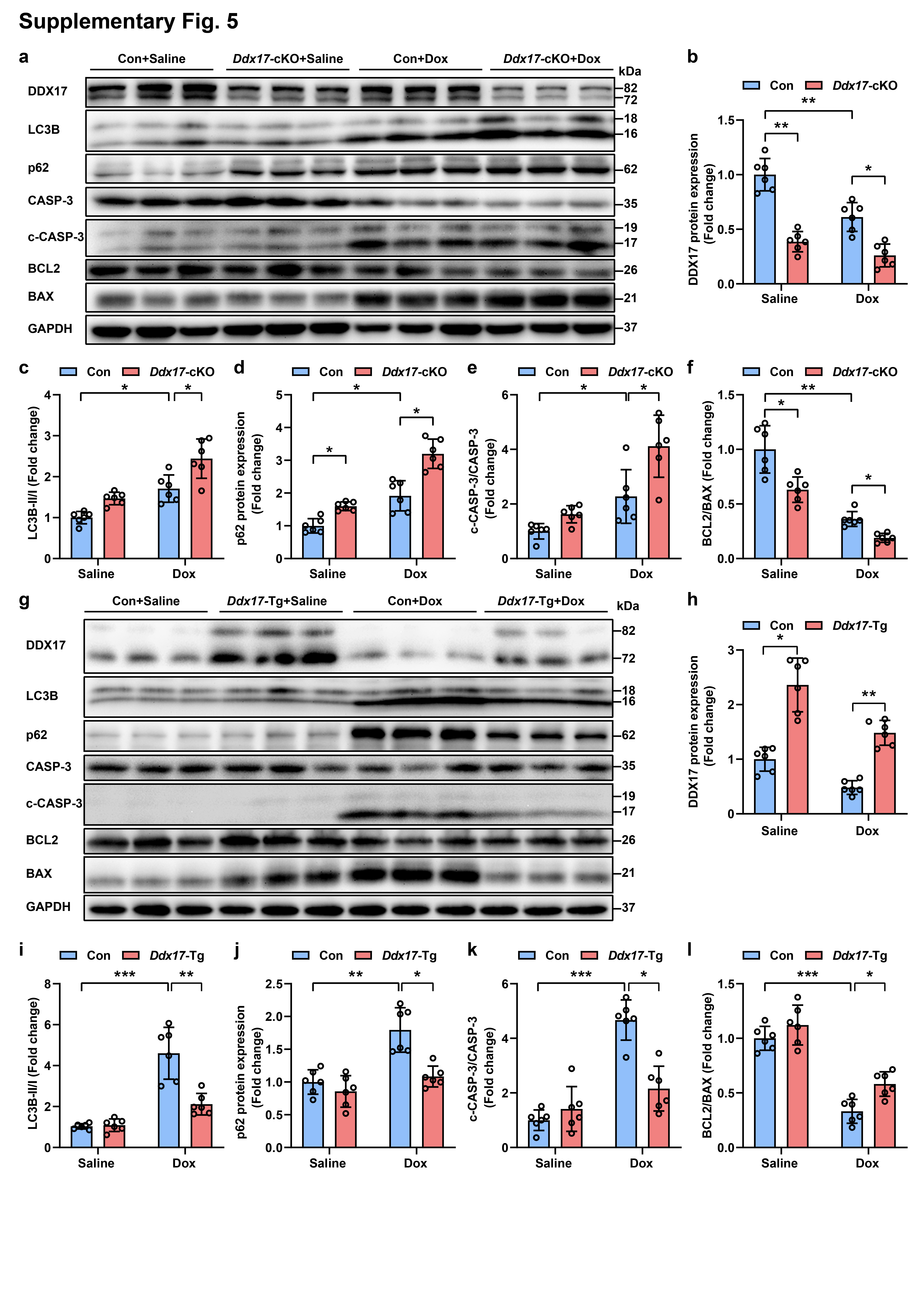


Supplementary Fig. 5. Cardiomyocyte-specific knockout and overexpression of *Ddx17* alter Dox-induced autophagic flux blockage and cell apoptosis.

**a-f** DDX17, LC3B, p62, CASP-3, c-CASP-3, BCL2 and BAX protein levels of myocardial tissue in Con+Saline, *Ddx17*-cKO+Saline, Con+Dox, and *Ddx17-*cKO+Dox groups (n=6). **g-l** Western blot of DDX17, LC3B, p62, CASP-3, c-CASP-3, BCL2 and BAX in myocardial tissue from the Con+Saline, *Ddx17*-Tg+Saline, Con+Dox and *Ddx17*-Tg+Dox groups (n=6). * *P* < 0.05, ** *P*< 0.01, and *** *P* < 0.001.


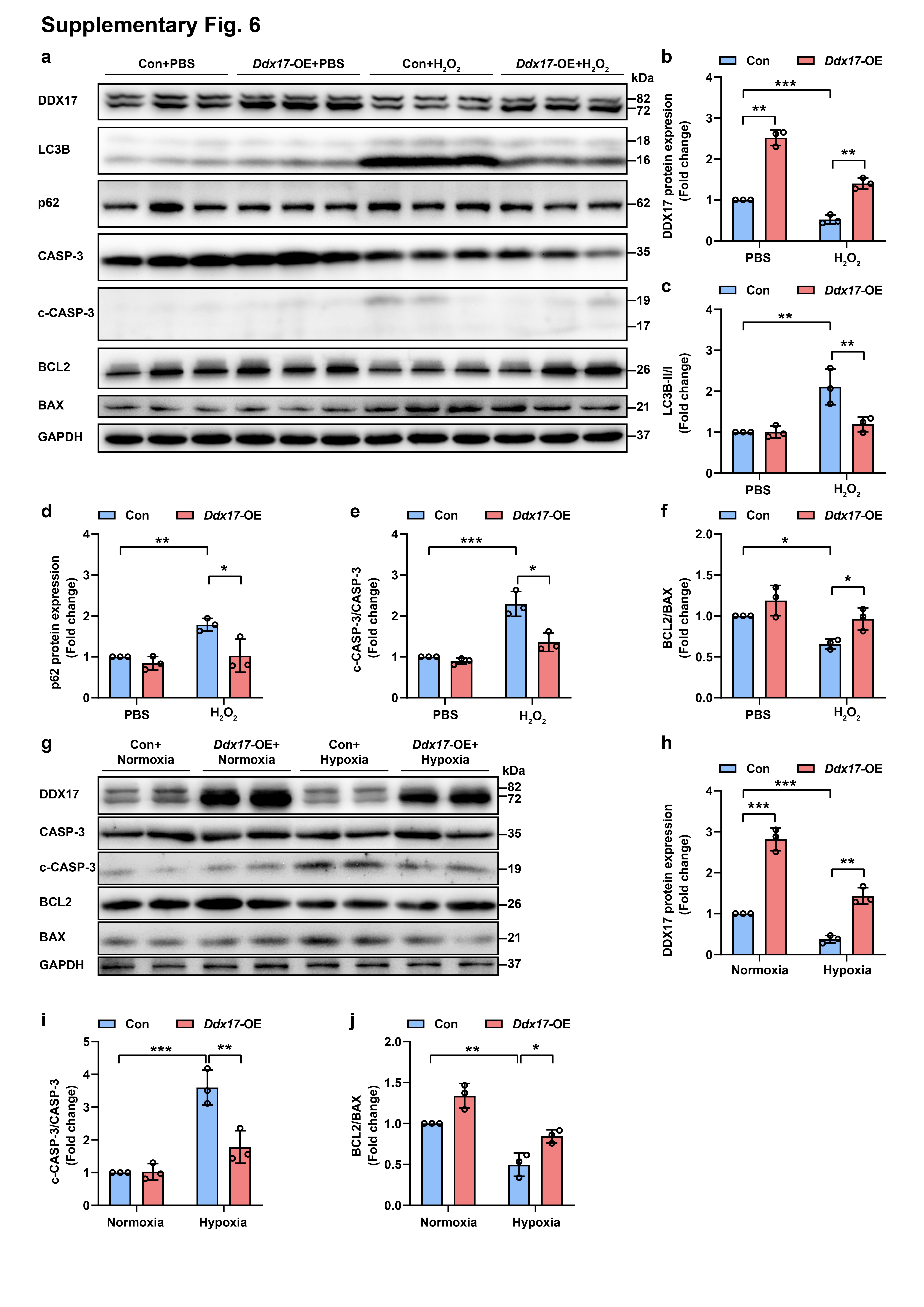


Supplementary Fig. 6. Overexpression of *Ddx17* (*Ddx17-*OE) in cardiomyocytes specifically inhibits H_2_O_2_- and hypoxia-induced autophagic flux blockage and the cell apoptosis.

**a-f** NMVMs were transfected with the empty vector adenovirus (Con) or *Ddx17*-overexpressing adenovirus (*Ddx17*-OE) at a multiplicity of infection (M.O.I.) of 30 for 24 hours and then treated with 100 μM H_2_O_2_ for 24 hours. The expression of autophagy- and apoptosis-related proteins in cardiomyocytes of the control adenovirus vector transfected+PBS (Con+PBS), Ad-*Ddx17*-transfected+PBS (*Ddx17-*OE+PBS), Con+H_2_O_2_, and *Ddx17-*OE+H_2_O_2_ groups was analyzed by western blot and statistical analysis (n=3). **g-j** Western blot and the average data of the expression of apoptosis-related proteins in the NMVMs of the Con+Normoxia, *Ddx17-*OE+Normoxia, Con+Hypoxia, and *Ddx17-*OE+Hypoxia groups (n=3). * *P*< 0.05, ** *P*< 0.01, and *** *P*< 0.001.


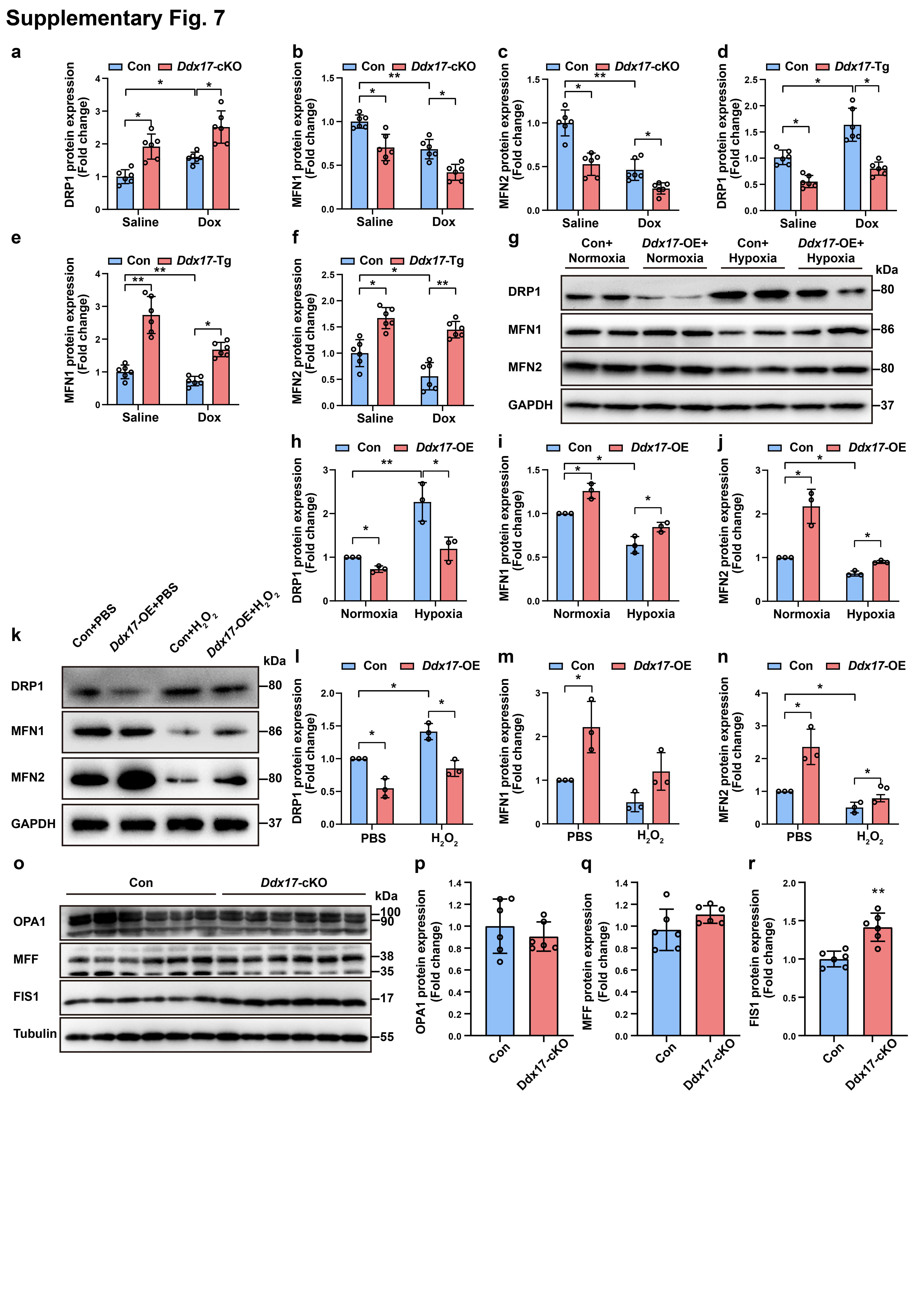


Supplementary Fig. 7. DDX17 regulates mitochondrial fission and mitochondrial homeostasis.

**a-c** Expression levels of DRP1, MFN1 and MFN2 in the left ventricle of the mice in the control+saline (Con+Saline), *Ddx17*-cKO+saline *(Ddx17*-cKO+Saline), control+doxorubicin (Con+Dox) and *Ddx17*-cKO+doxorubicin (*Ddx17*-cKO+Dox) groups by western blot (n=6). **d-f** Expression levels of DRP1, MFN1 and MFN2 in the left ventricle of mice in the control+saline (Con+Saline), *Ddx17*-transgene+saline (*Ddx17*-Tg+Saline), control+doxorubicin (Con+Dox) and *Ddx17*-transgene+doxorubicin (*Ddx17*-Tg+Dox) groups by western blot (n=6). **g-j** Western blot and the average data of DRP1, MFN1 and MFN2 in the control+normoxia (Con+Normoxia), *Ddx17*-overexpression+normoxia (*Ddx17-*OE+Normoxia), control+hypoxia (Con+Hypoxia), and *Ddx17*-overexpression+hypoxia (*Ddx17-*OE+Hypoxia) groups (n=3). **k-n** Western blot and the average data of DRP1, MFN1 and MFN2 in the control+PBS (Con+PBS), *Ddx17*-overexpression+PBS (*Ddx17-*OE+PBS), control+H_2_O_2_ (Con+H_2_O_2_), and *Ddx17*-overexpression+H_2_O_2_ (*Ddx17-*OE+H_2_O_2_) groups (n=3). **o-r** Western blot and the average data of OPA1, MFF and FIS1 in control (Con) and *Ddx17*-cKO mice (n=6). * *P*< 0.05 and ** *P*< 0.01.


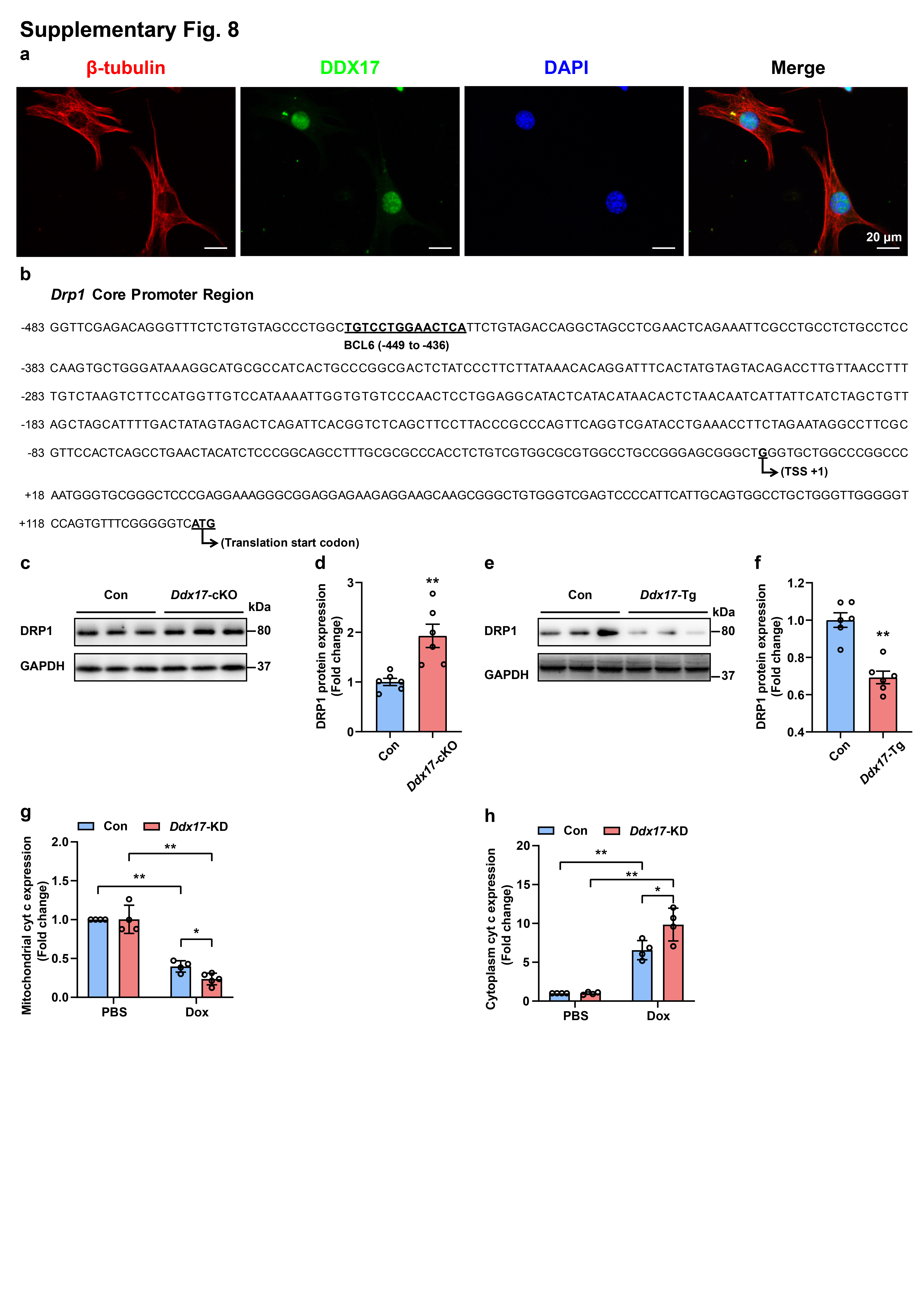


Supplementary Fig. 8. Expression of DRP1 in NMVMs of *Ddx17*-cKO and *Ddx17*-Tg mice.

**a** Immunofluorescence staining of β-tubulin (red), DDX17 (green), and DAPI (blue) in NMVMs (n=3); scale bar, 20 μm. **b** The core promoter sequence and the predicted binding site of BCL6 in the promoter region of *Drp1*. The transcription start site (TSS) is indicated by a backward arrow at the indicated position +1. The predicted binding sequence of the BCL6 transcription factor to *Drp1* is underlined in bold, along with the distance between the start and end nucleotides to the TSS. The position of the translation initiation codon (ATG) relative to the TSS is also indicated by a backward arrow. **c-d** Western blot and the average data of DRP1 in isolated NMVMs from control (Con) and *Ddx17*-cKO mice (n=6). **e-f** Western blot and average data of DRP1 expression in isolated NMVMs from control (Con) and *Ddx17*-Tg mice (n=6). **g-h** HL-1 cells were transfected with NCi (Con) and *Ddx17* siRNA (*Ddx17*-KD) for 24 hours, then treated with PBS or 0.5 μM Dox for 24 hours and divided into Con, *Ddx17*-KD, Con+Dox and *Ddx17*-KD+Dox groups. Cardiomyocyte mitochondria and cytoplasm were extracted separately using a mitochondrial isolation kit. The average data of cytochrome c levels in mitochondria and cytoplasm were analyzed by western blot. COX IV was used as a protein loading control for mitochondria and β-tubulin was used as a protein loading control for cytoplasmic proteins (n=4). * *P*< 0.05 and ** *P*< 0.01.

Supplementary Table 1. The sequence information of pGL3-*Drp1*-WT and pGL3-*Drp1*-MUT.

| **Plasmid** | **Sequence** |
| --- | --- |
| pGL3-*Drp1*-WT | ggtaccTCTCTTGGTGGGTAGGCTGGAGCGGTAGAAATGTCTGGAGACATTCTTTTAAGTTTTCCTATAGTTTCCTTGATTGCTCTTCATAAAGTAGTACTTCTCATAAGGAAATGACATTGGTTAATTAATAGACTCTATCCCTATTTTGGAAGGGGGGGGGGGTTCGAGACAGGGTTTCTCTGTGTAGCCCTGGCTGTCCTGGAACTCATTCTGTAGACCAGGCTAGCCTCGAACTCAGAAATTCGCCTGCCTCTGCCTCCCAAGTGCTGGGATAAAGGCATGCGCCATCACTGCCCGGCGACTaagctt |
| pGL3-*Drp1*-MUT | ggtaccTCTCTTGGTGGGTAGGCTGGAGCGGTAGAAATGTCTGGAGACATTCTTTTAAGTTTTCCTATAGGTTTCCTTGATTGCTCTTCATAAAGTAGTACTTCTCATAAGGAAATGACATTGGTTAATTAATAGACTCTATCCCTATTTTGGAAGGGGGGGGGGGTTCGAGACAGGGTTTCTCTGTGTAGCCCTGGCCAGTTGAAGGAGTGTTCTGTAGACCAGGCTAGCCTCGAACTCAGAAATTCGCCTGCCTCTGCCTCCCAAGTGCTGGGATAAAGGCATGCGCCATCACTGCCCGGCGACTaagctt |

Predicted BCL6 binding sites or mutated binding sites in the promoter region of the *Drp1* gene are underlined. The inserted sequence contains a 5' *Kpn* I restriction endonuclease cleavage site and a 3' *Hind* III restriction endonuclease cleavage site.

Supplementary Table 2. The basic clinical date of the patients of myocardial biopsy.

| **Gender** | **Age** | **EF (%)** | **BNP (pg/mL)** | **Diagnose** |
| --- | --- | --- | --- | --- |
| Male | 34 | 20 | 1042.76 | Coronary heart disease |
| Male | 31 | 25 | 1067.33 | Dilated cardiomyopathy |
| Male | 57 | 28 | 237.1 | Hypertensive heart disease |
| Male | 30 | 32 | 354.84 | Hypertensive heart disease |
| Male | 61 | 33 | 677.42 | Coronary heart disease |
| Male | 29 | 40 | 521.44 | Coronary heart disease |
| Male | 31 | 40 | 341.78 | Coronary heart disease |
| Male | 50 | 45 | 300.19 | Coronary heart disease |
| Male | 41 | 54 | 191.36 | Dilated cardiomyopathy |
| Male | 52 | 55 | 306.32 | Hypertensive heart disease |
| Male | 64 | 58 | 158.27 | Coronary heart disease |
| Male | 34 | 60 | 404.83 | Hypertensive heart disease |
| Male | 58 | 60 | 287 | Hypertensive heart disease |
| Male | 63 | 65 | 52.72 | Hypertensive heart disease |
| Male | 41 | 68 | 396.07 | Hypertensive heart disease |

Supplementary Table 3. Antibody information.

| **Antibodies** | **Manufacturer** | **Cat. No.** | **Source** | **Application(s) and Dilution** |
| --- | --- | --- | --- | --- |
| BAX | Cell Signaling Technology | 2772 | rabbit | WB (1:1000) |
| BCL2 | Cell Signaling Technology | 3498 | rabbit | WB (1:1000) |
| BCL6 | Santa Cruz | sc-7388 | mouse | WB (1:500),  Co-IP (2 µg per 500 µg of total protein),  IF (1:100) |
| BCL6 | Cell Signaling Technology | 5650 | rabbit | WB (1:1000),  ChIP (1:200) |
| BCL6 | Proteintech | 66340-1-Ig | mouse | WB (1:500) |
| Caspase-3 | Cell Signaling Technology | 9662 | rabbit | WB (1:1000) |
| Caspase-3 | Cell Signaling Technology | 14220 | rabbit | WB (1:1000) |
| cleaved-Caspase-3 | Cell Signaling Technology | 9661 | rabbit | WB (1:1000) |
| COX IV | Cell Signaling Technology | 4844 | rabbit | WB (1:1000) |
| cytochrome c | Cell Signaling Technology | 4272 | rabbit | WB (1:1000) |
| DDX17 | Abcam | Ab180190 | rabbit | WB (1:1000),  IF (1:200) |
| DDX17 | Santa Cruz | sc-398168 | mouse | WB (1:500),  Co-IP (2 µg per 500 µg of total protein) |
| DDX17 | Proteintech | 19910-1-AP | rabbit | WB (1:1000),  IF (1:200) |
| DRP1 | Proteintech | 12957-1-AP | rabbit | WB (1:500) |
| DRP1 | Cell Signaling Technology | 8570 | rabbit | WB (1:1000) |
| FIS1 | Proteintech | 10956-1-AP | rabbit | WB (1:500) |
| GAPDH | Cell Signaling Technology | 2118 | rabbit | WB (1:1000) |
| LC3B | Sigma-Aldrich | SAB5701328 | rabbit | WB (1:1000) |
| MFF | Proteintech | 17090-1-AP | rabbit | WB (1:3000) |
| MFN1 | Proteintech | 13798-1-AP | rabbit | WB (1:1000) |
| MFN2 | Proteintech | 12186-1-AP | rabbit | WB (1:1000) |
| OPA1 | Proteintech | 27733-1-AP | rabbit | WB (1:500) |
| p62 | Cell Signaling Technology | 5114 | rabbit | WB (1:1000) |
| β-tubulin | Proteintech | 10094-1-AP | rabbit | WB (1:1000),  IF (1:400) |
| Anti-rabbit IgG | Cell Signaling Technology | 7074 | goat | WB (1:1000) |
| Anti-mouse IgG | Cell Signaling Technology | 7076 | horse | WB (1:1000) |
| Anti-mouse IgG (H+L), (Alexa Fluor® 488) | Cell Signaling Technology | 4408 | goat | IF (1:400) |
| Anti-rabbit IgG (H+L), (Alexa Fluor® 594) | Cell Signaling Technology | 8889 | goat | IF (1:400) |
