# Supplementary material for: DEAD-box helicase 17 (DDX17) protects cardiac function by promoting mitochondrial homeostasis in heart failure
Source: Signal Transduct Target Ther. 2024 May 24;9:127. doi: 10.1038/s41392-024-01831-2 (PMC11116421; doi:10.1038/s41392-024-01831-2)

## Figure 1d

DDX17 (82 and 72 kDa)

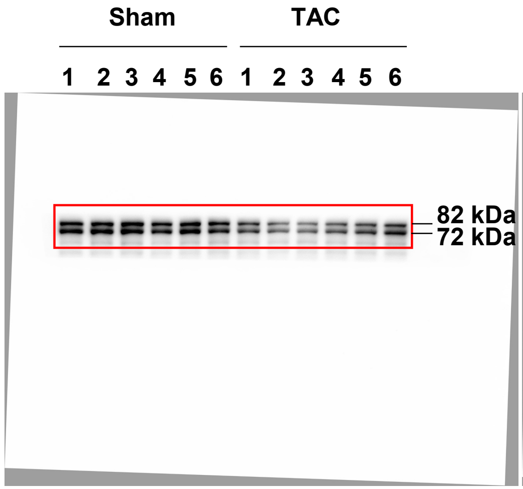


GAPDH (37 kDa)

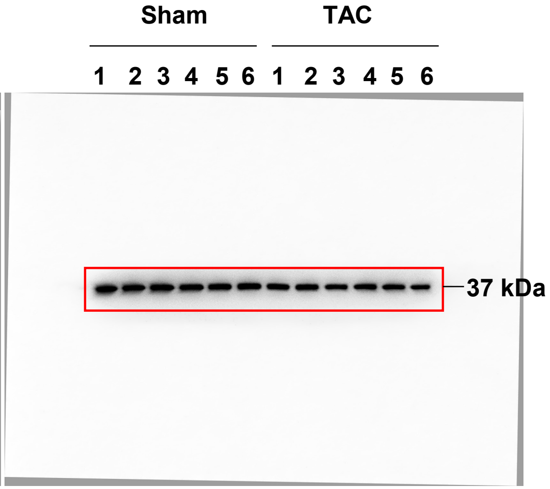


## Figure 1k

DDX17 (82 and 72 kDa)

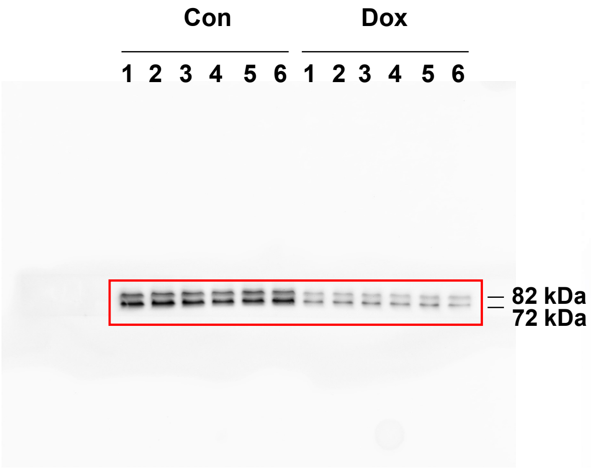


GAPDH (37 kDa)

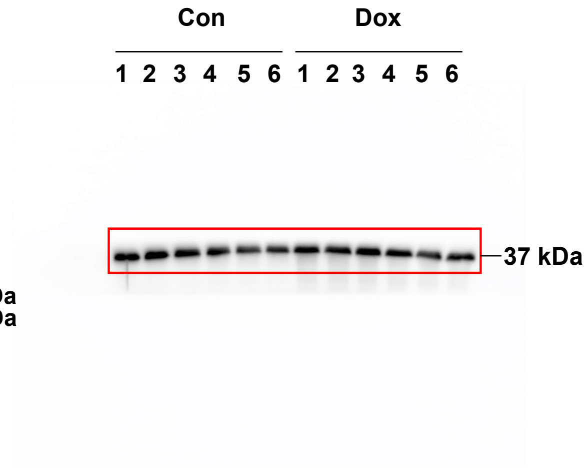


## Figure 1m

DDX17 (82 and 72 kDa)

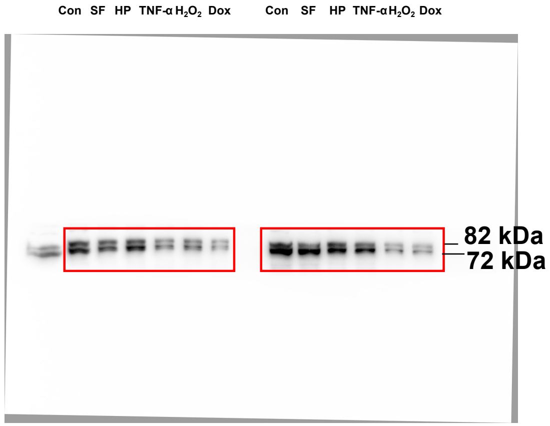


GAPDH (37 kDa)

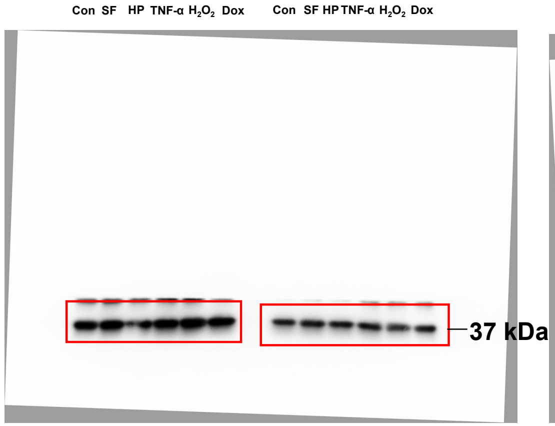


## Figure 1m

DDX17 (82 and 72 kDa)

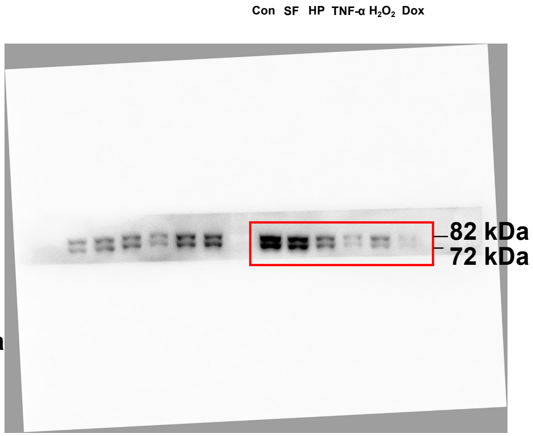


GAPDH (37 kDa)

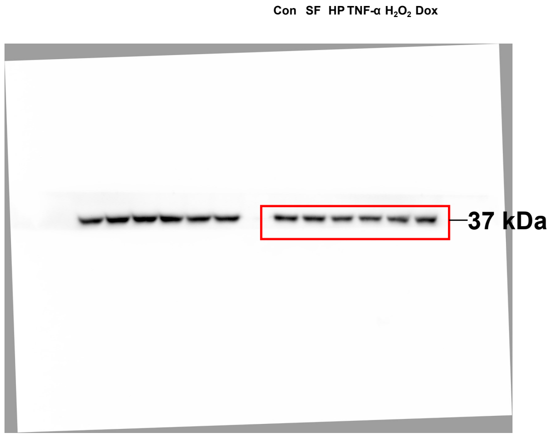

Figure 1o

DDX17 (82 and 72 kDa)

Normoxia Hypoxia Normoxia Hypoxia

1 2 3 1 2 3 1 2 3 1 2 3

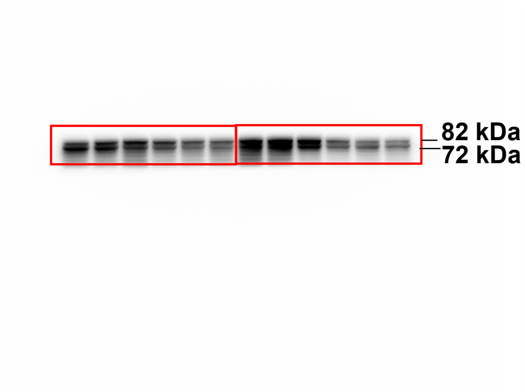


GAPDH (37 kDa)

Normoxia Hypoxia Normoxia Hypoxia

1 2 3 1 2 3 1 2 3 1 2 3

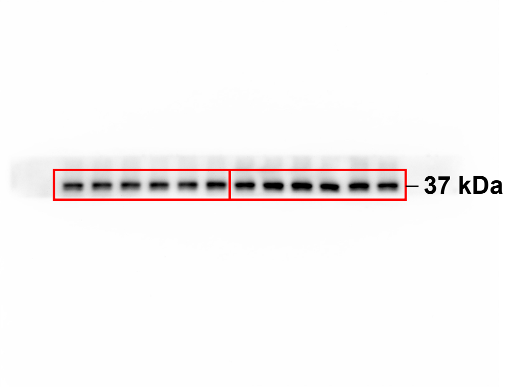


DDX17 (82 and 72 kDa)

Normoxia Hypoxia Normoxia Hypoxia

1 2 3 1 2 3 1 2 3 1 2 3

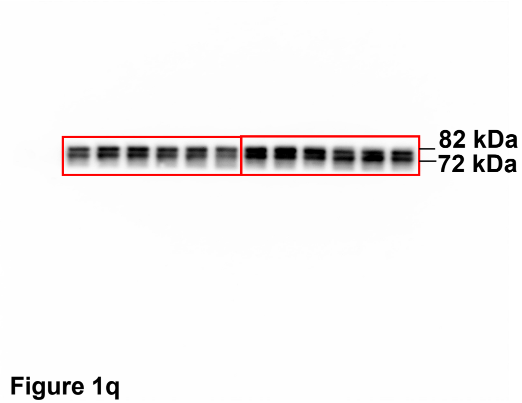


GAPDH (37 kDa)

Normoxia Hypoxia Normoxia Hypoxia

1 2 3 1 2 3 1 2 3 1 2 3

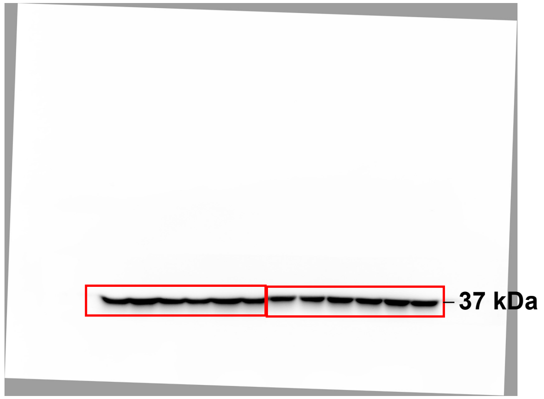


Normoxia Hypoxia

1 2 3 4 5 1 2 3 4 5 6

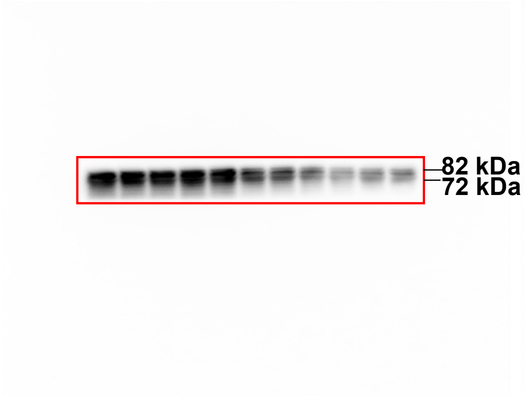


Normoxia Hypoxia

1 2 3 4 5 1 2 3 4 5 6

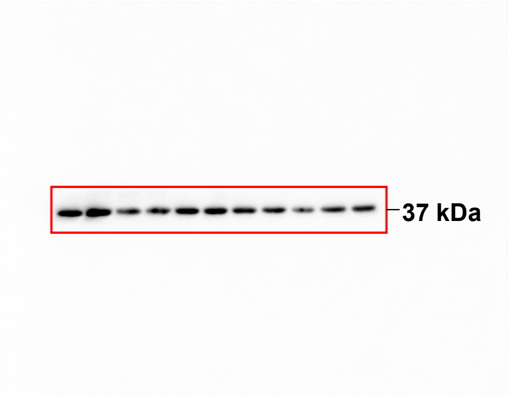


Figure 1q

DDX17 (82 and 72 kDa)

$H_2O_2$ (μM)

Con 50 100 200 300

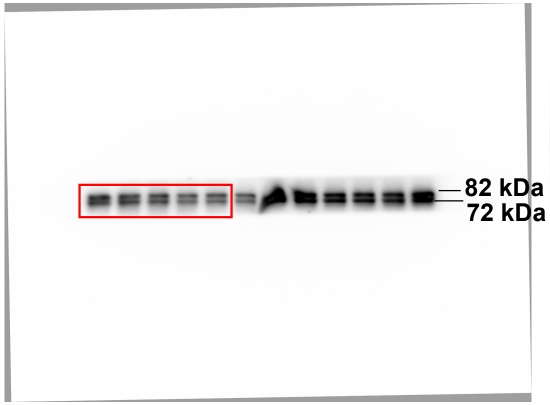


GAPDH (37 kDa)

$H_2O_2$ (μM)

Con 50 100 200 300

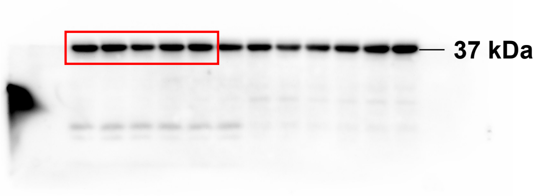

Figure 1q

DDX17 (82 and 72 kDa)

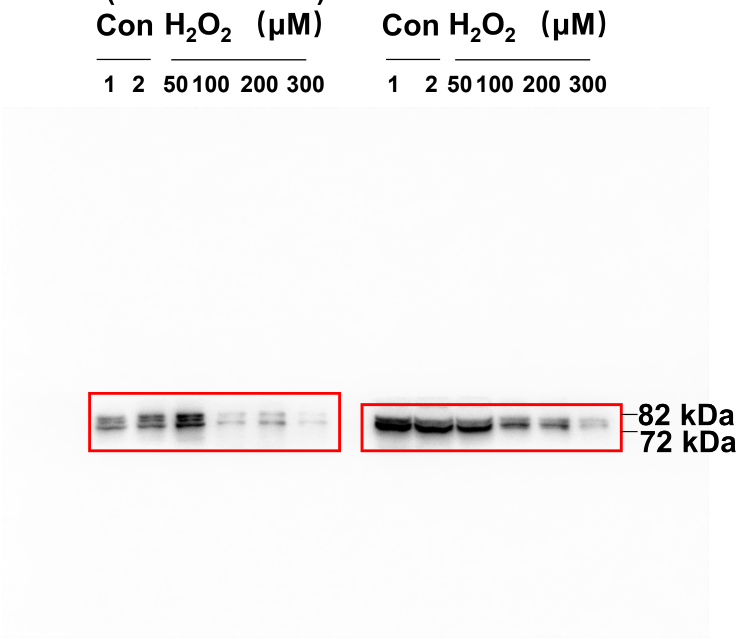


GAPDH (37 kDa)

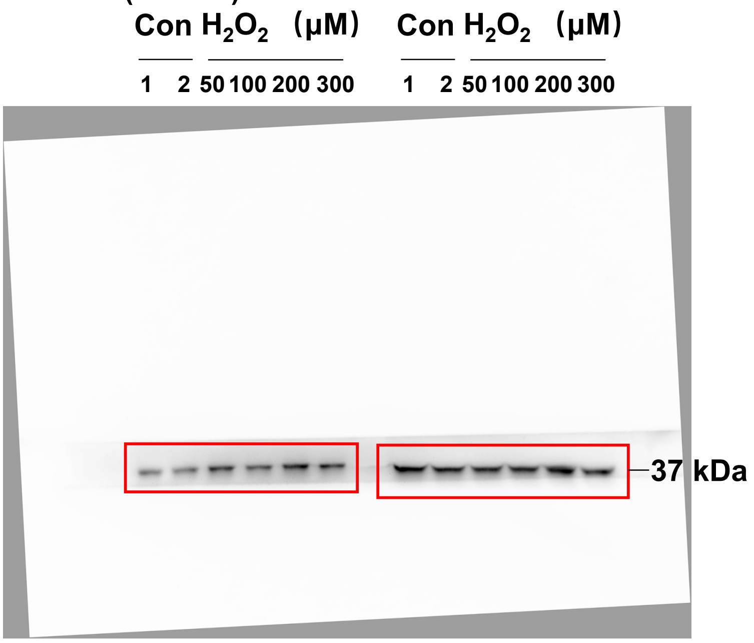


Figure 1s

DDX17 (82 and 72 kDa)

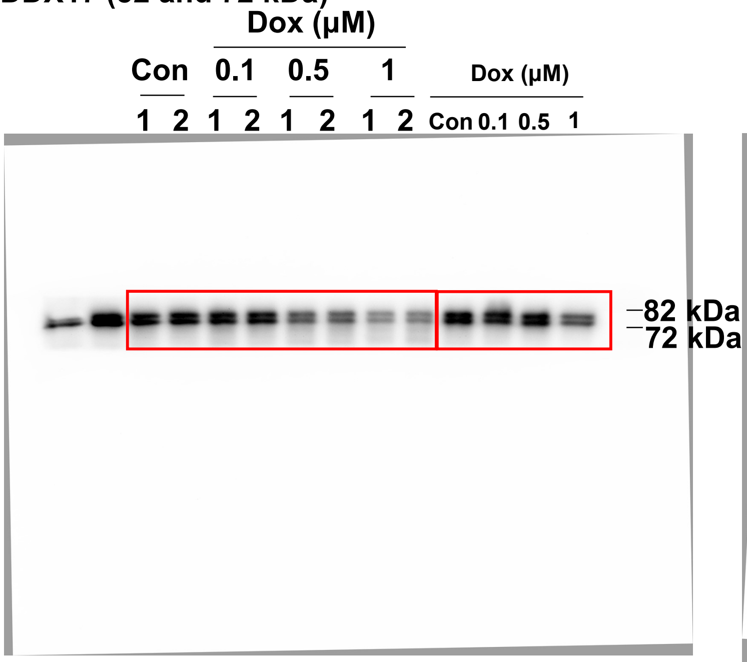


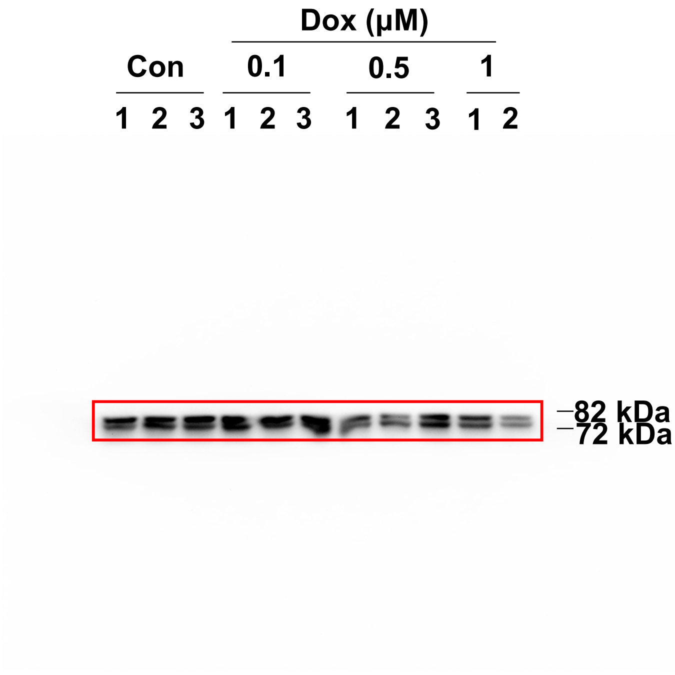


GAPDH (37 kDa)

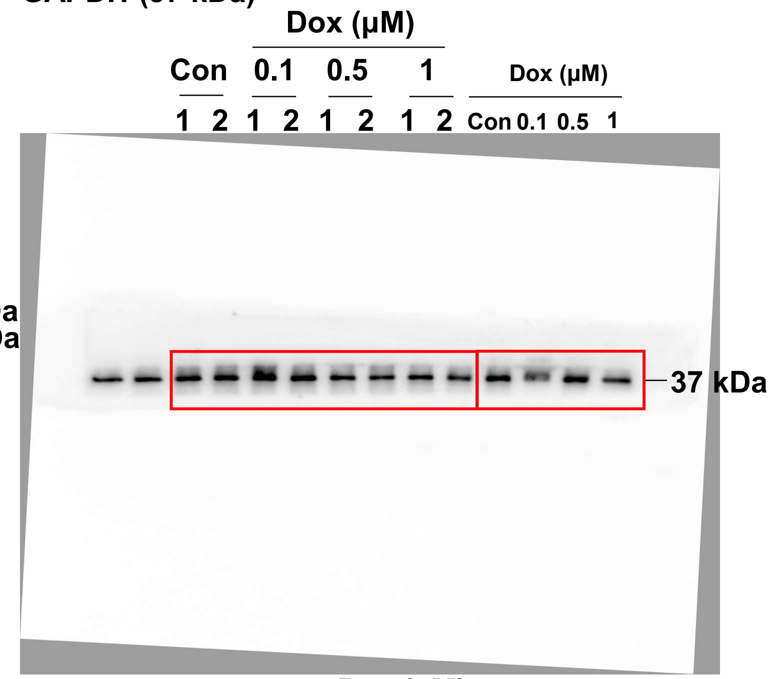


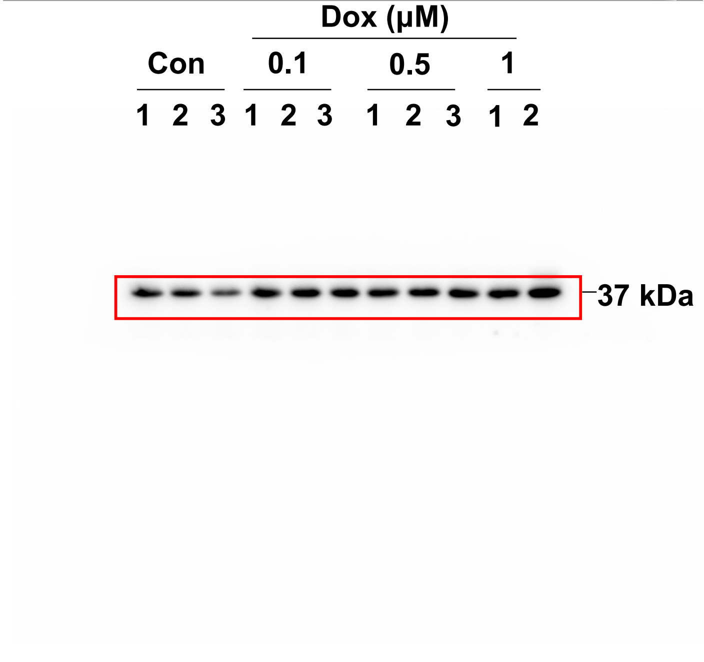

## Figure 2a

DDX17 (82 and 72 kDa)

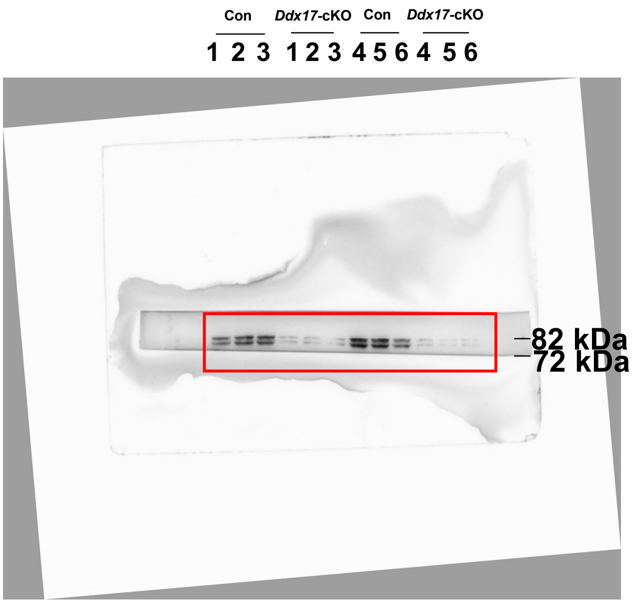


GAPDH (37 kDa)

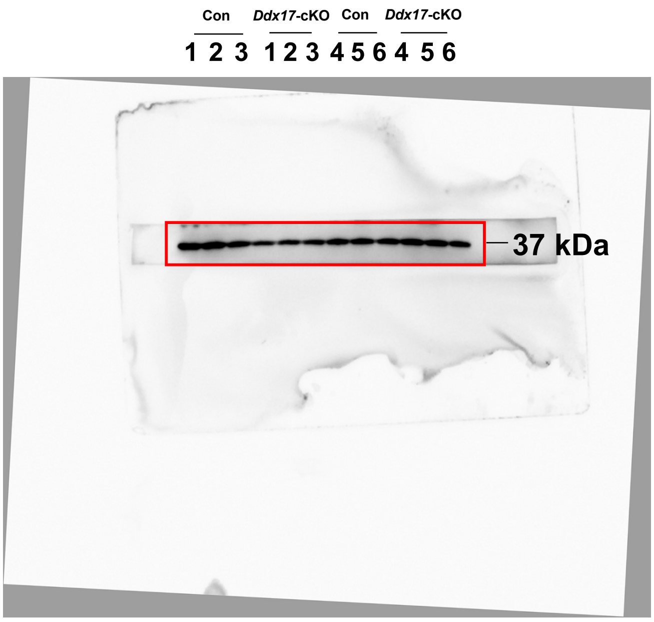


## Figure 2c

DDX17 (82 and 72 kDa)

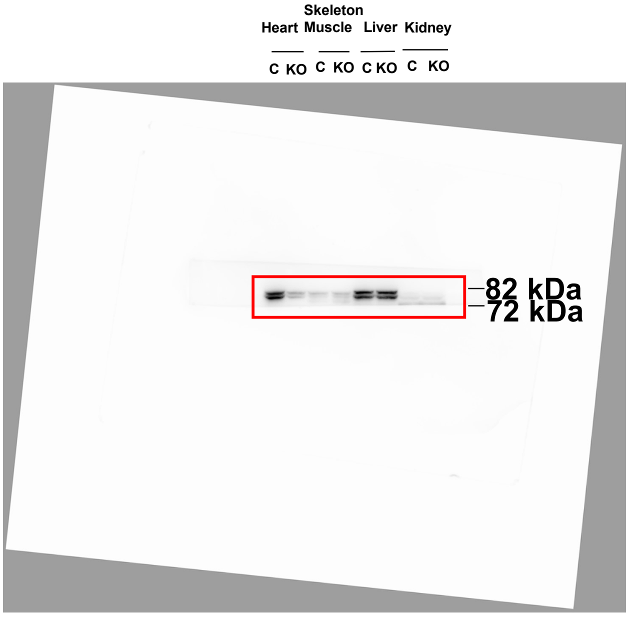


GAPDH (37 kDa)

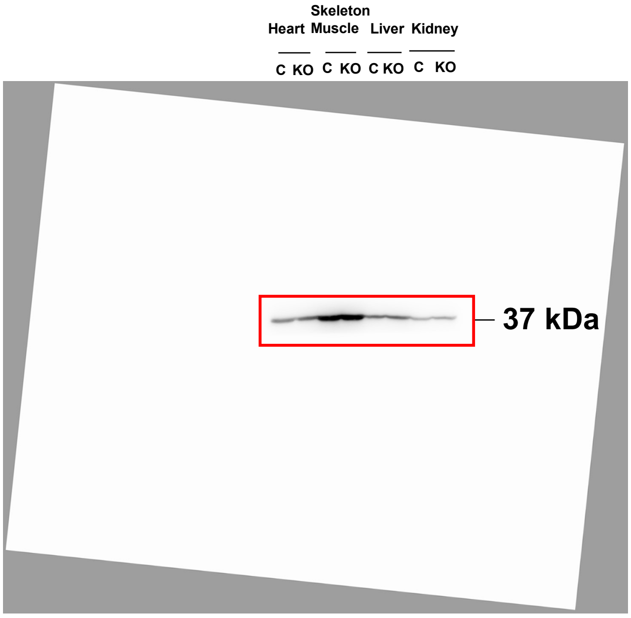


## Figure 3a

DDX17 (82 and 72 kDa)

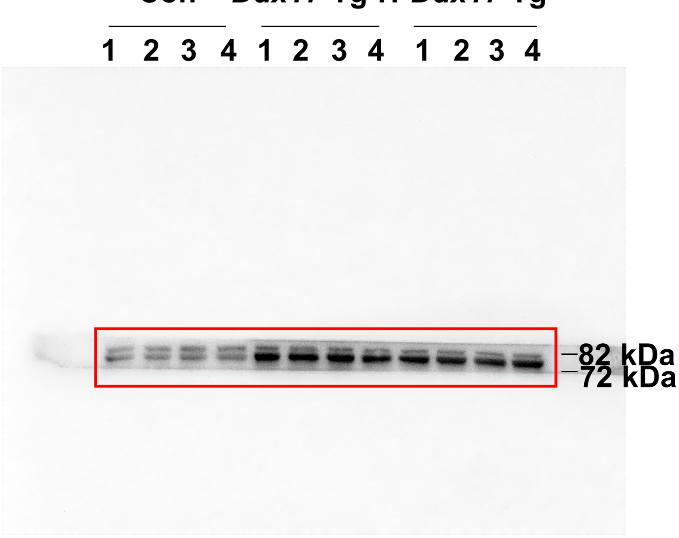


GAPDH (37 kDa)

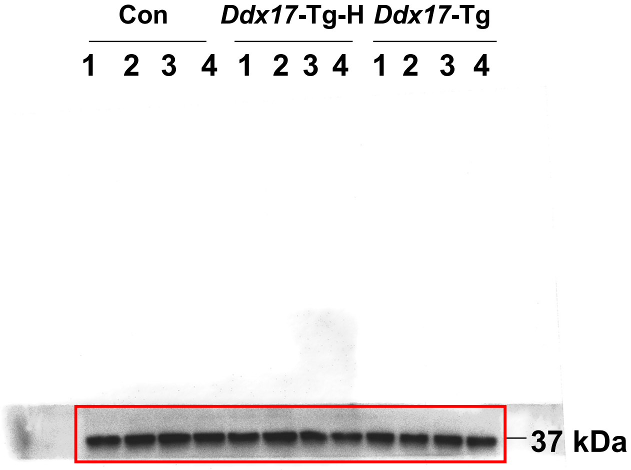

## Figure 3a

DDX17 (82 and 72 kDa)

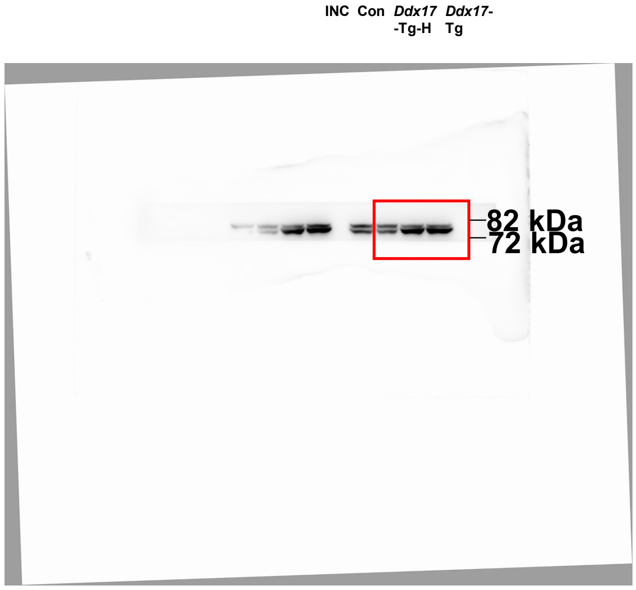


GAPDH (37 kDa)

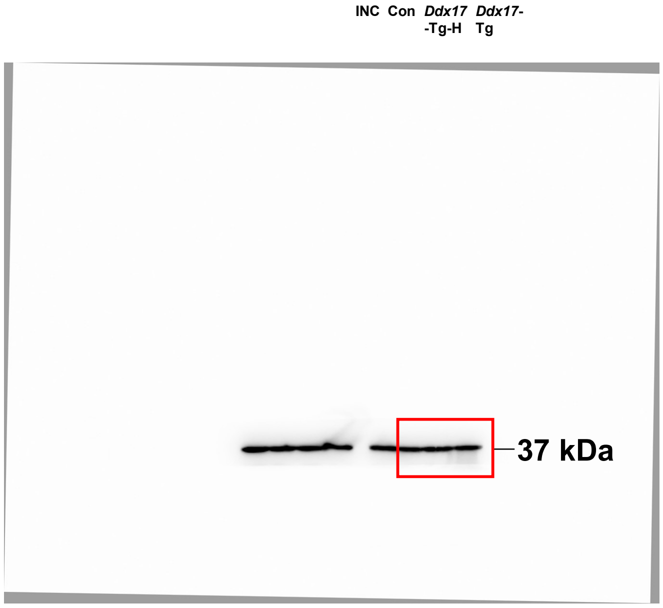


## Figure 3b

DDX17 (82 and 72 kDa)

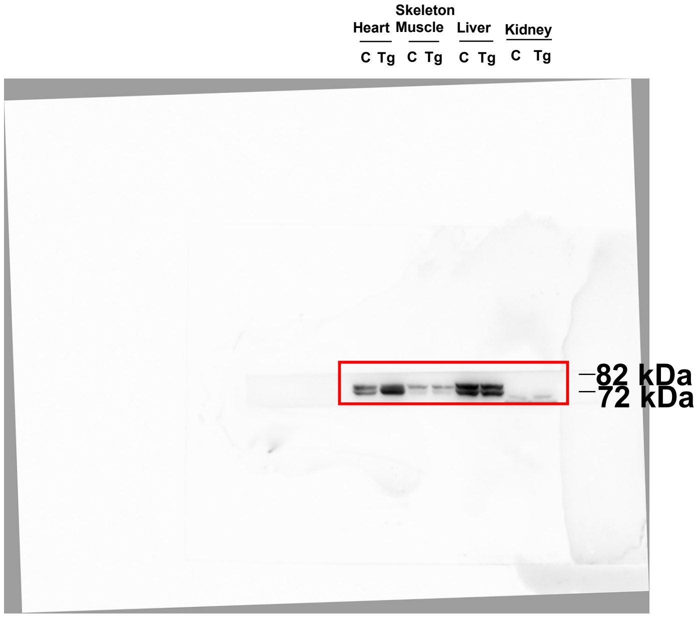


GAPDH (37 kDa)

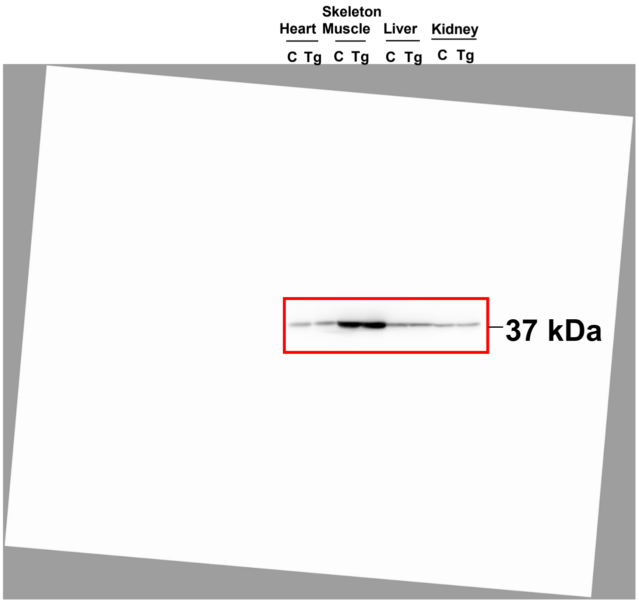


## Figure 4s

DRP1 (80 kDa)

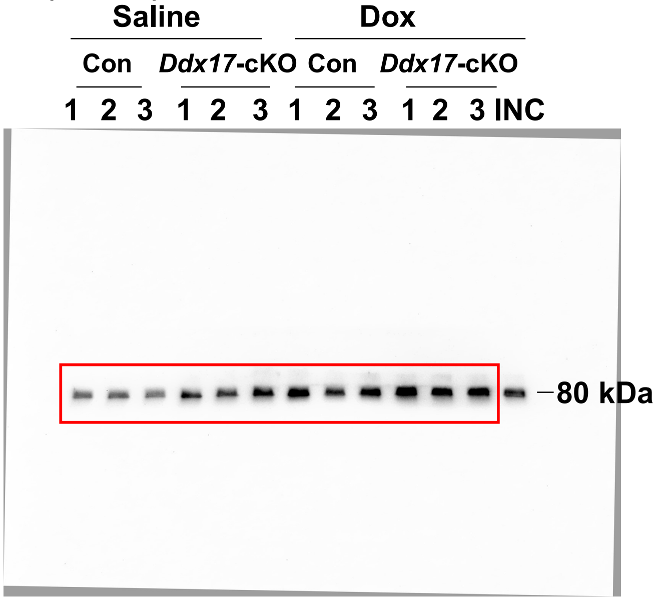


MFN1 (86 kDa)

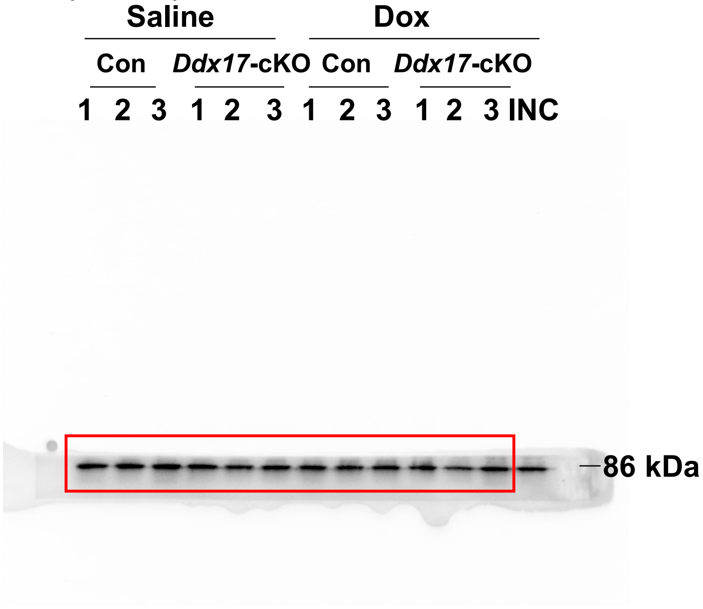


INC: internal positive control

Figure 4s

DRP1 (80 kDa)

Saline | Dox
Con | *Ddx17*-cKO | Con | *Ddx17*-cKO
4 5 6 4 5 6 4 5 6 4 5 6 INC

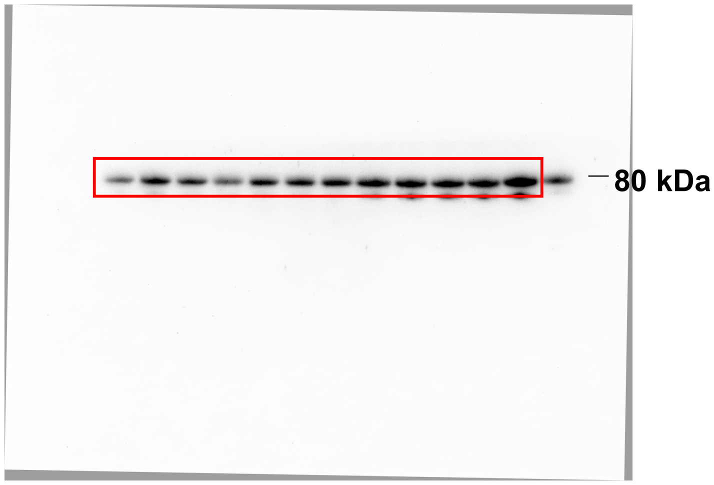


MFN1 (86 kDa)

Saline | Dox
Con | *Ddx17*-cKO | Con | *Ddx17*-cKO
4 5 6 4 5 6 4 5 6 4 5 6 INC

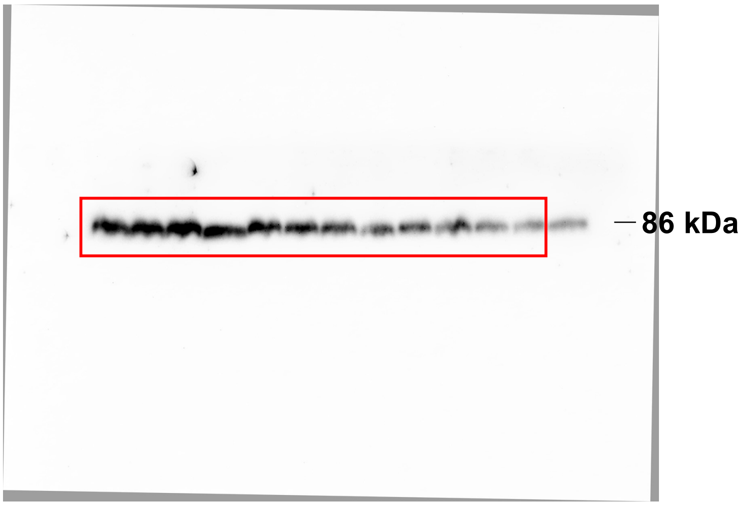


MFN2 (80 kDa)

Saline | Dox
Con | *Ddx17*-cKO | Con | *Ddx17*-cKO
1 2 3 1 2 3 1 2 3 1 2 3 INC

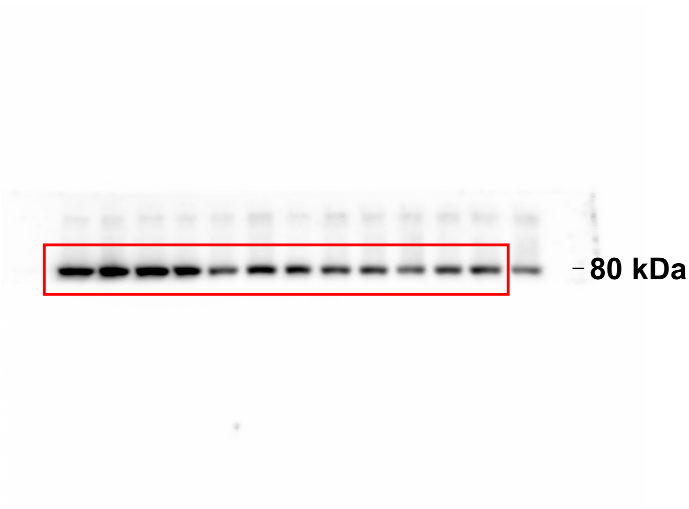


Saline | Dox
Con | *Ddx17*-cKO | Con | *Ddx17*-cKO
4 5 6 4 5 6 4 5 6 4 5 6 INC

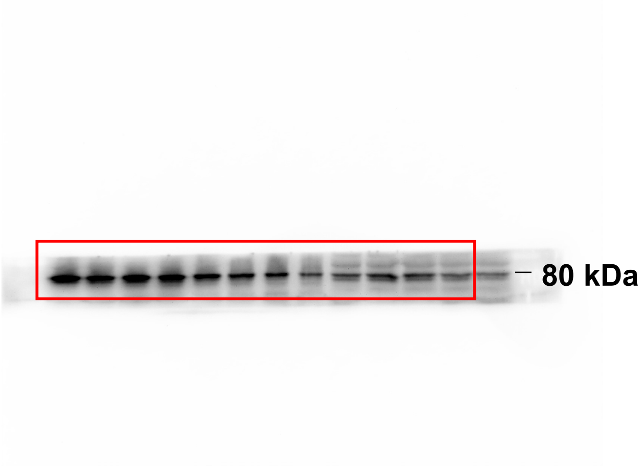


GAPDH (37 kDa)

Saline | Dox
Con | *Ddx17*-cKO | Con | *Ddx17*-cKO
1 2 3 1 2 3 1 2 3 1 2 3 INC

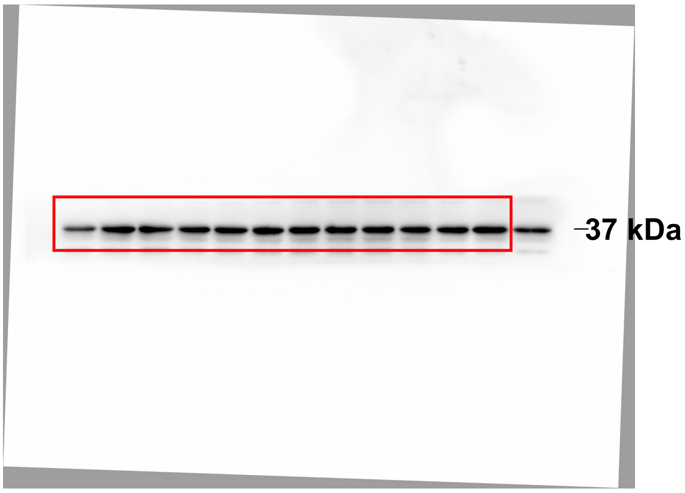


Saline | Dox
Con | *Ddx17*-cKO | Con | *Ddx17*-cKO
4 5 6 4 5 6 4 5 6 4 5 6 INC

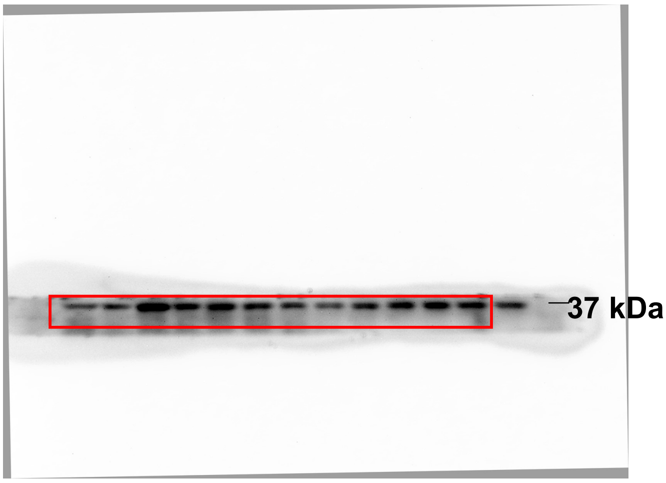

**Figure 4s**

**GAPDH (37 kDa)**

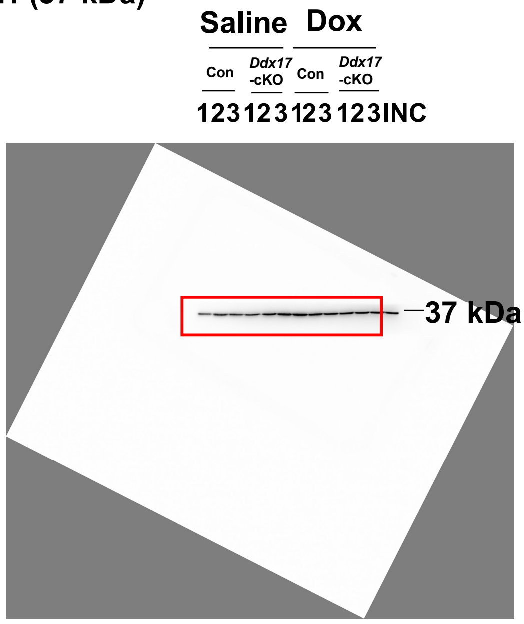


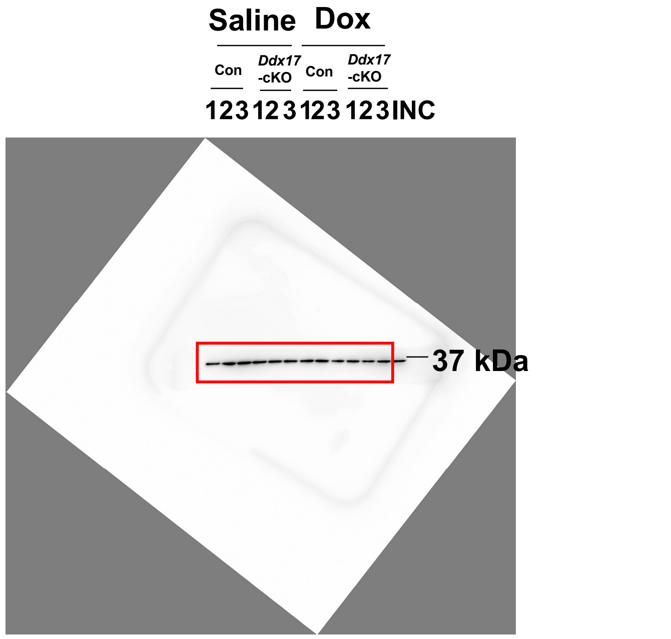


**Figure 4t**

**DRP1 (80 kDa)**

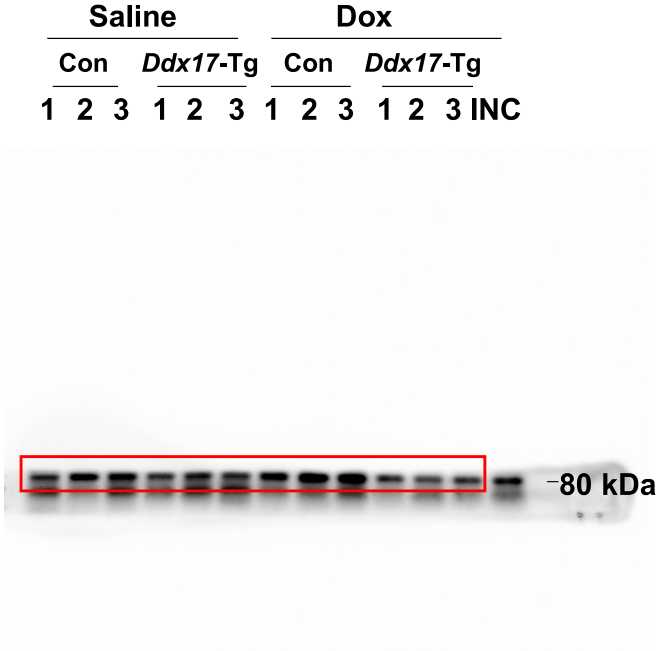


**MFN1 (86 kDa)**

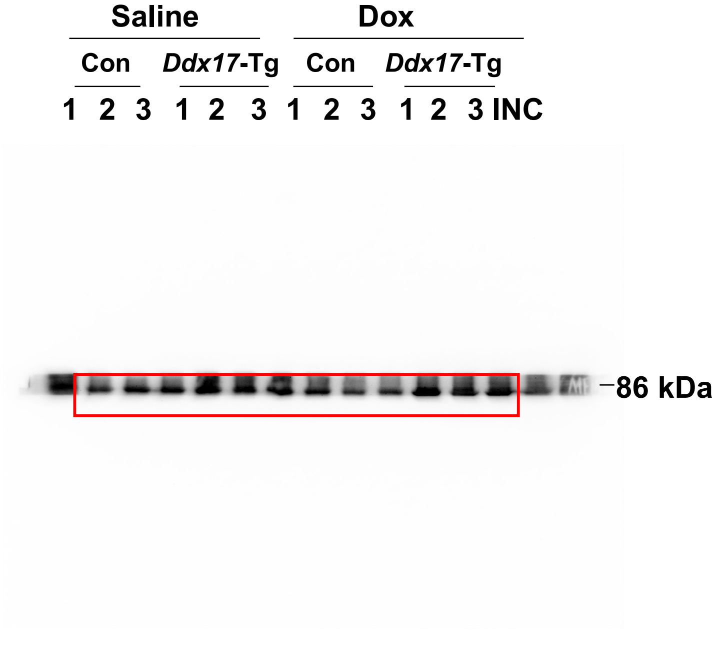


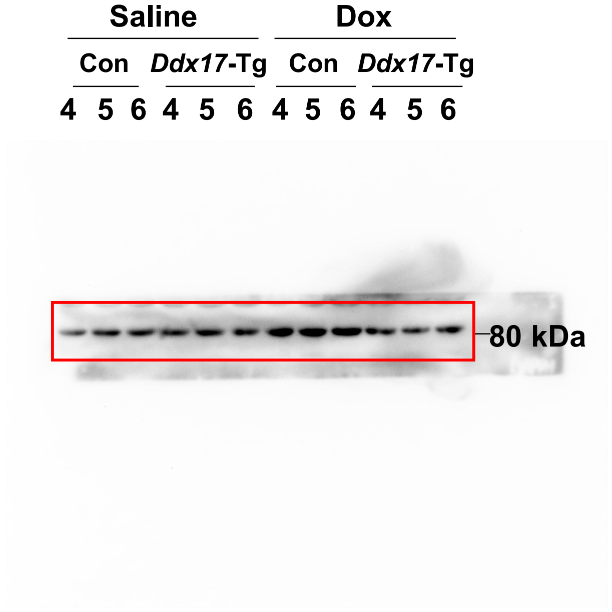


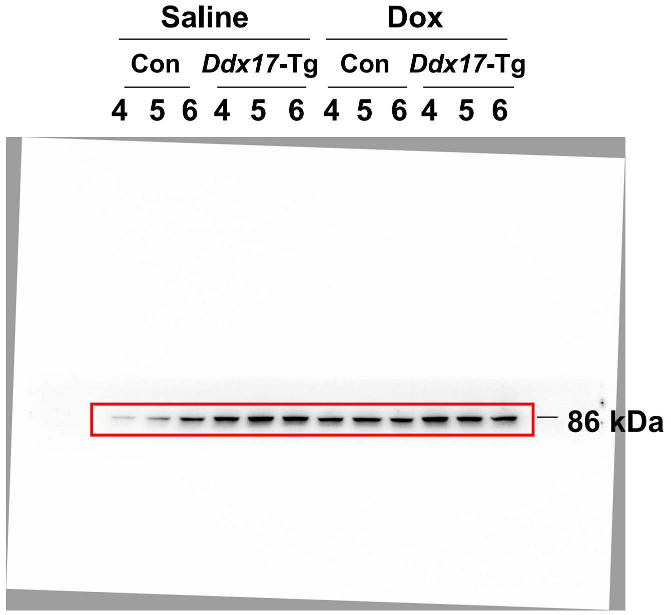

Figure 4t

MFN2 (80 kDa)

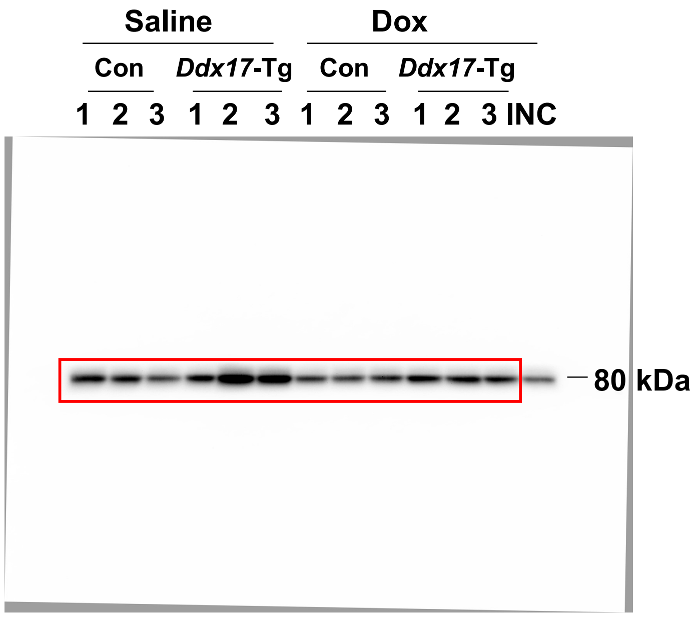


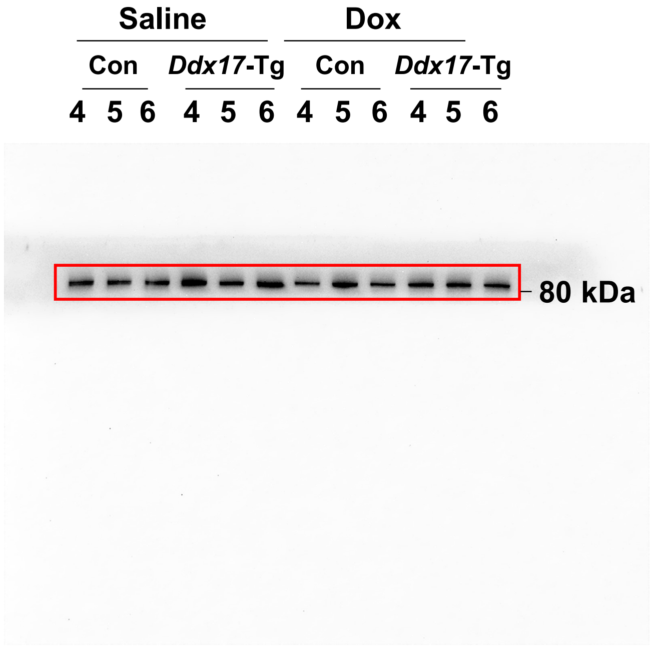


GAPDH (37 kDa)

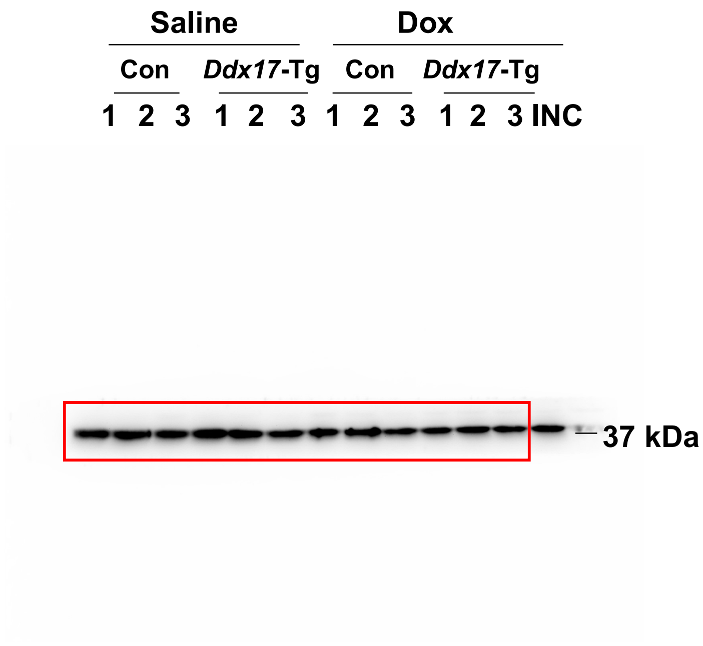


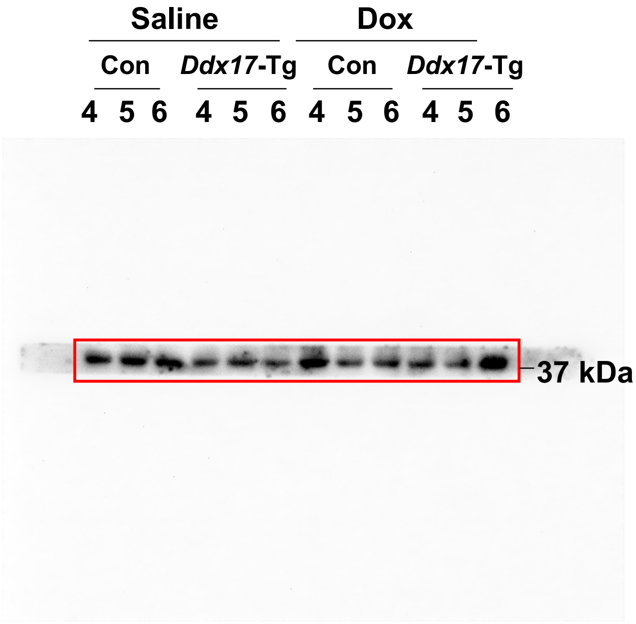


GAPDH (37 kDa)

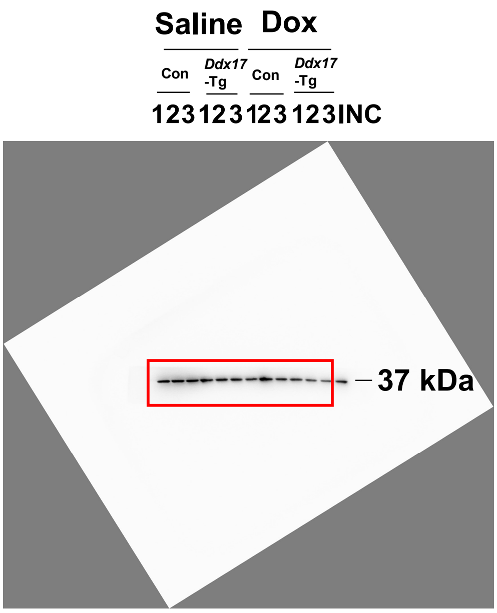


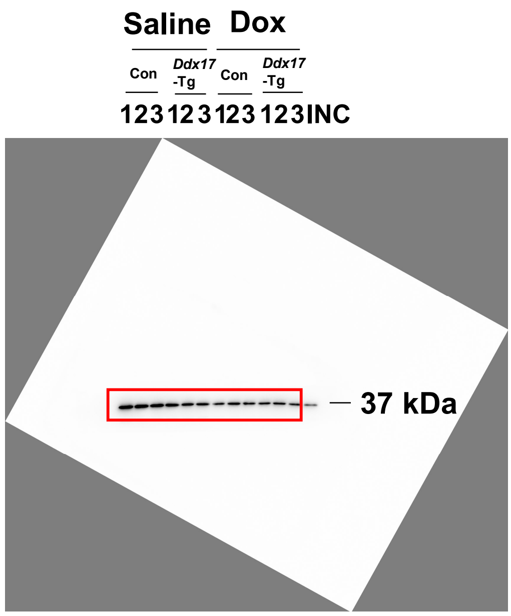

## Figure 5d

### DDX17 (82 and 72 kDa)

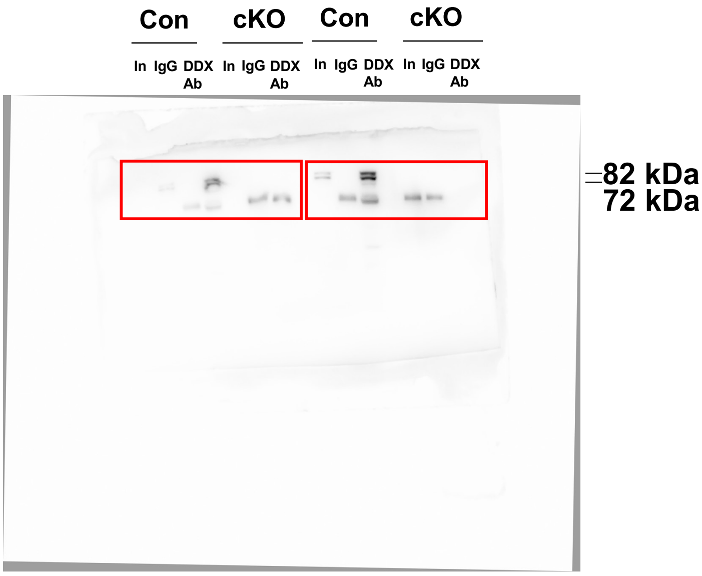


### BCL6 (87 kDa)

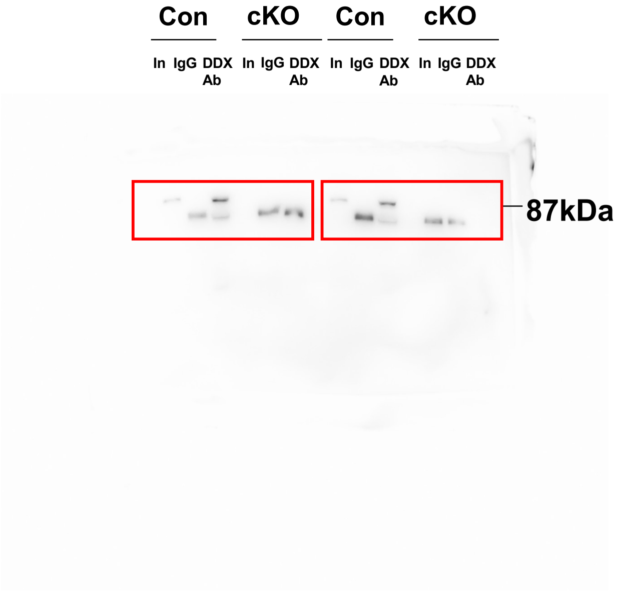


## Figure 5e

### DDX17 (82 and 72 kDa)

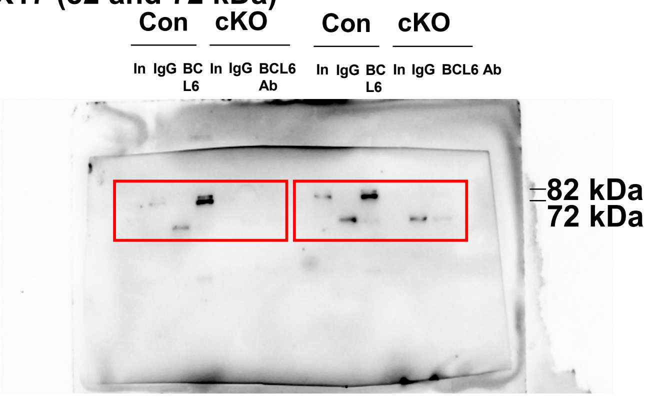


### BCL6 (87 kDa)

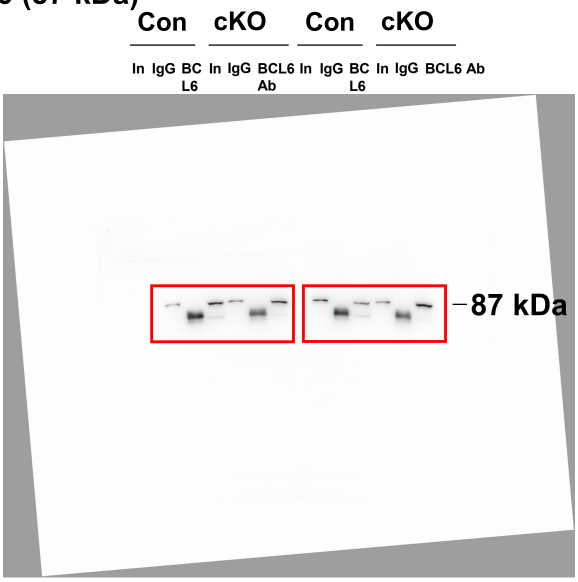


## Figure 5k

### DDX17 (82 and 72 kDa)

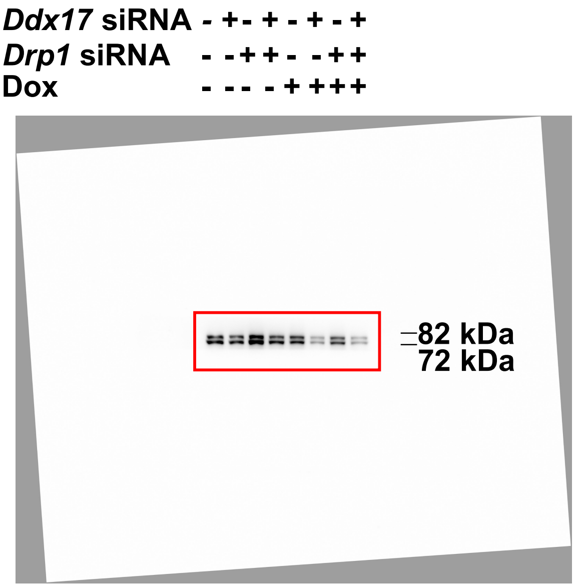


### DRP1 (80 kDa)

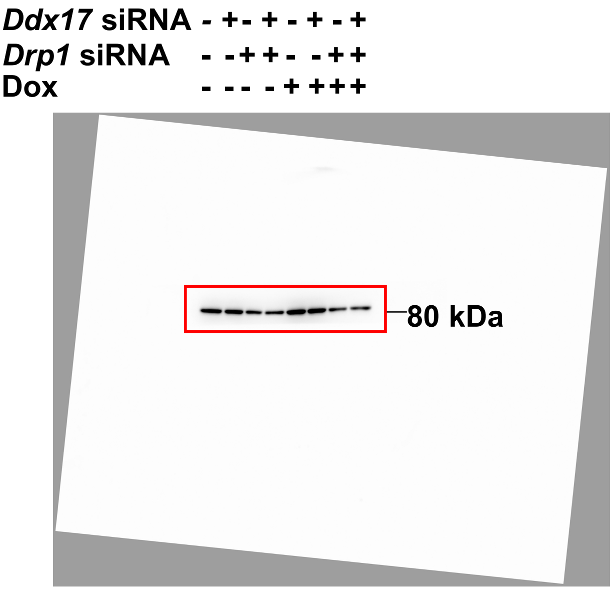

Figure 5k

DDX17 (82 and 72 kDa)

*Ddx17* siRNA - +- + - + - +
*Drp1* siRNA - -++- -++
Dox - -- - ++++

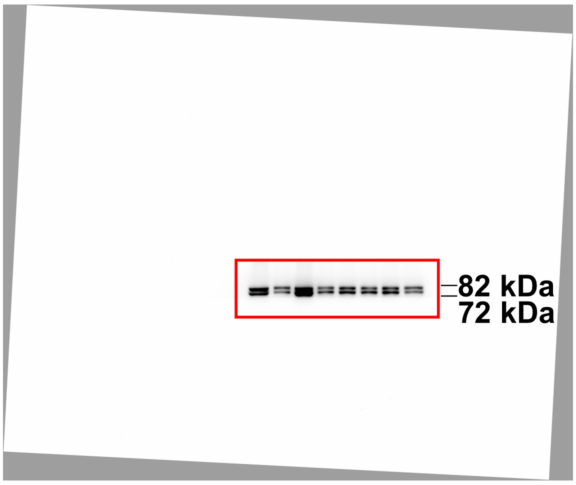


DRP1 (80 kDa)

*Ddx17* siRNA - +- + - + - +
*Drp1* siRNA - -++- -++
Dox - -- - ++++

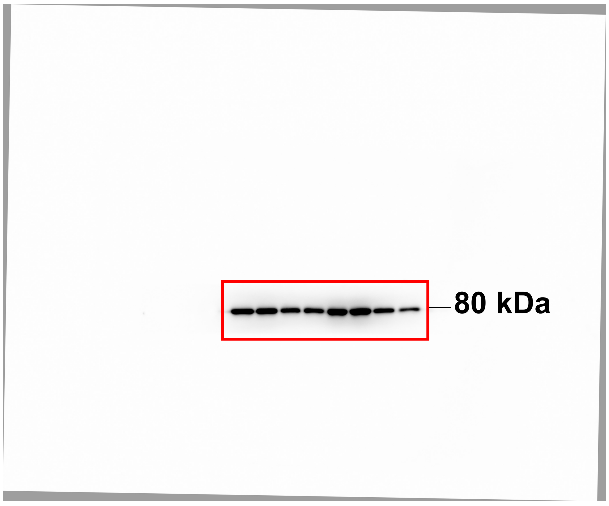


*Ddx17* siRNA - +- + - + - +
*Drp1* siRNA - -++- -++
Dox - -- - ++++

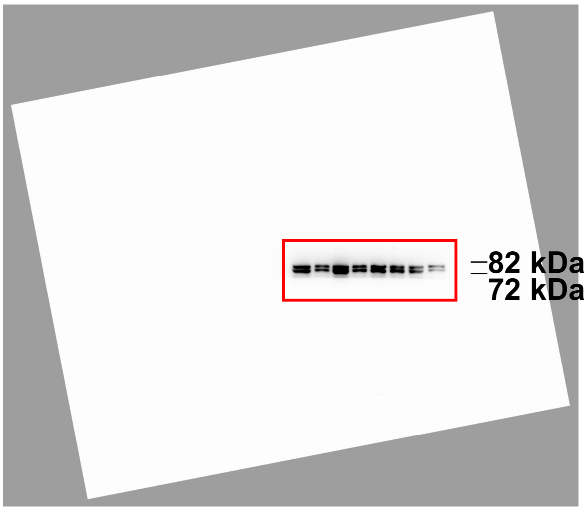


*Ddx17* siRNA - +- + - + - +
*Drp1* siRNA - -++- -++
Dox - -- - ++++

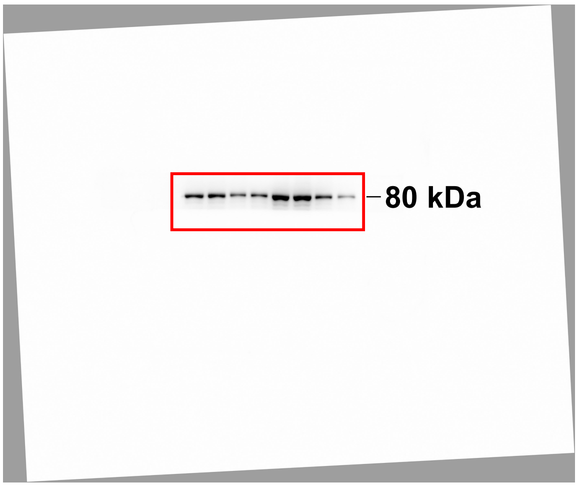


C-CASP3 (19 kDa)

*Ddx17* siRNA - +- + - + - +
*Drp1* siRNA - -++- -++
Dox - -- - ++++

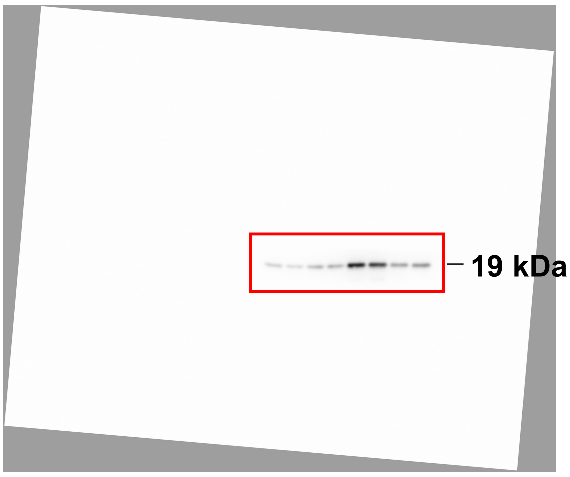


GAPDH (37 kDa)

*Ddx17* siRNA - +- + - + - +
*Drp1* siRNA - -++- -++
Dox - -- - ++++

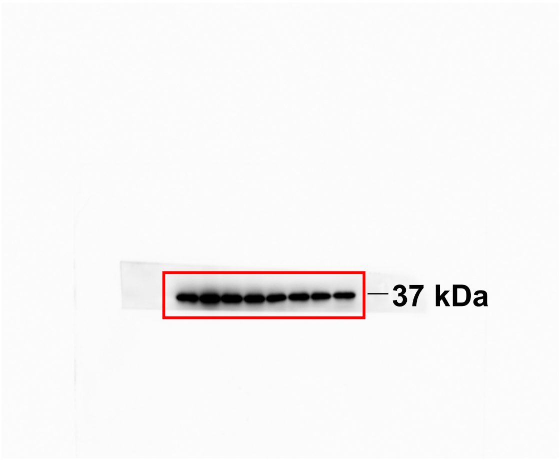

## Figure 5k

C-CASP3 (19 kDa)

*Ddx17* siRNA - +- + - + - +
*Drp1* siRNA - -++- -++
Dox - -- - ++++

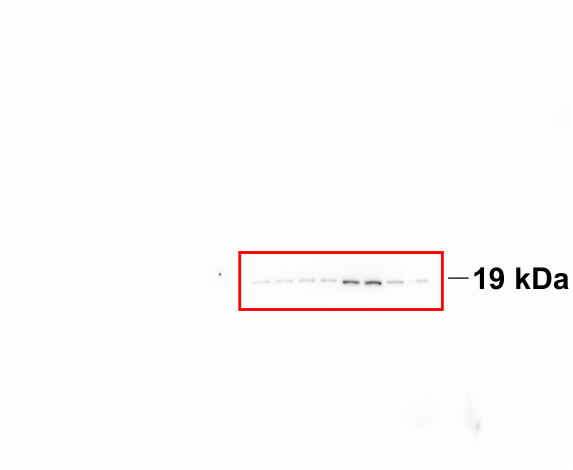


GAPDH (37 kDa)

*Ddx17* siRNA - +- + - + - +
*Drp1* siRNA - -++- -++
Dox - -- - ++++

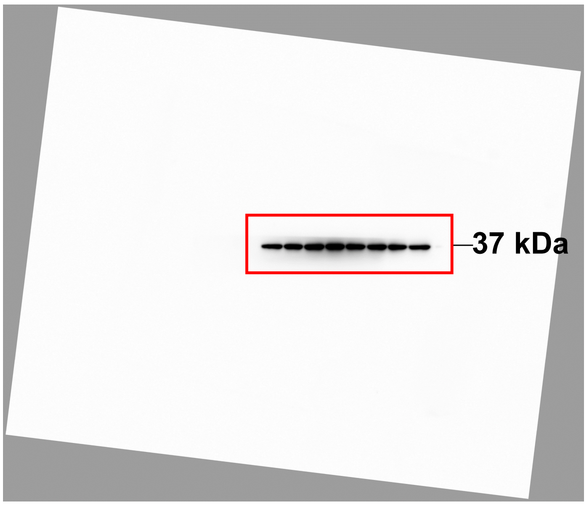


*Ddx17* siRNA - +- + - + - +
*Drp1* siRNA - -++- -++
Dox - -- - ++++

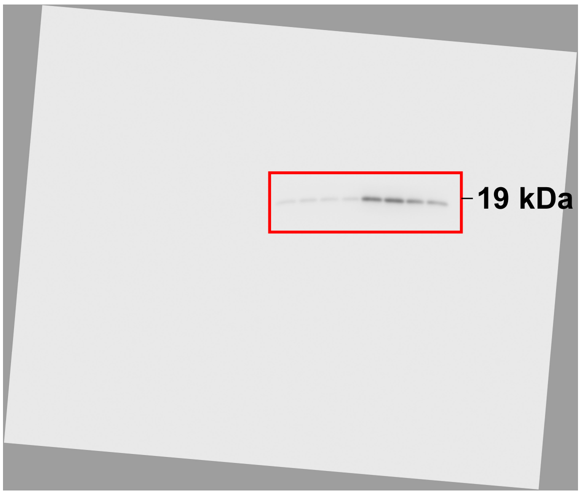


*Ddx17* siRNA - +- + - + - +
*Drp1* siRNA - -++- -++
Dox - -- - ++++

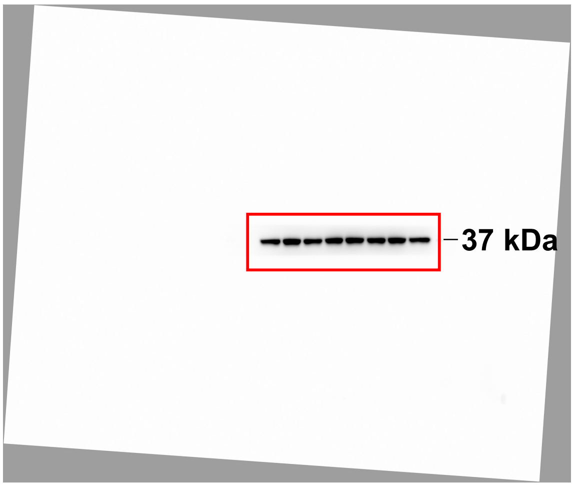


## Figure 5o

Mito-DRP1 (80 kDa)

PBS Dox PBS Dox
C KD C KD C KD C KD

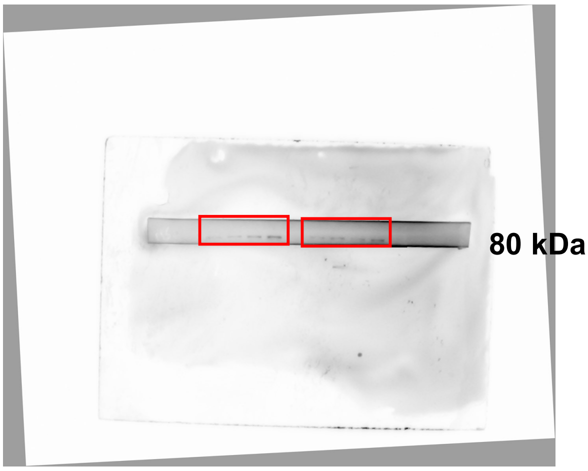


COX IV (17 kDa)

PBS Dox PBS Dox
C KD C KD C KD C KD

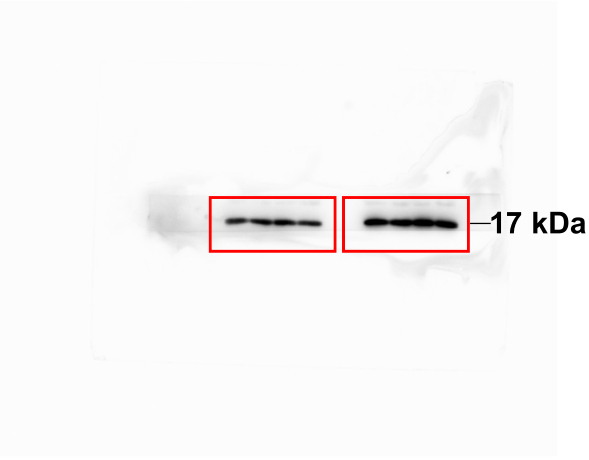

## Figure 5o

### Mito-DRP1 (80 kDa)

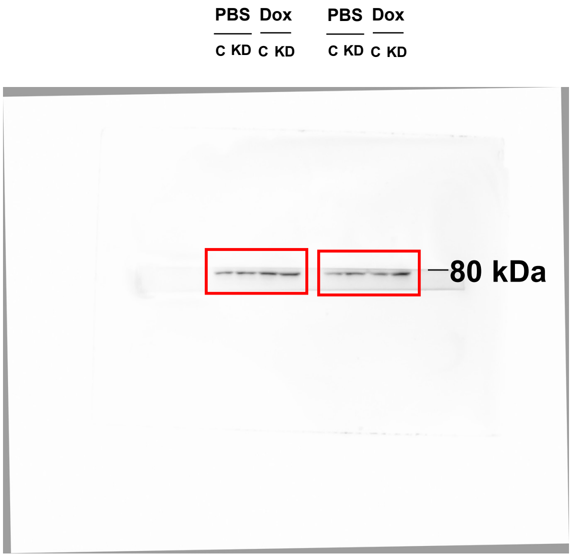


### COX IV (17 kDa)

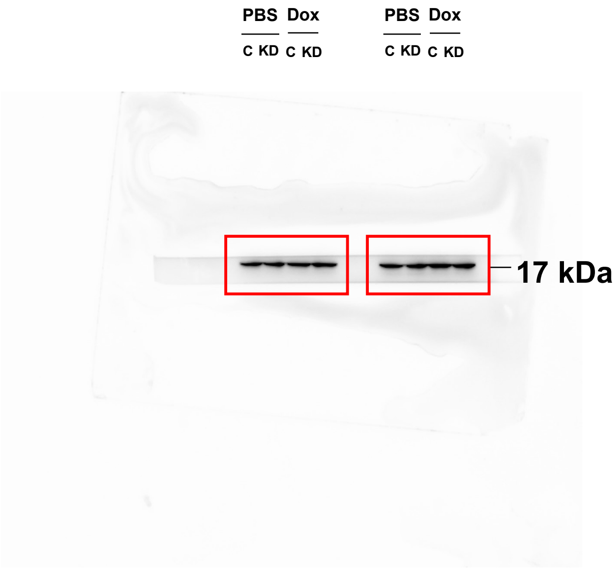


### Cyto-DRP1 (80 kDa)

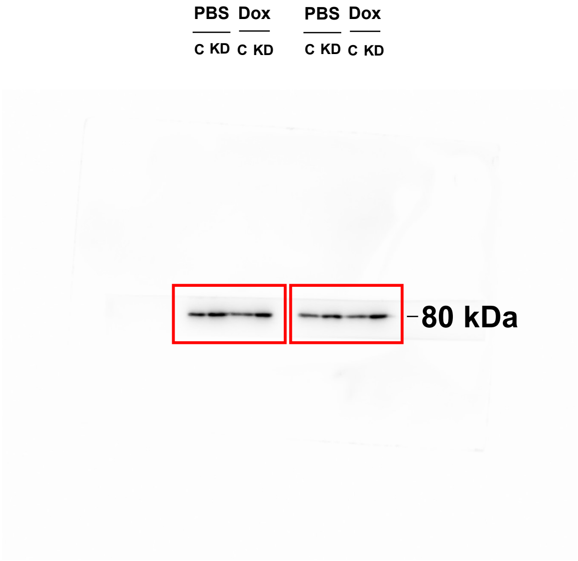


### Tubulin (55 kDa)

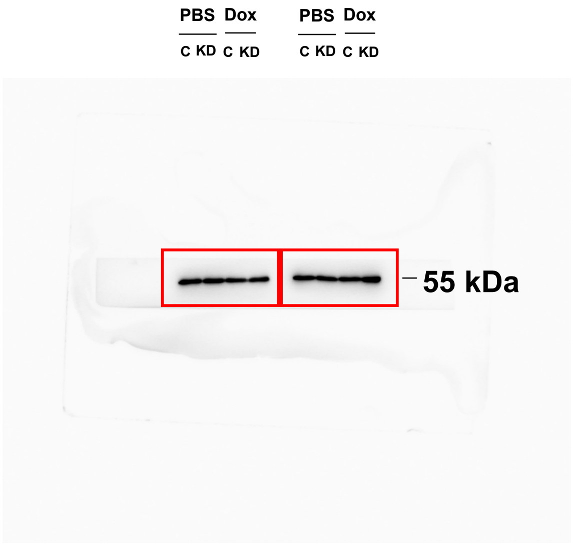


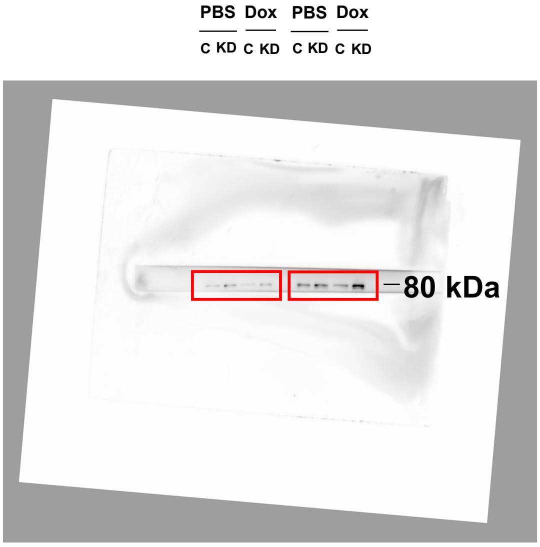


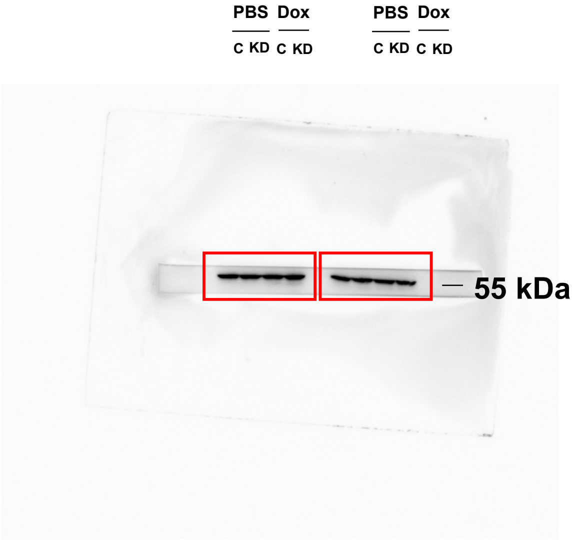

## Figure 5r

### Mito-Cyt C (14 kDa)

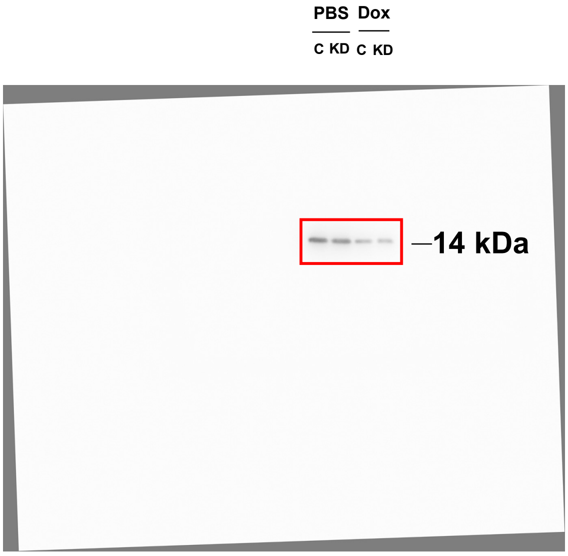


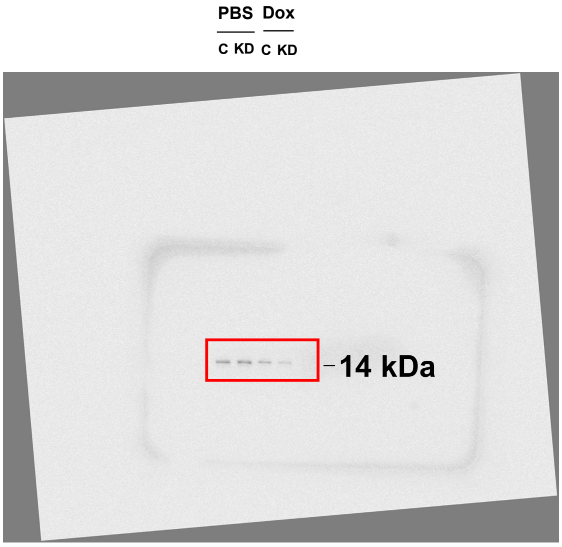


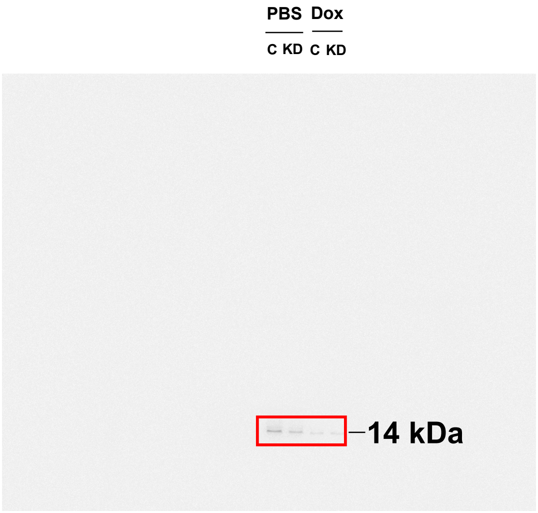


### COX IV (17 kDa)

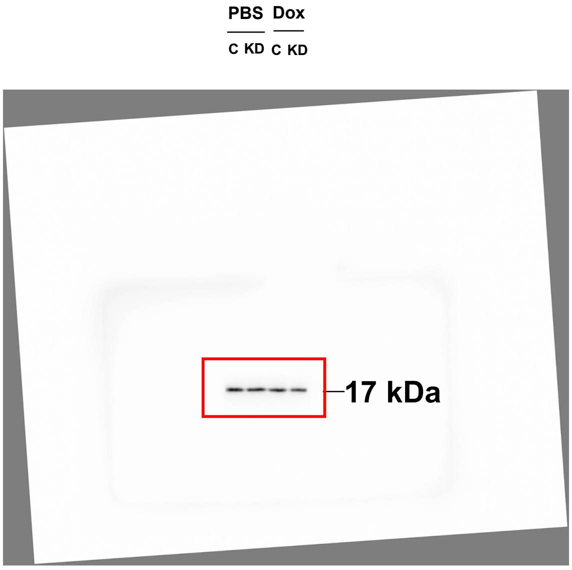


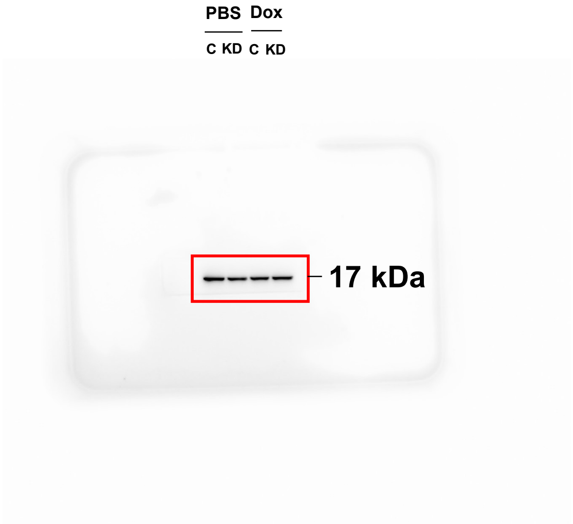


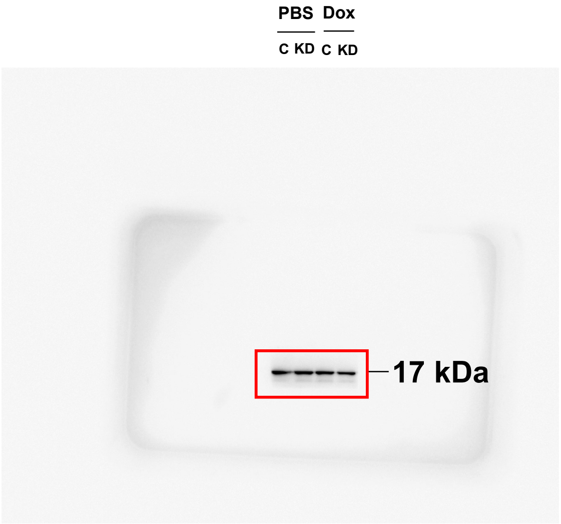

## Figure 5r

### Mito-Cyt C (14 kDa)

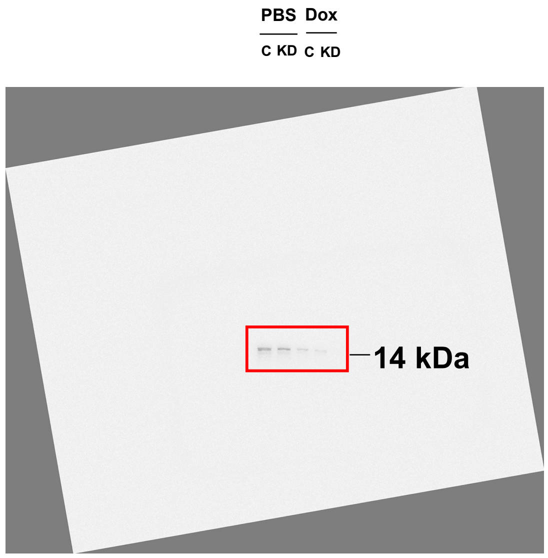


### COX IV (17 kDa)

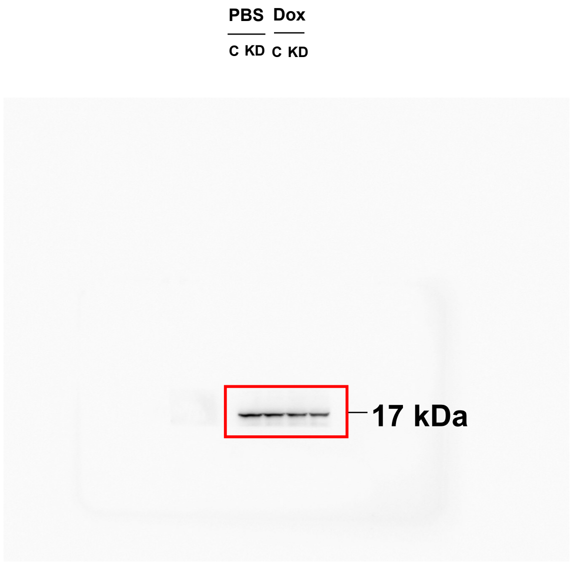


### Cyto-Cyt C (14 kDa)

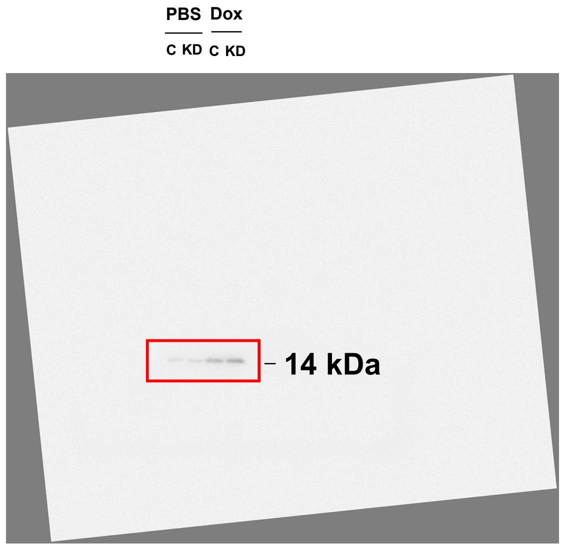


### Tubulin (55 kDa)

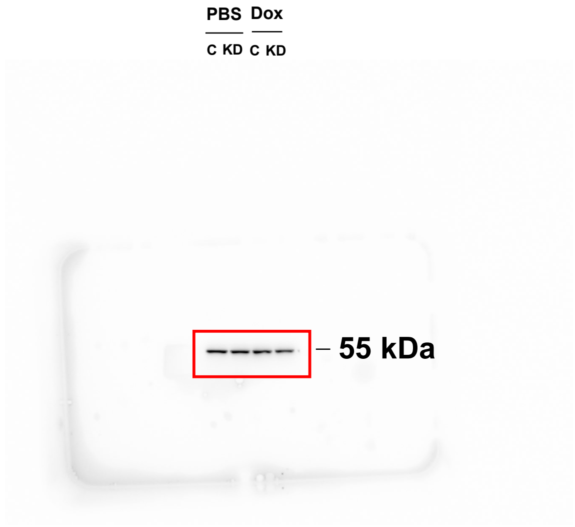


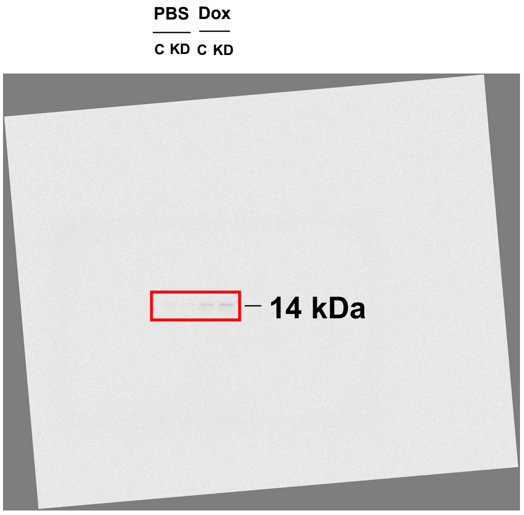


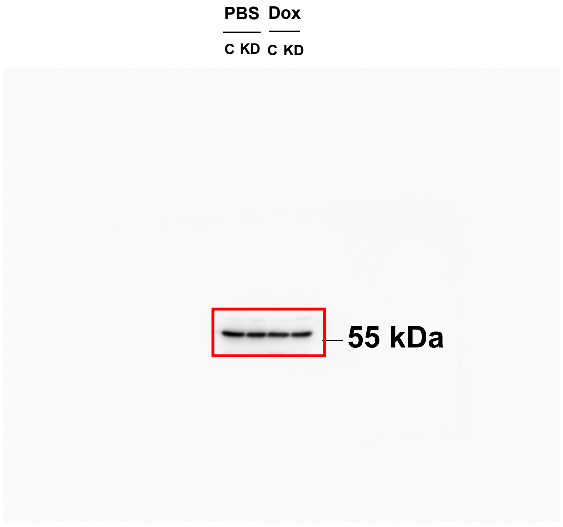

## Figure 5r

### Cyto-Cyt C (14 kDa)

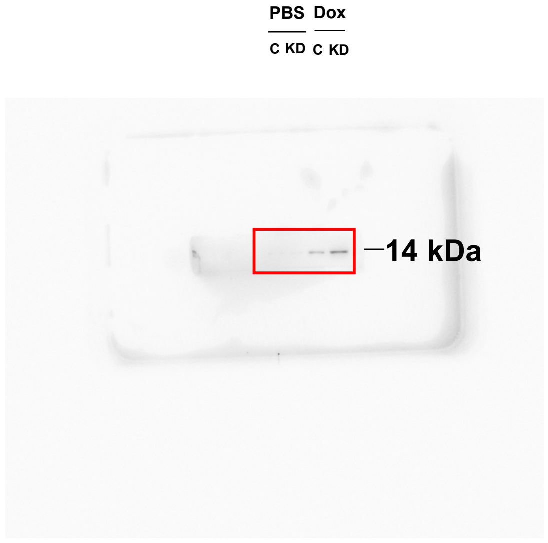


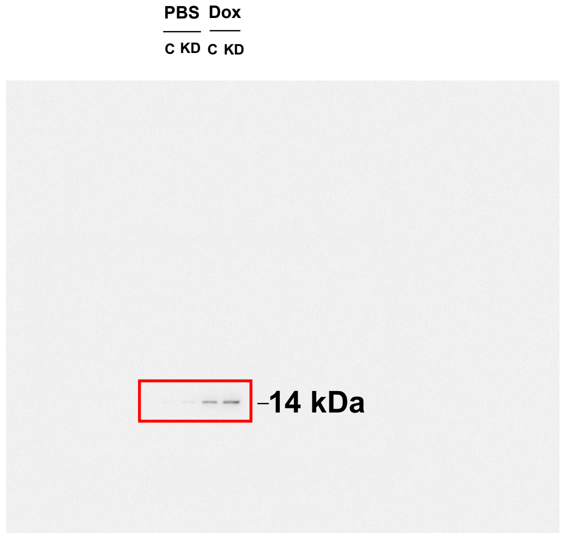


### Tubulin (55 kDa)

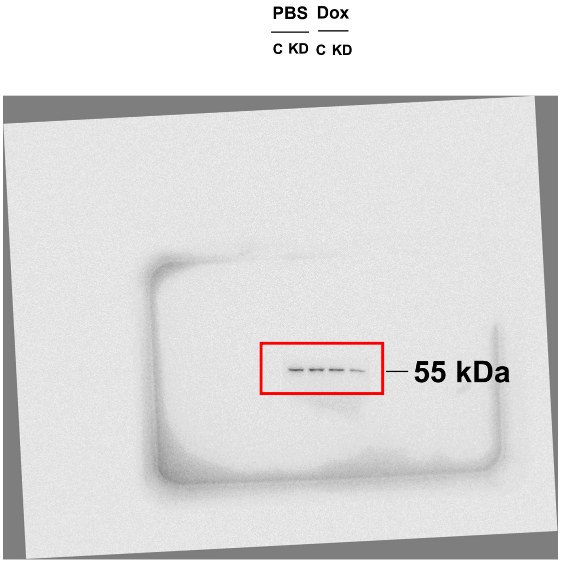


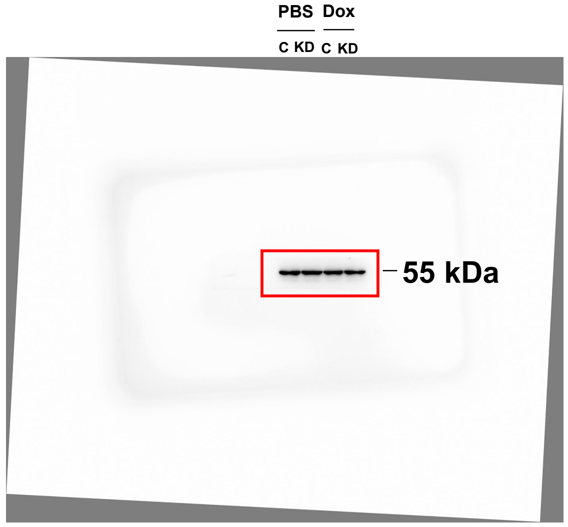

**Supplementary Figure 1a**

**CASP-3 (35 kDa) and c-CASP3 (19 kDa)**

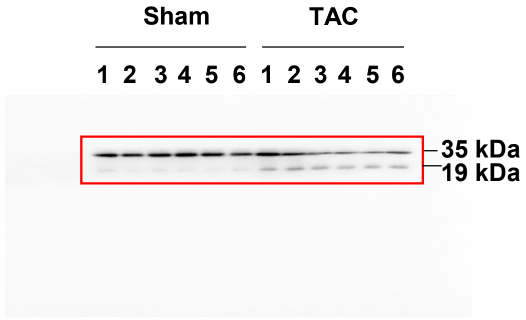


**BCL2 (26 kDa)**

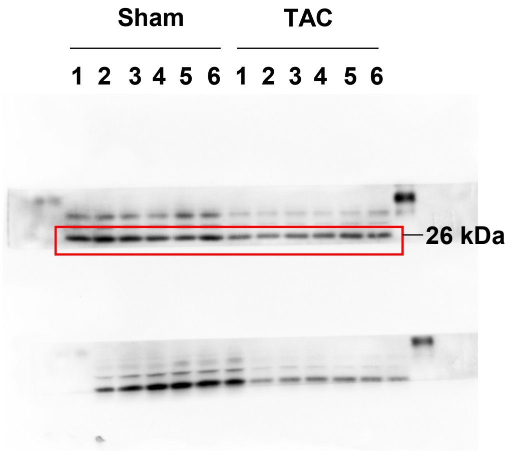


**BAX (21 kDa)**

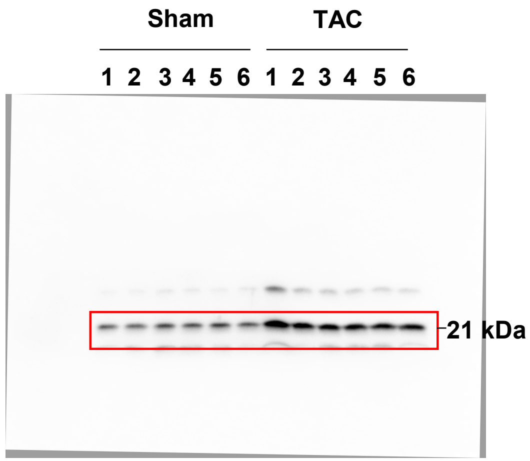


**GAPDH (37 kDa)**

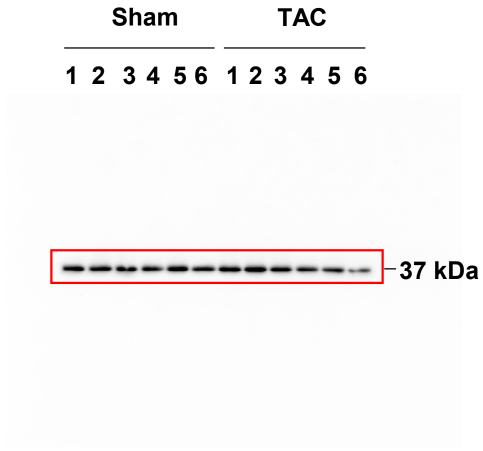


**Supplementary Figure 1m**

**LC3B (18 and 16 kDa)**

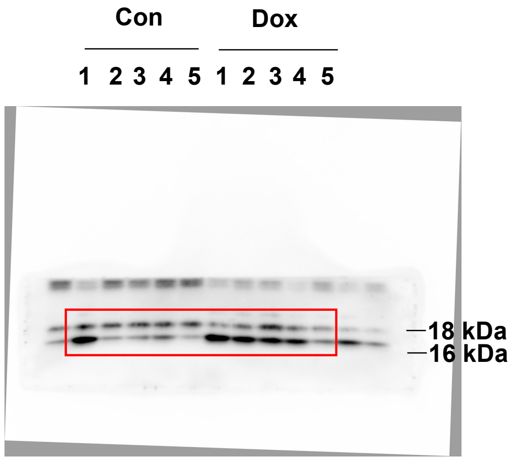


**p62 (62 kDa)**

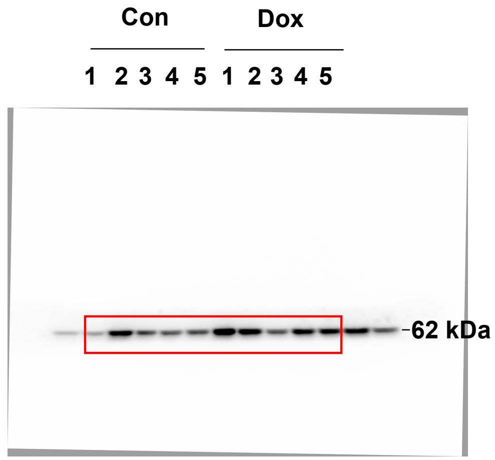


**GAPDH (37 kDa)**

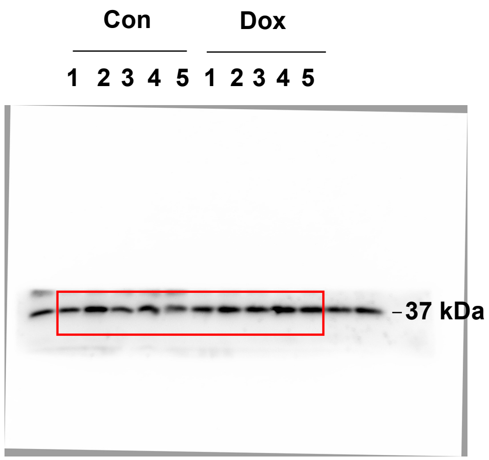


**Supplementary Figure 1r**

**CASP-3 (35 kDa)**

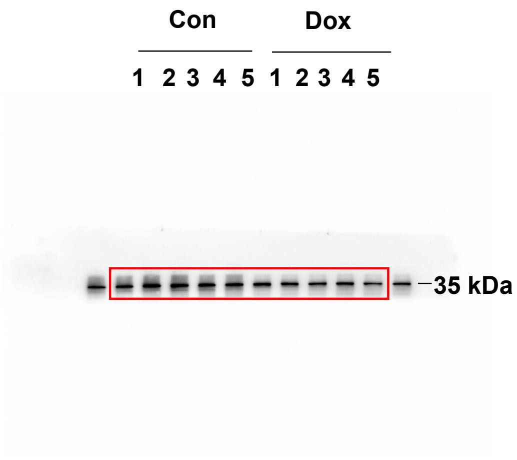

Supplementary Figure 1r

c-CASP-3 (19 kDa)

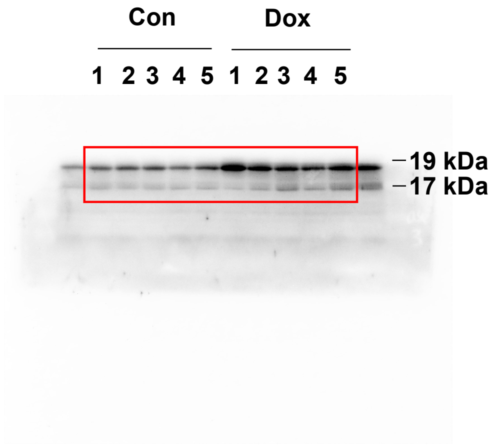


BCL2 (26 kDa)

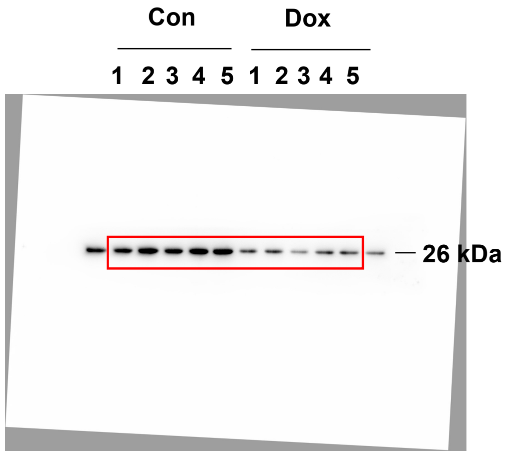


BAX (21 kDa)

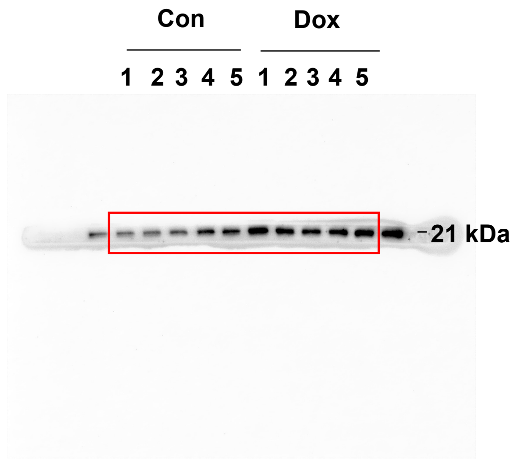


GAPDH (37 kDa)

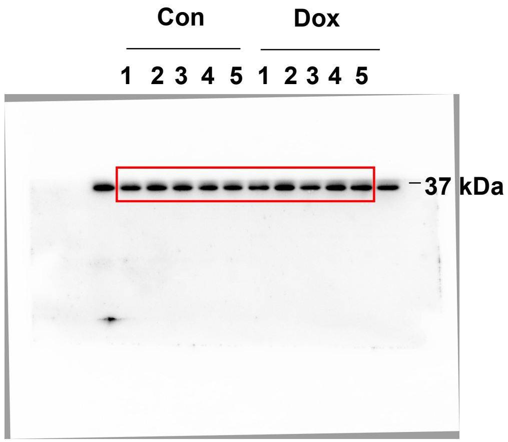


Supplementary Figure 2c

LC3B (18 and 16 kDa)

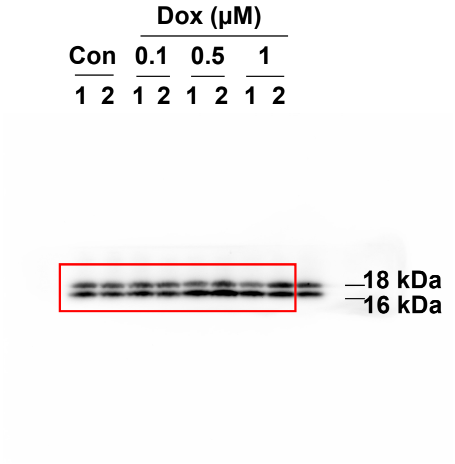


p62 (62 kDa)

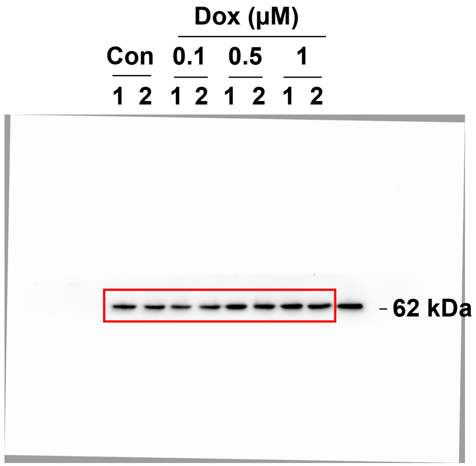


GAPDH (37 kDa)

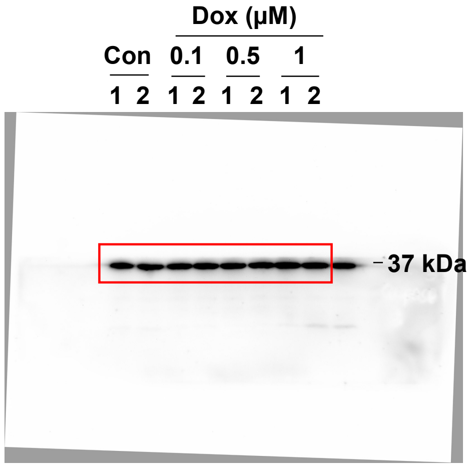

Supplementary Figure 2c

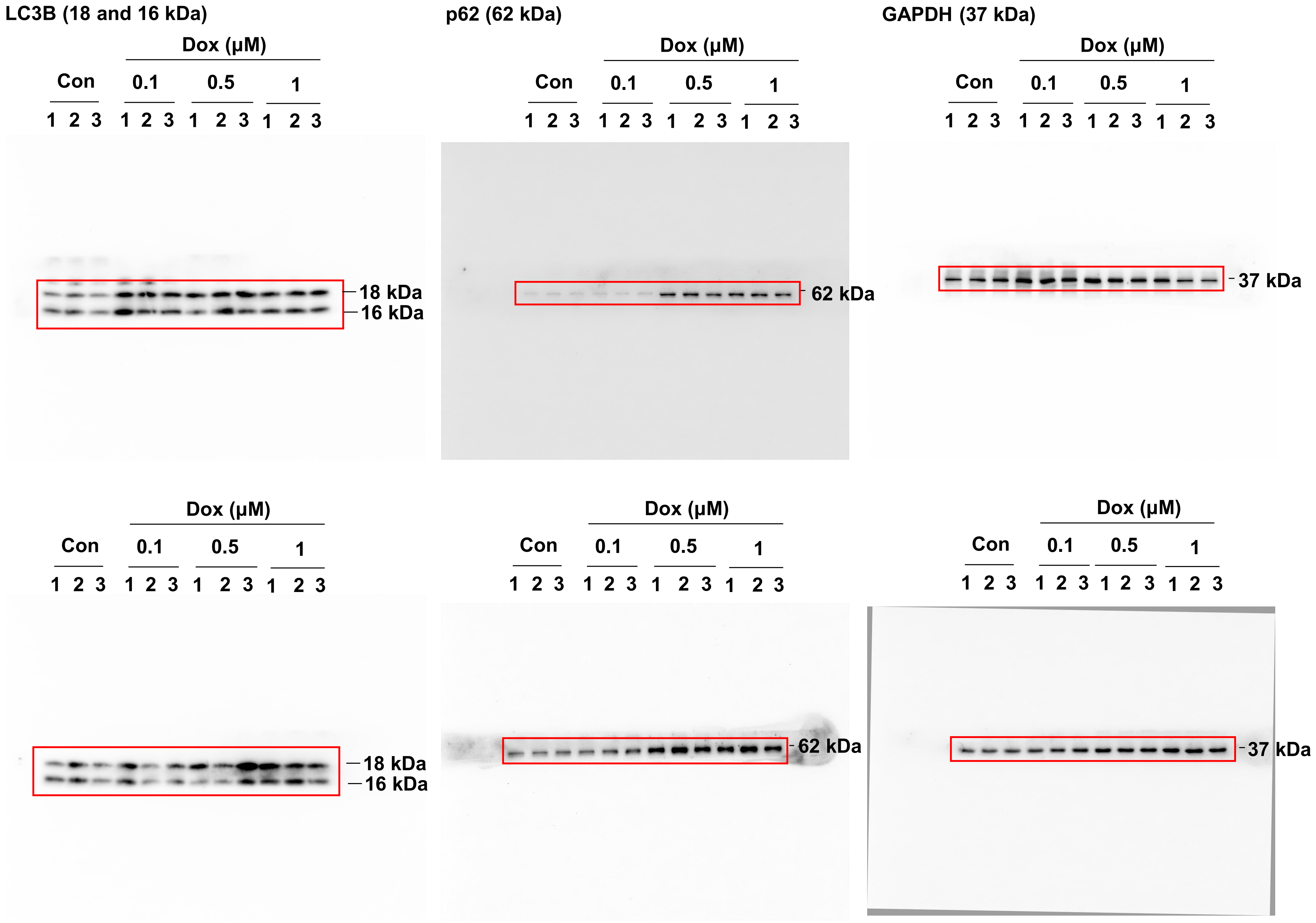

## Supplementary Figure 2h

### CASP-3 (35 kDa)

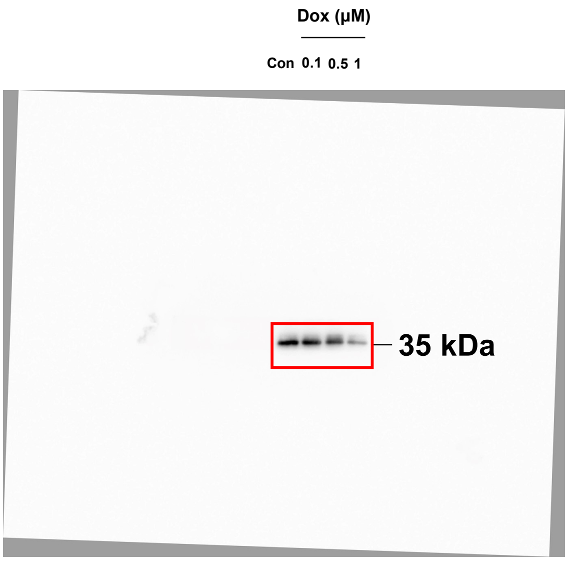


### C-CASP-3 (19 kDa)

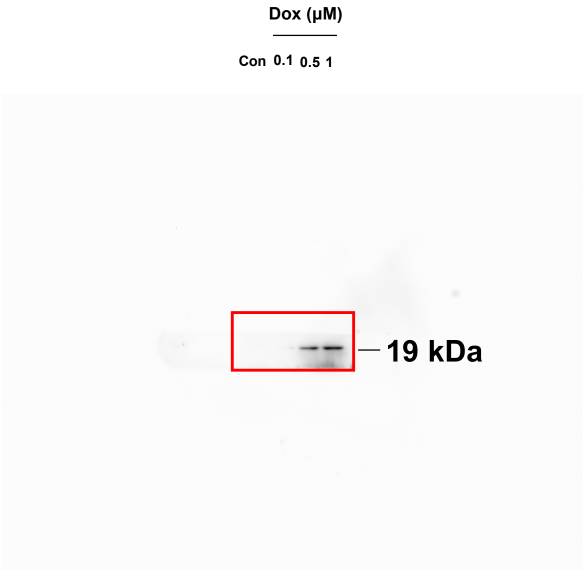

**Supplementary Figure 2h**

**CASP-3 (35 kDa)**

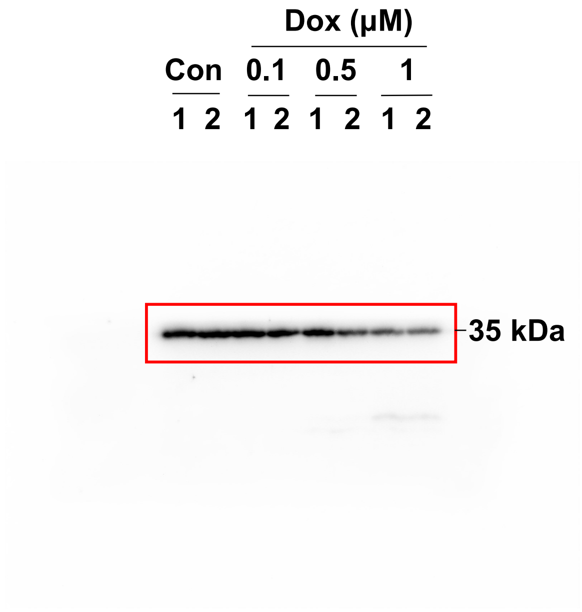


**C-CASP-3 (19 kDa, 17kDa)**

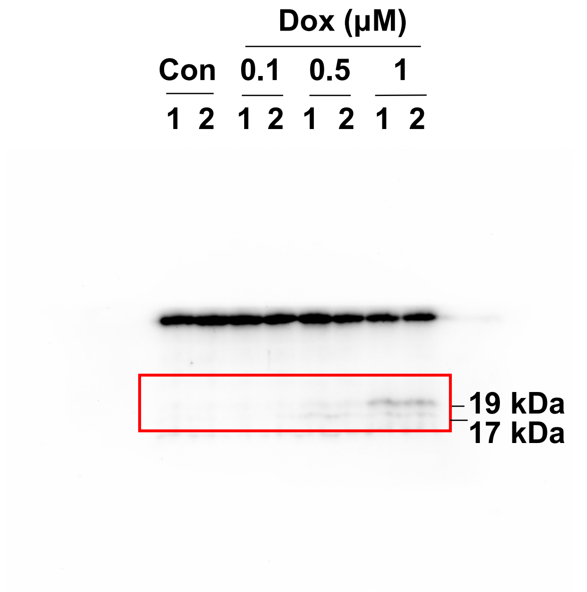


**CASP-3 (35 kDa) and C-CASP-3 (19 and 17 kDa)**

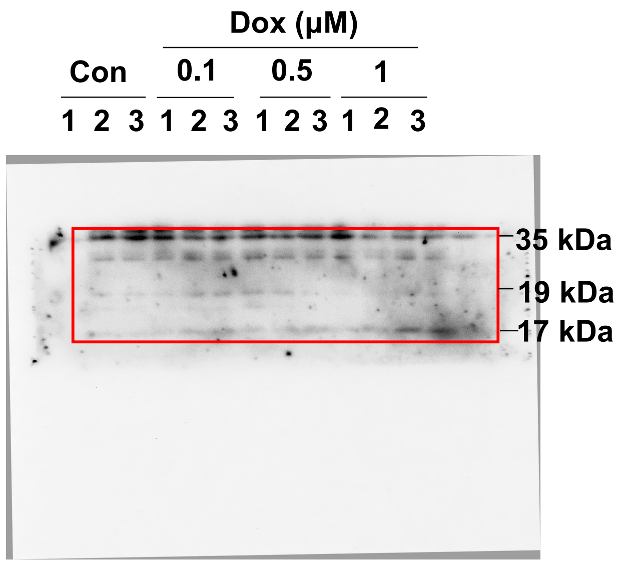


**CASP-3 (35 kDa) and C-CASP-3 (19 and 17 kDa)**

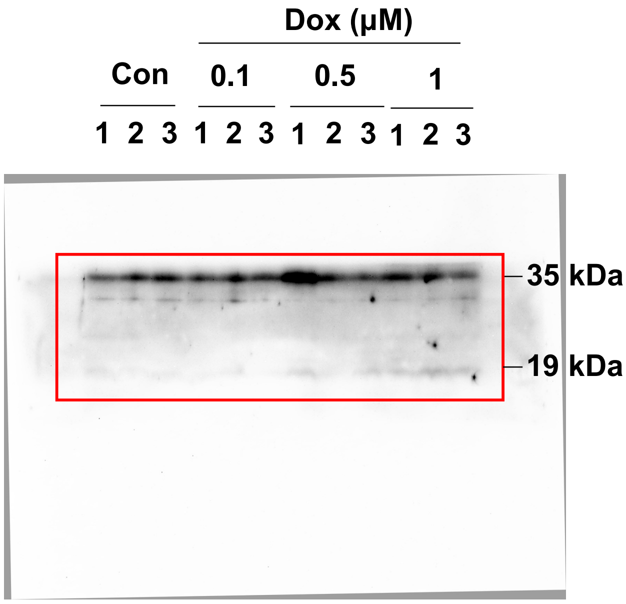


**BCL2 (26 kDa)**

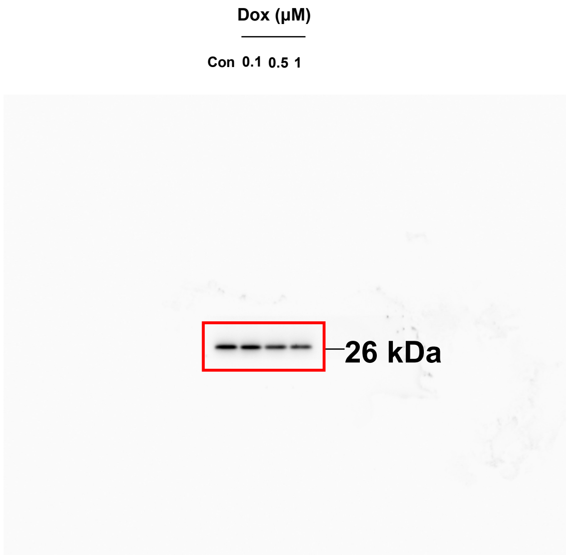


**BAX (21 kDa)**

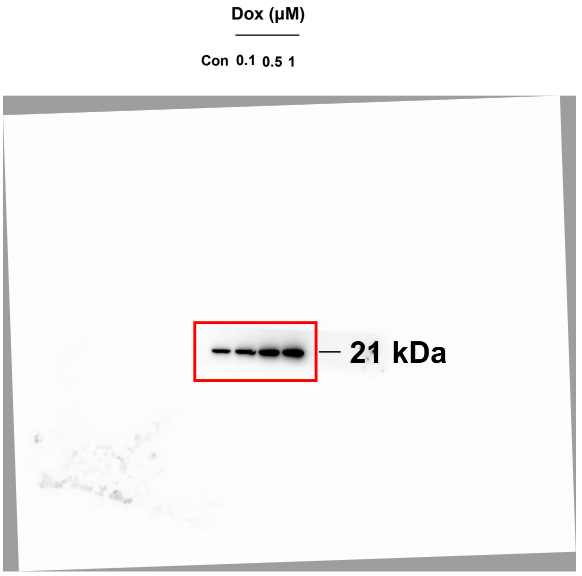

## Supplementary Figure 2h

### BCL2 (26 kDa)

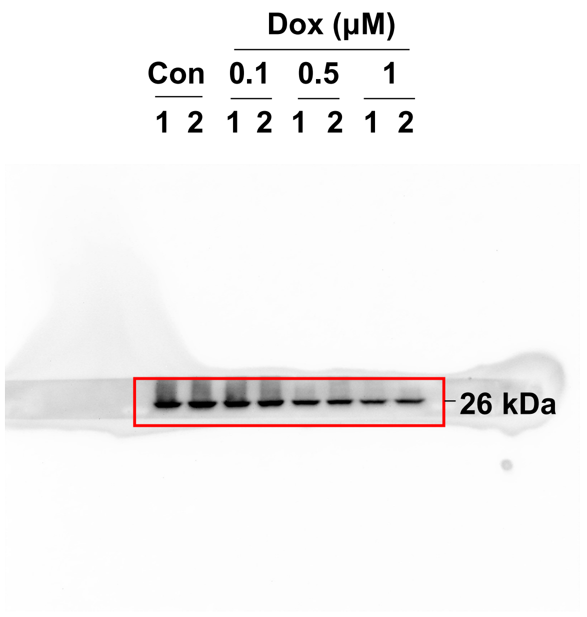


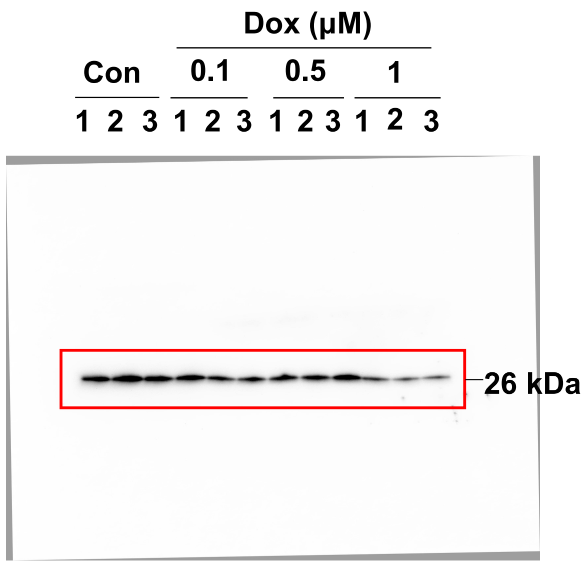


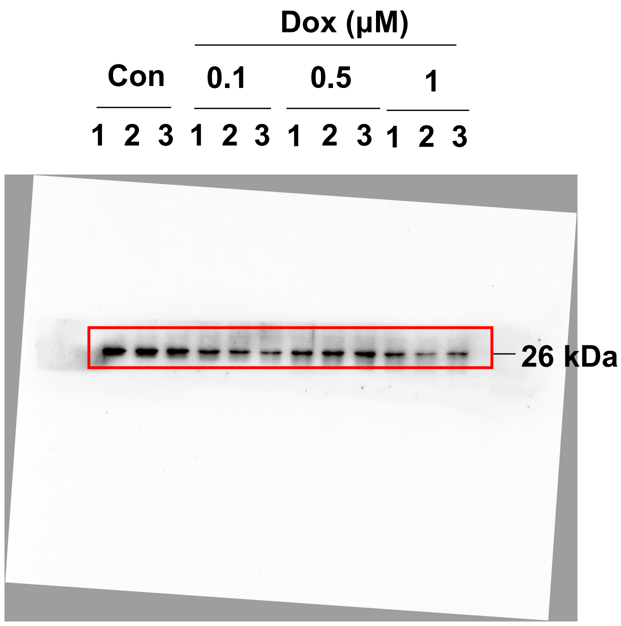


### BAX (21 kDa)

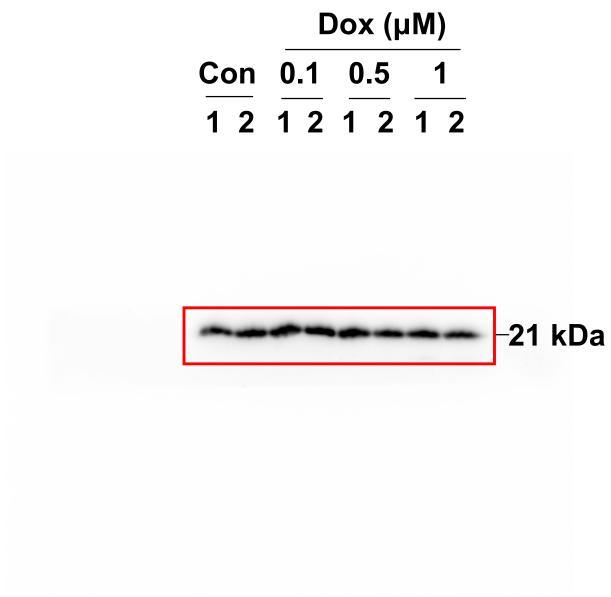


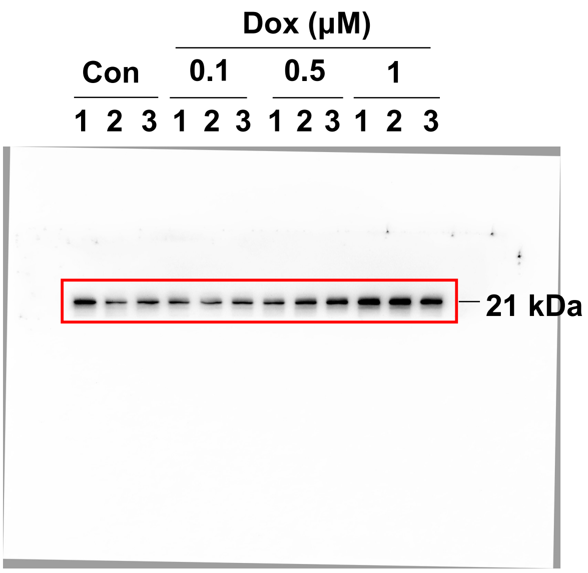


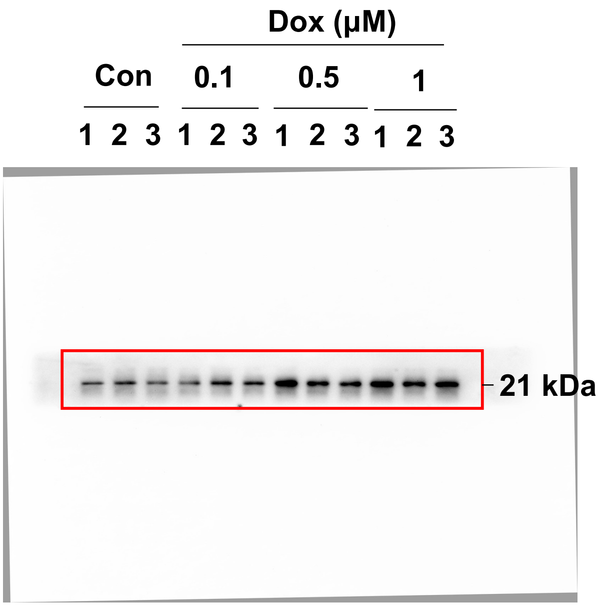

## Supplementary Figure 2h

### GAPDH (37 kDa)

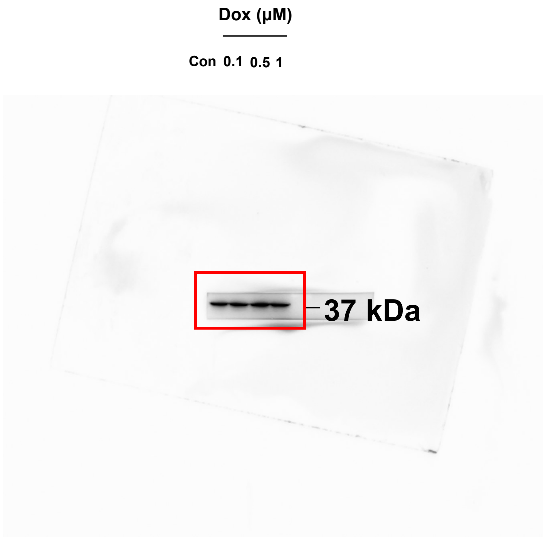


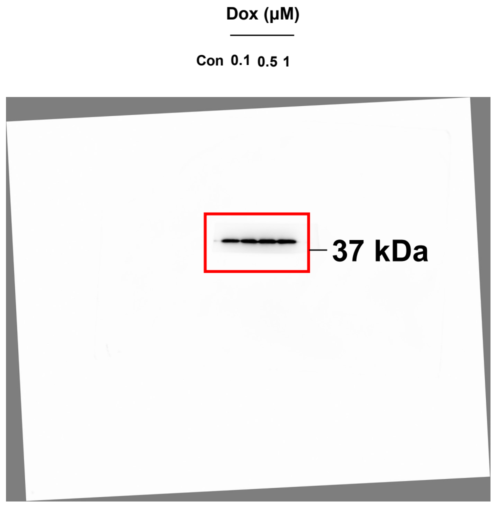


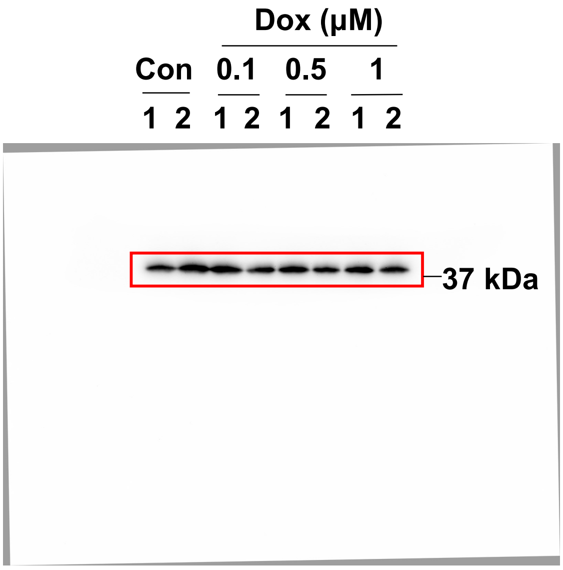


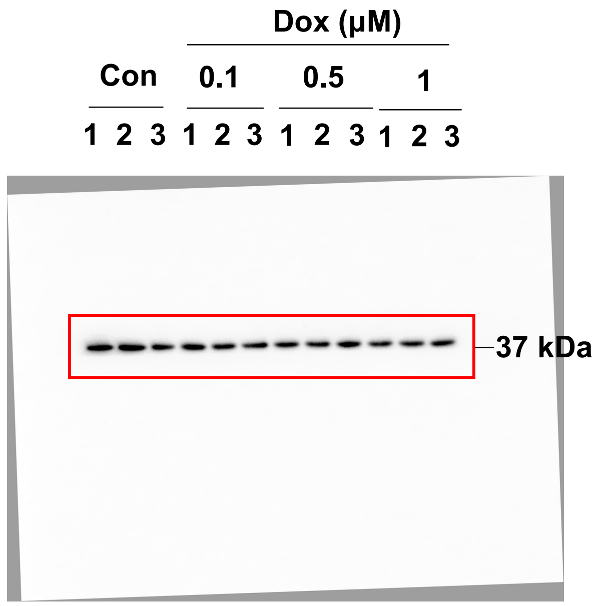


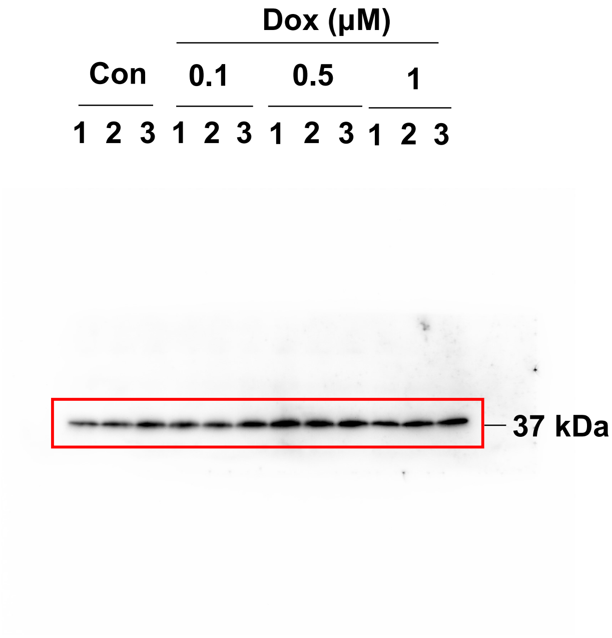

Supplementary Figure 5a

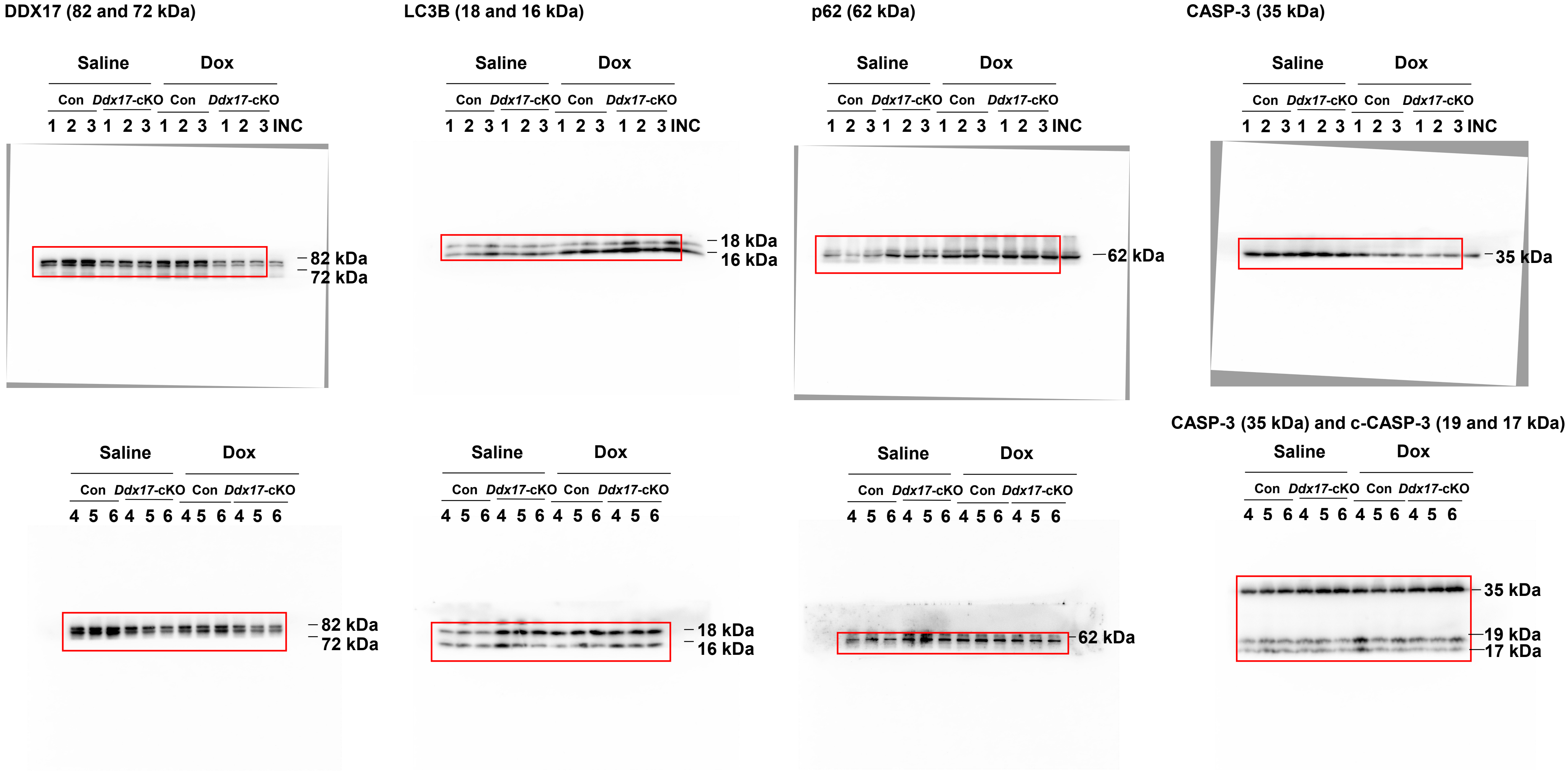

Supplementary Figure 5a

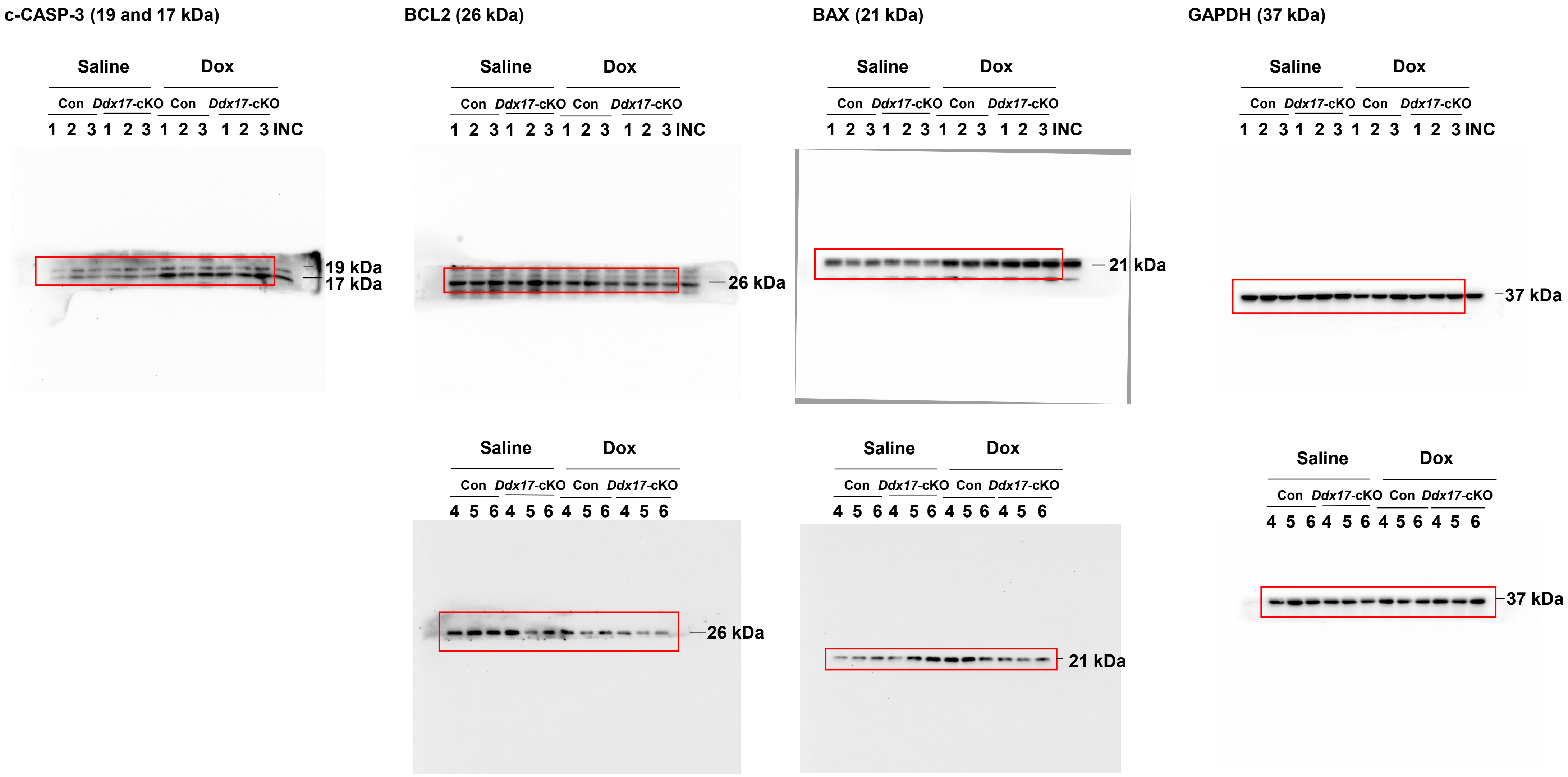

Supplementary Figure 5g

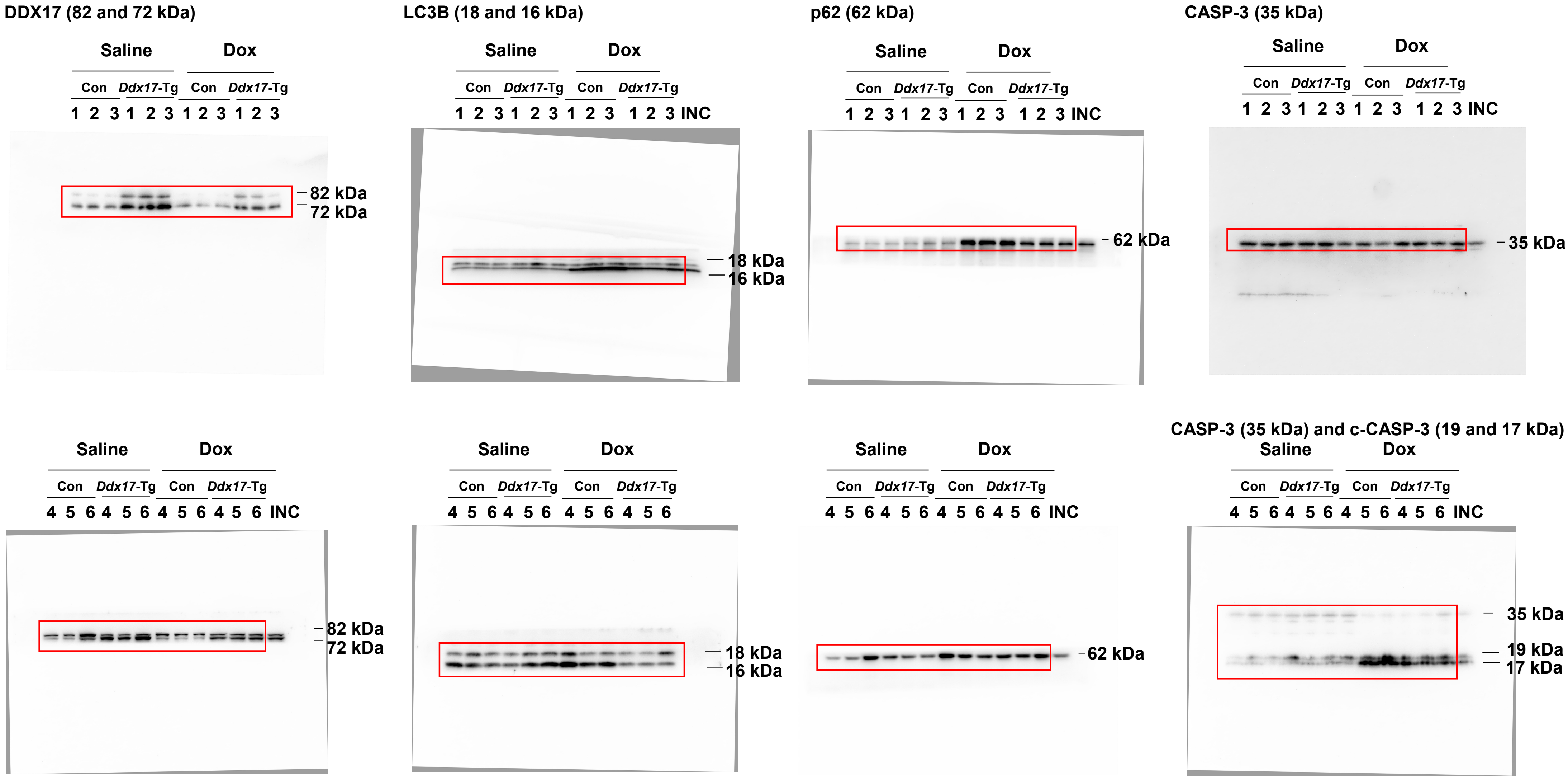

Supplementary Figure 5g

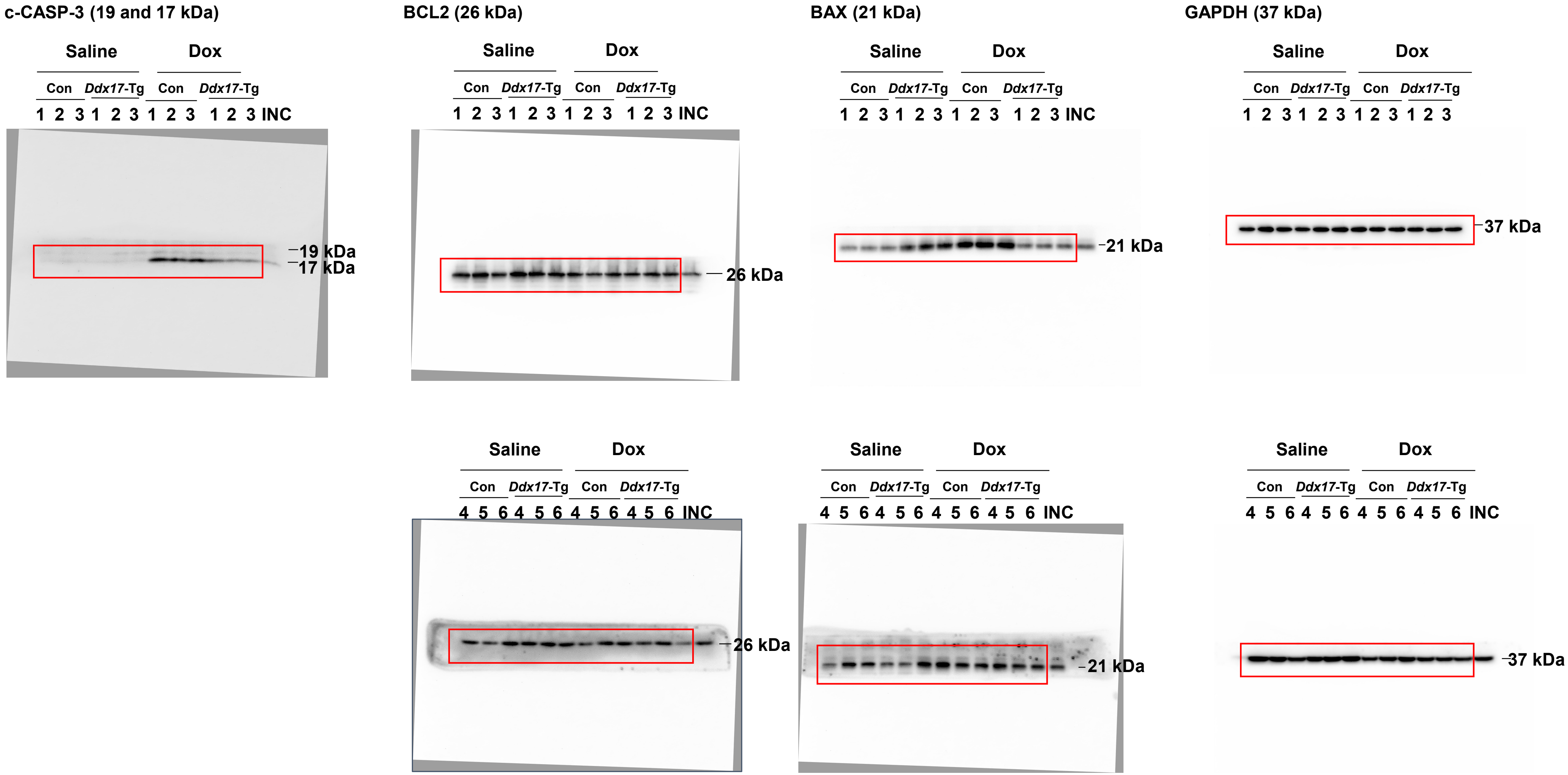

Supplementary Figure 6a

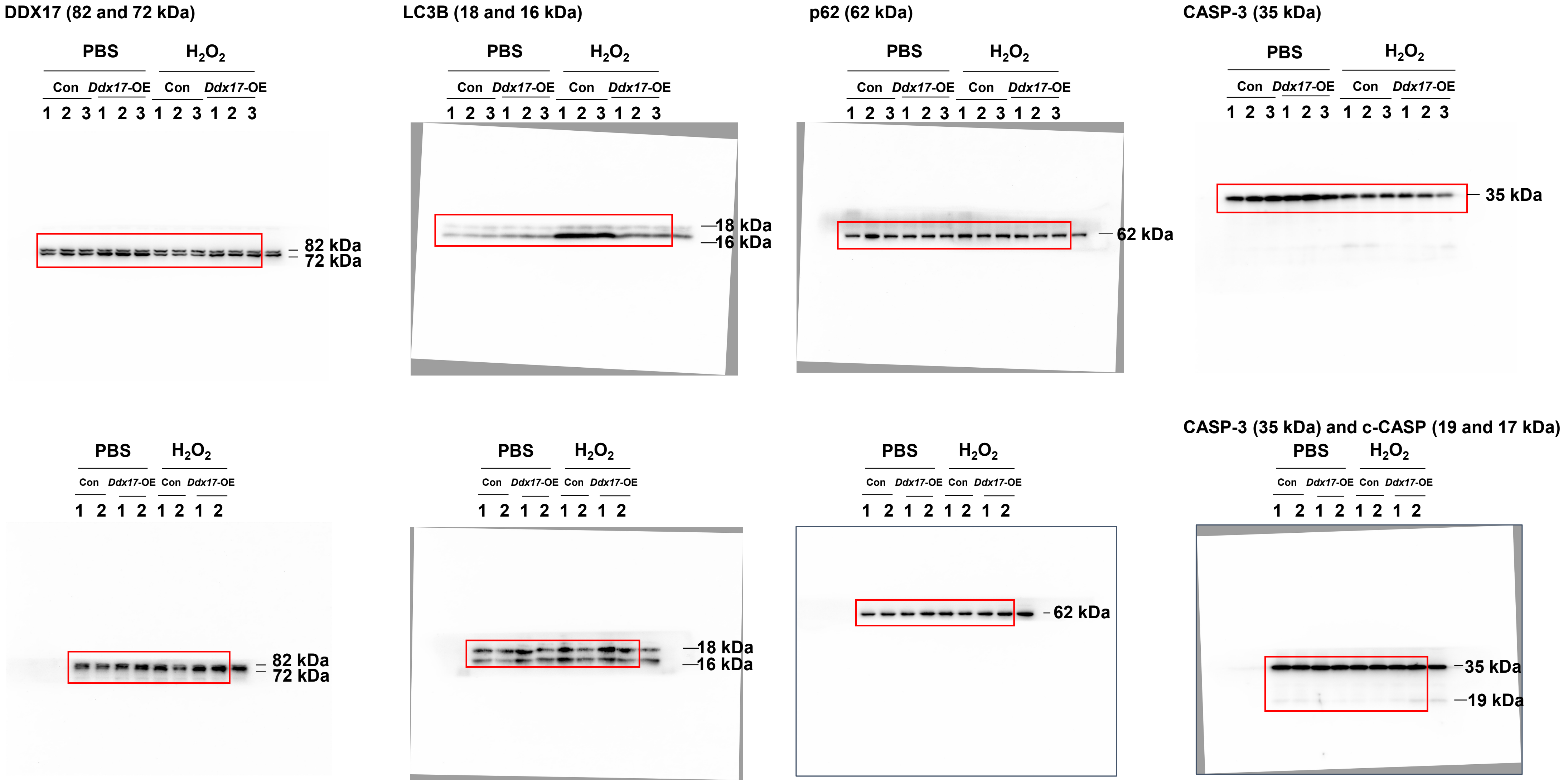

**Supplementary Figure 6a**

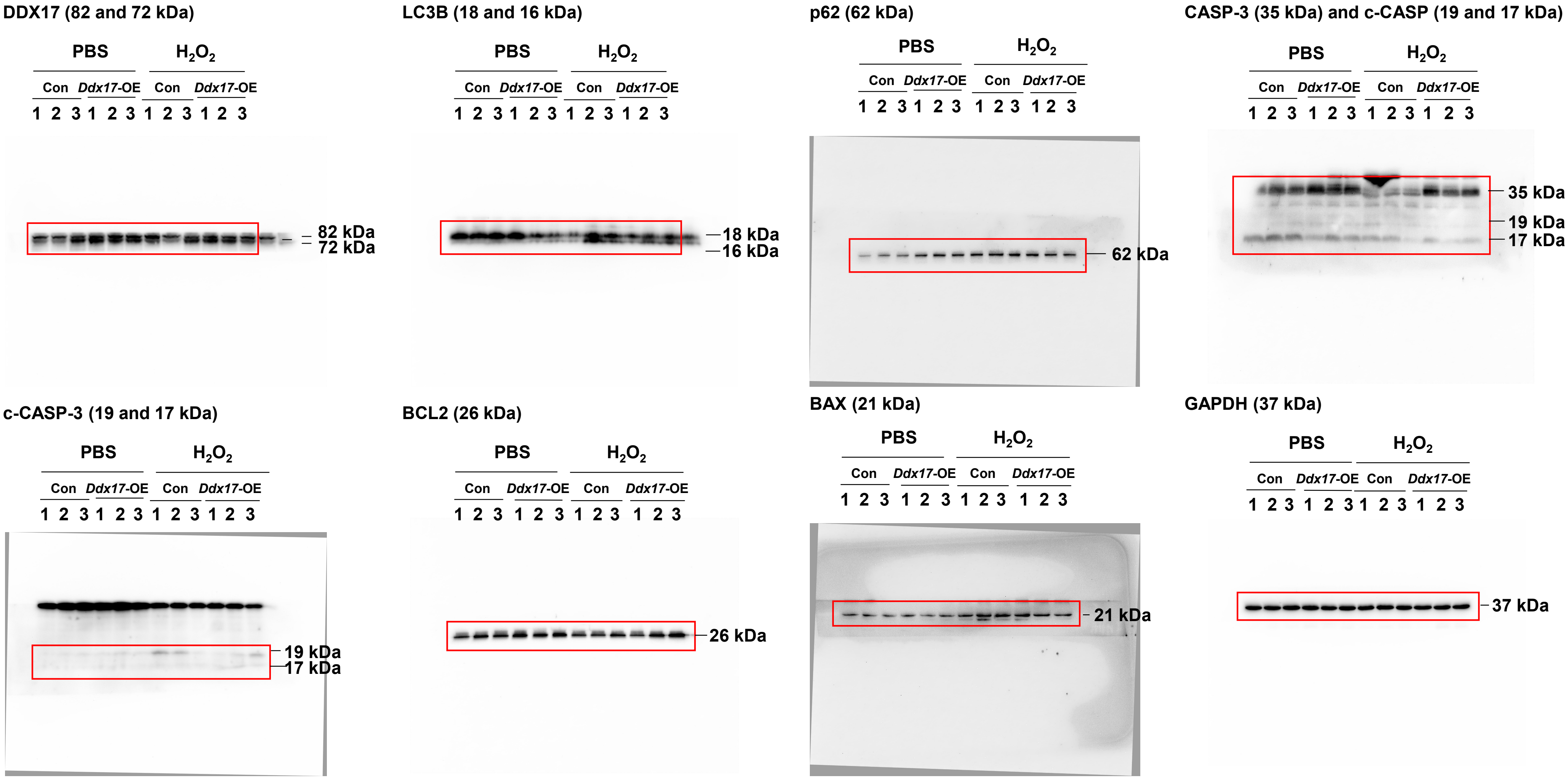

## Supplementary Figure 6a

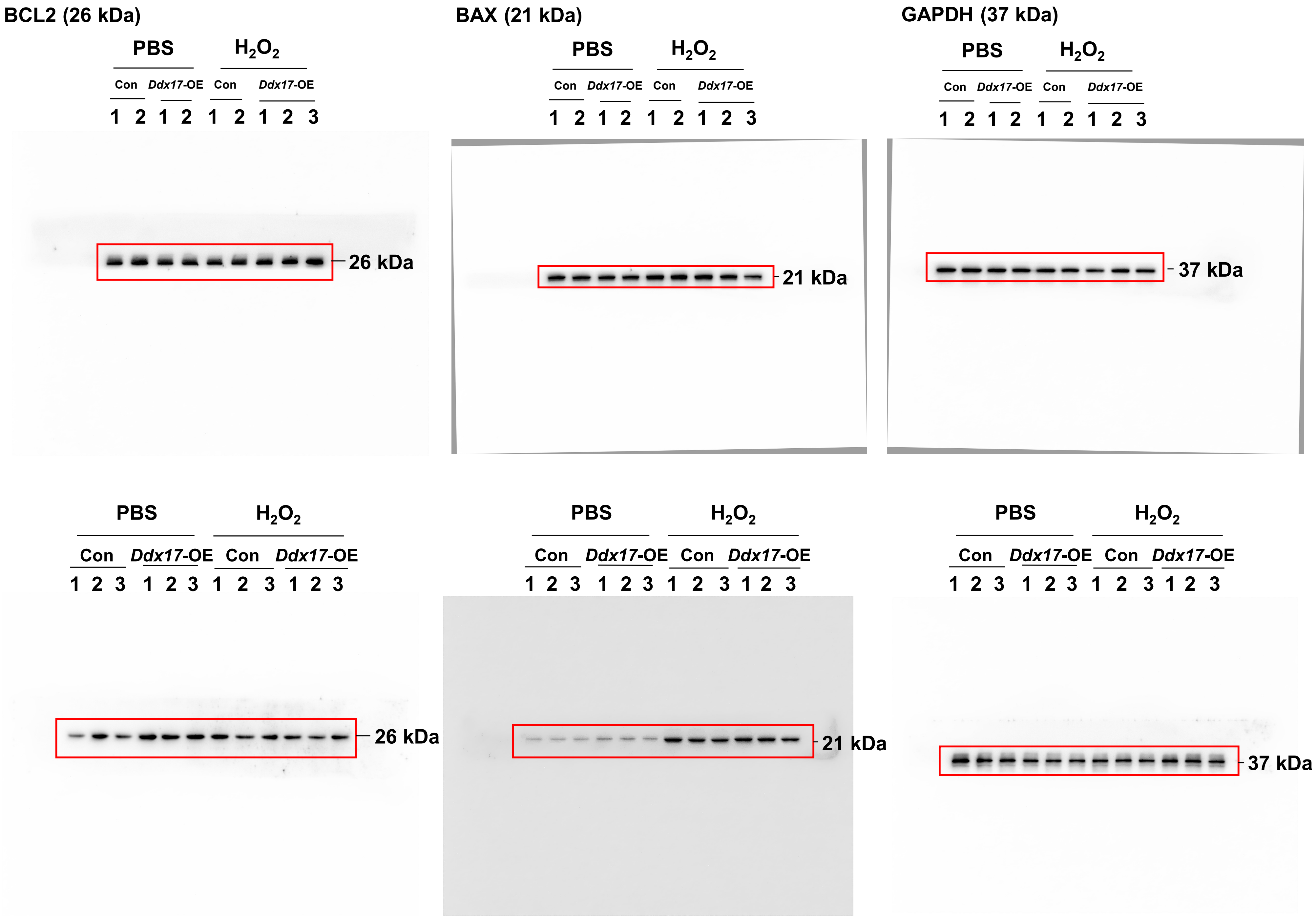

## Supplementary Figure 6g

DDX17 (82 and 72 kDa)

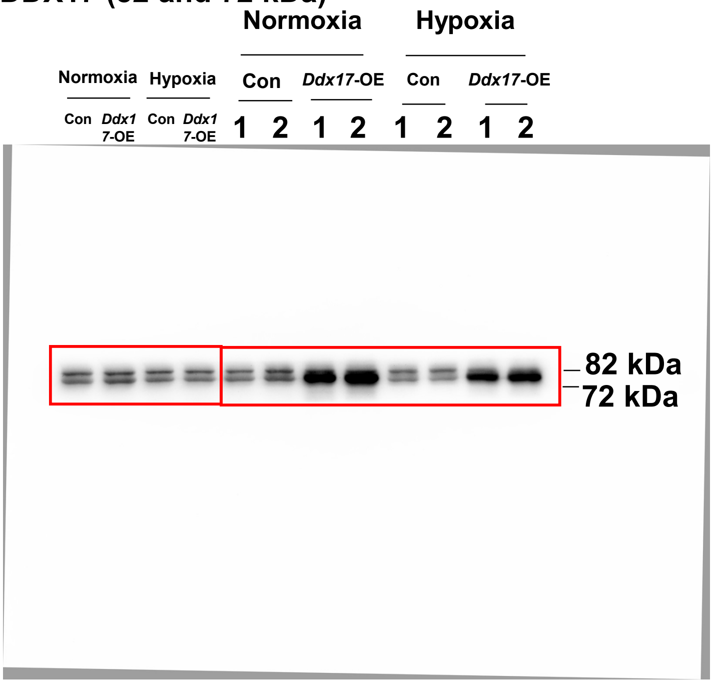


CASP-3 (35 kDa)

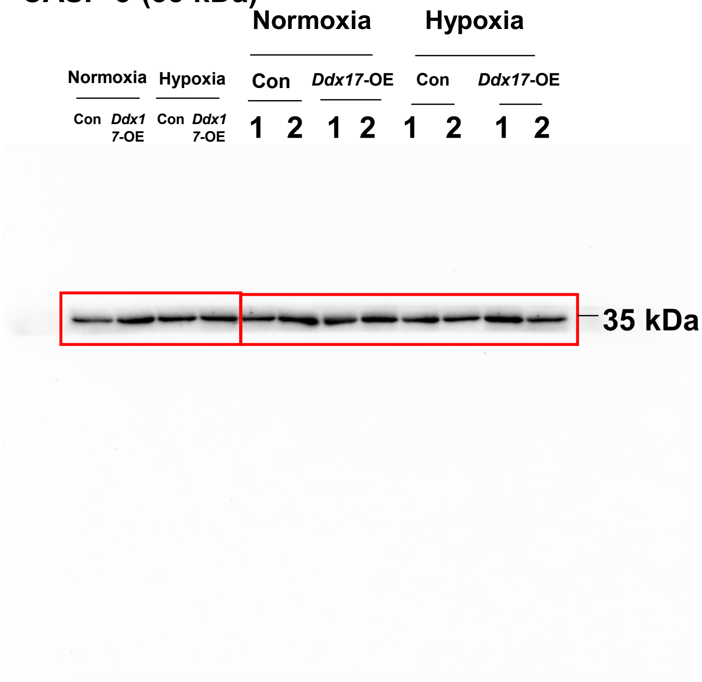


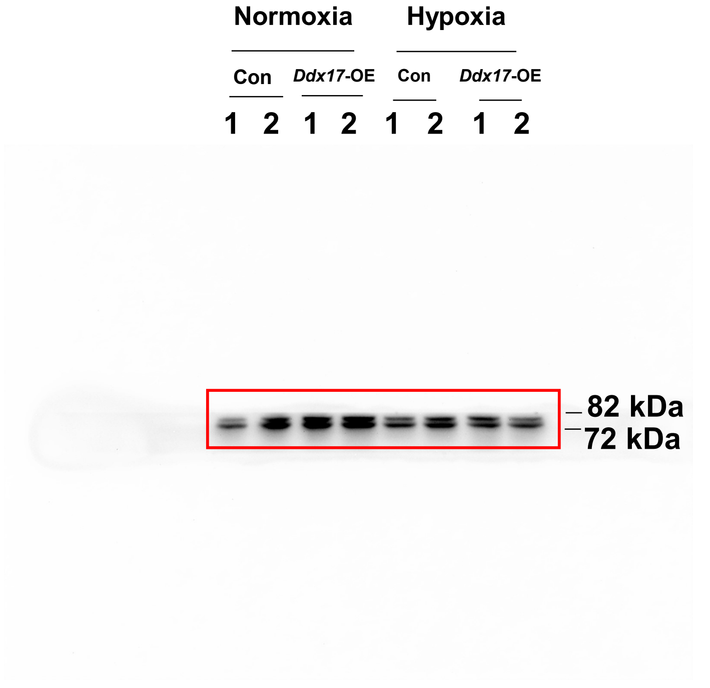


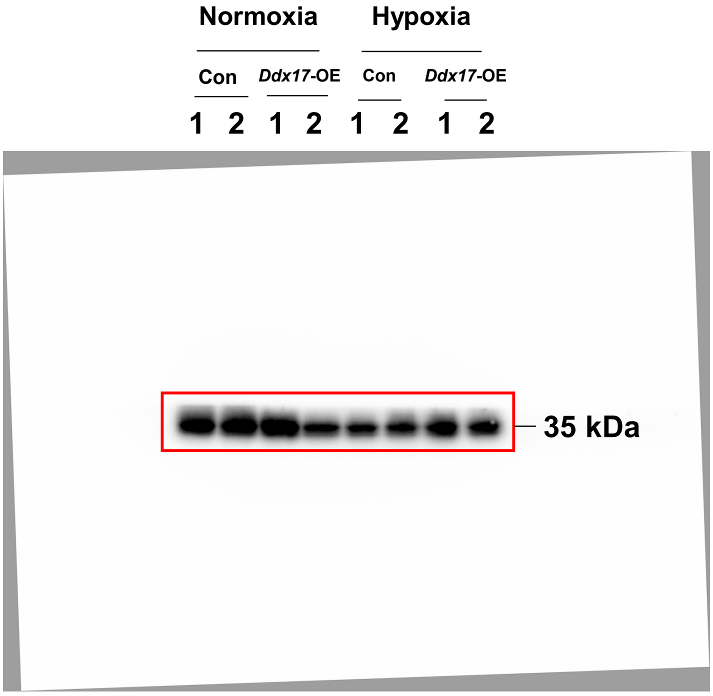


c-CASP-3 (19 and 17 kDa)

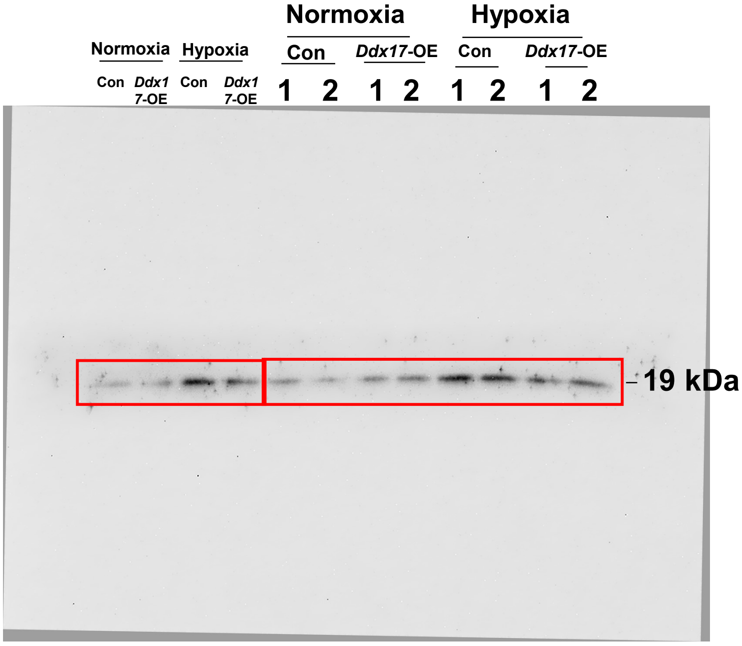


BCL2 (26 kDa)

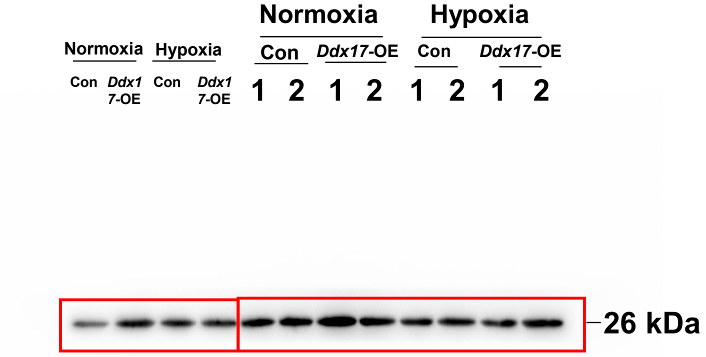

## Supplementary Figure 6g

### c-CASP-3 (19 and 17 kDa)

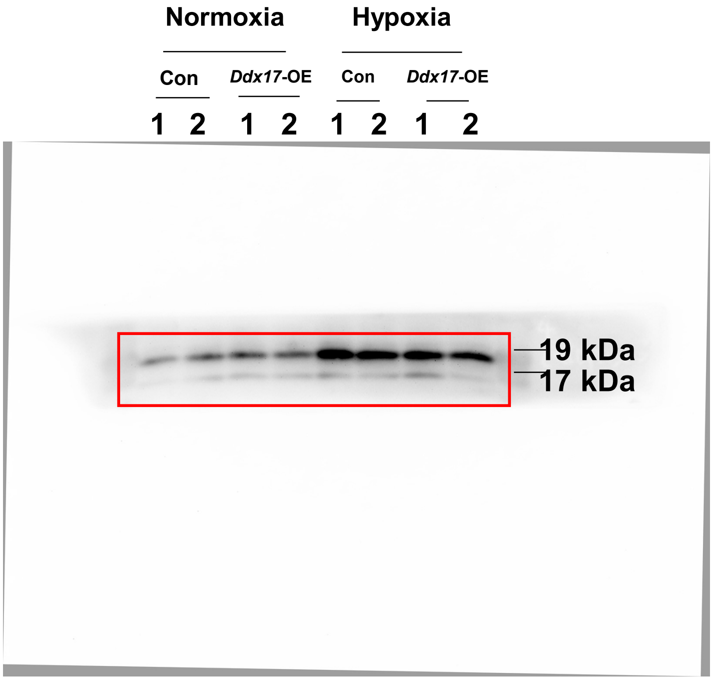


### BCL2 (26 kDa)

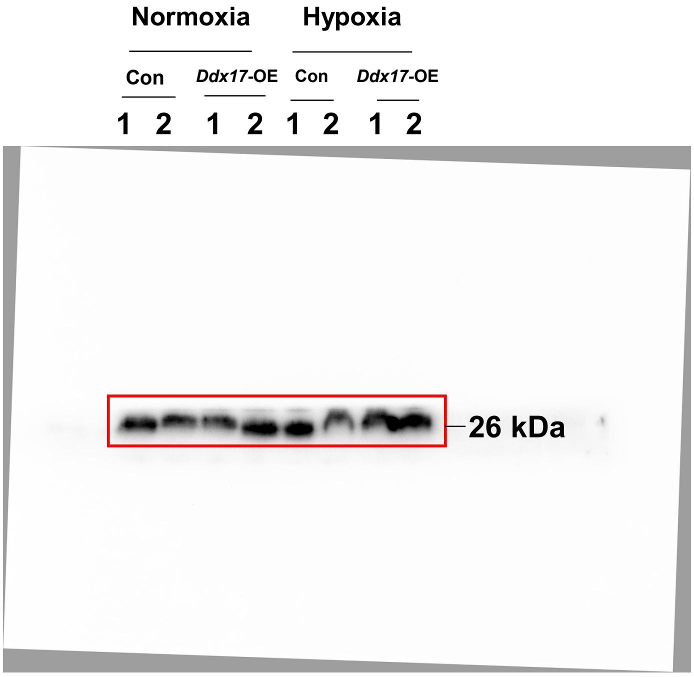


### BAX (21 kDa)

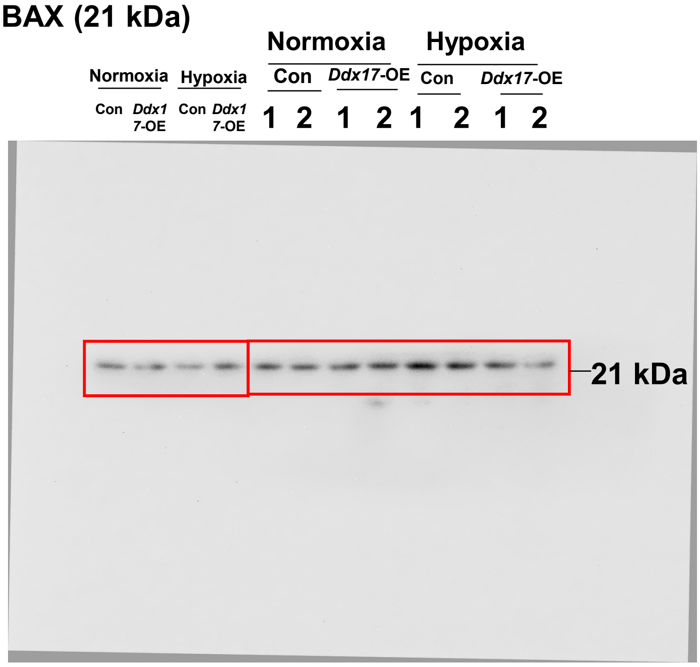


### GAPDH (37 kDa)

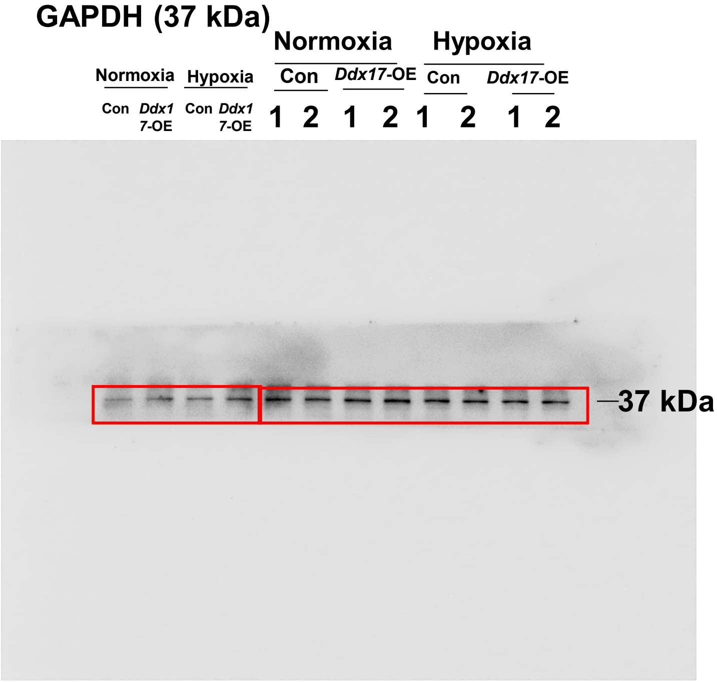


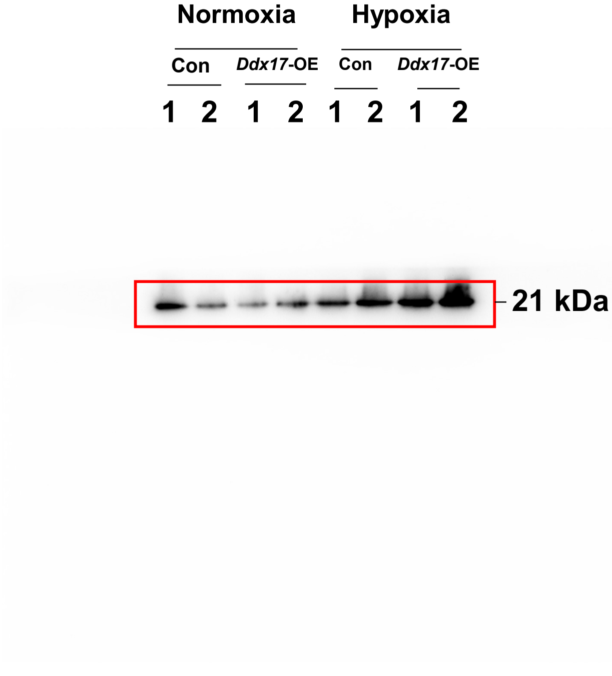


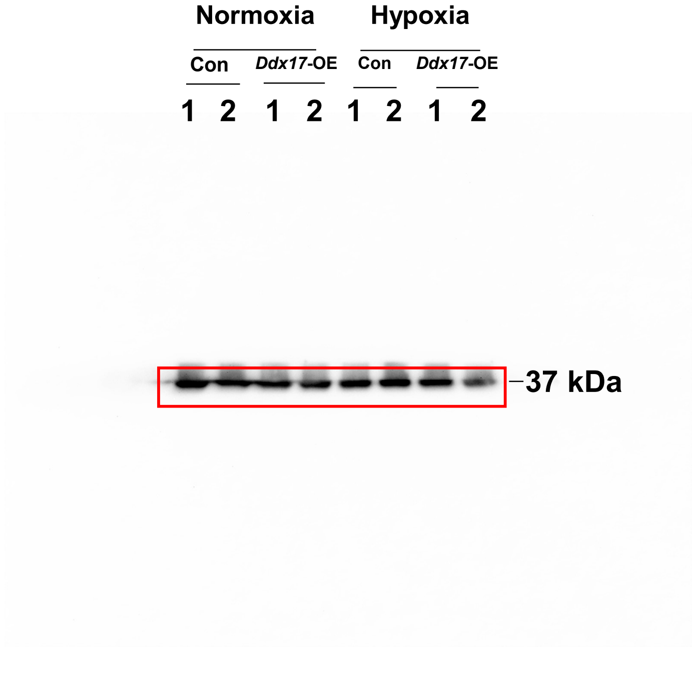

## Supplementary Figure 7g

### DRP1 (80 kDa)

Normoxia | Hypoxia
Con | *Ddx17*-OE | Con | *Ddx17*-OE
1 2 1 2 1 2 1 2

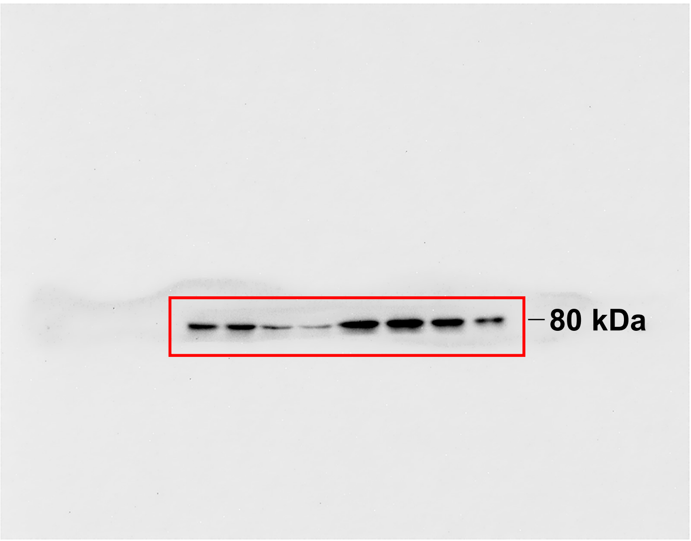


Normoxia | Hypoxia
Con | *Ddx17*-OE | Con | *Ddx17*-OE

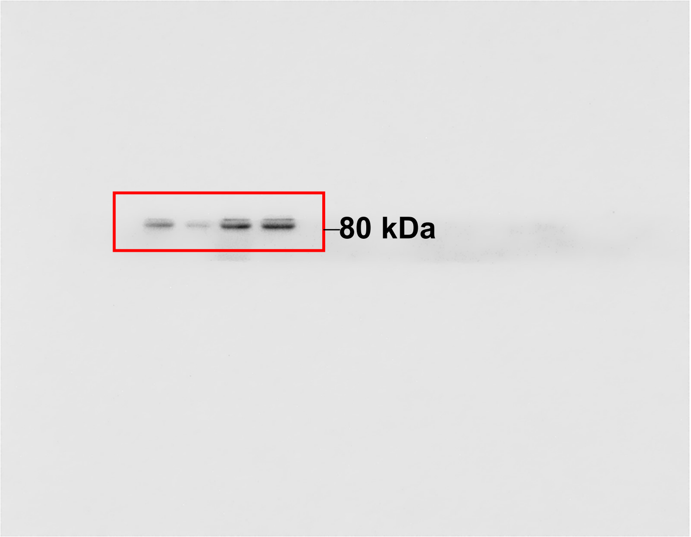


Normoxia | Hypoxia
Con | *Ddx17*-OE | Con | *Ddx17*-OE

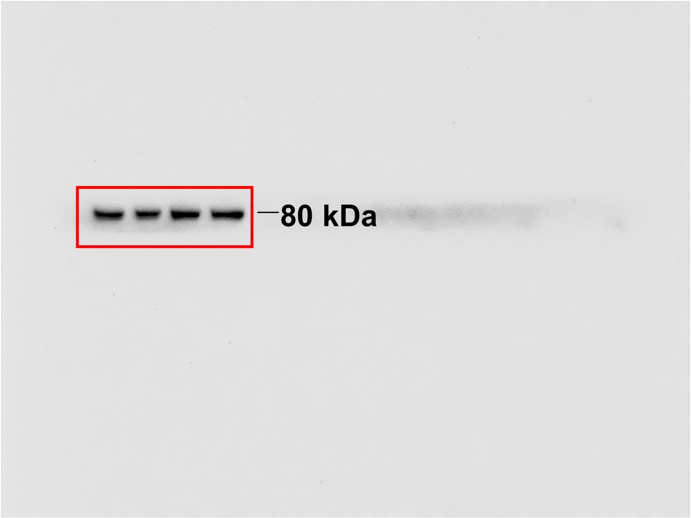


### MFN1 (86 kDa)

Normoxia | Hypoxia
Con | *Ddx17*-OE | Con | *Ddx17*-OE
1 2 1 2 1 2 1 2

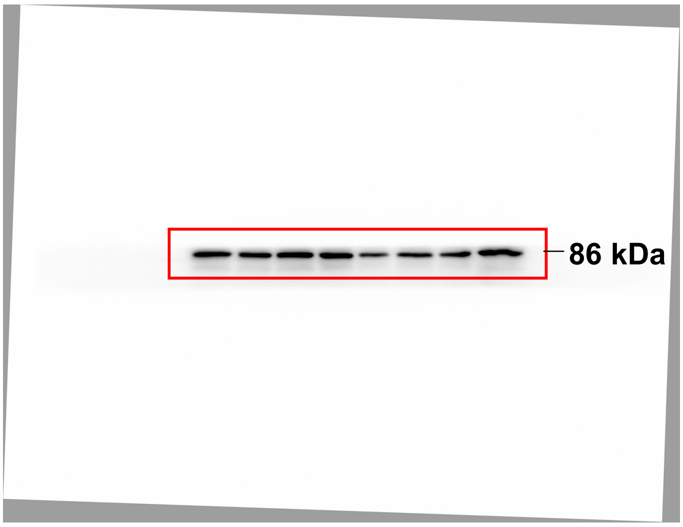


Normoxia | Hypoxia
Con | *Ddx17*-OE | Con | *Ddx17*-OE

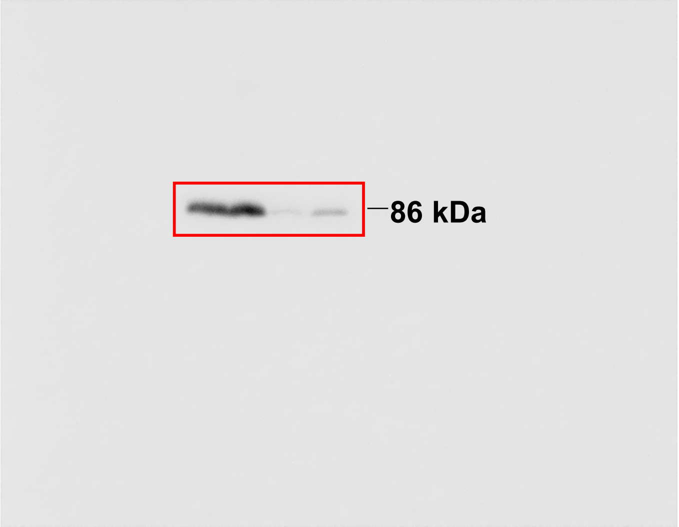


Normoxia | Hypoxia
Con | *Ddx17*-OE | Con | *Ddx17*-OE

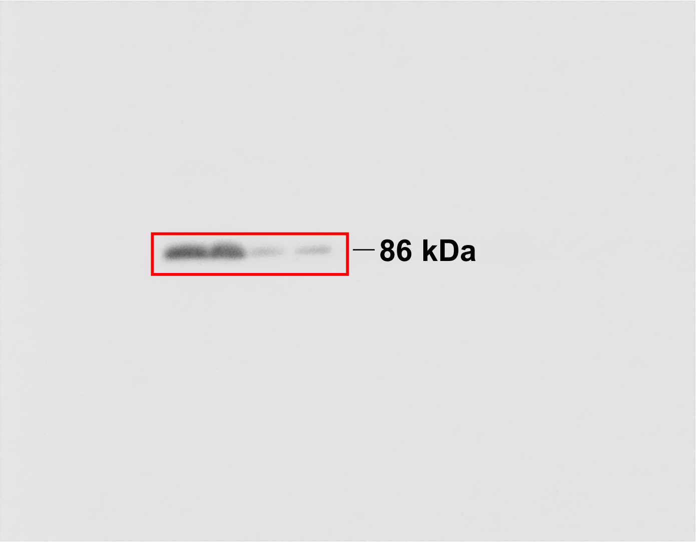

## Supplementary Figure 7g

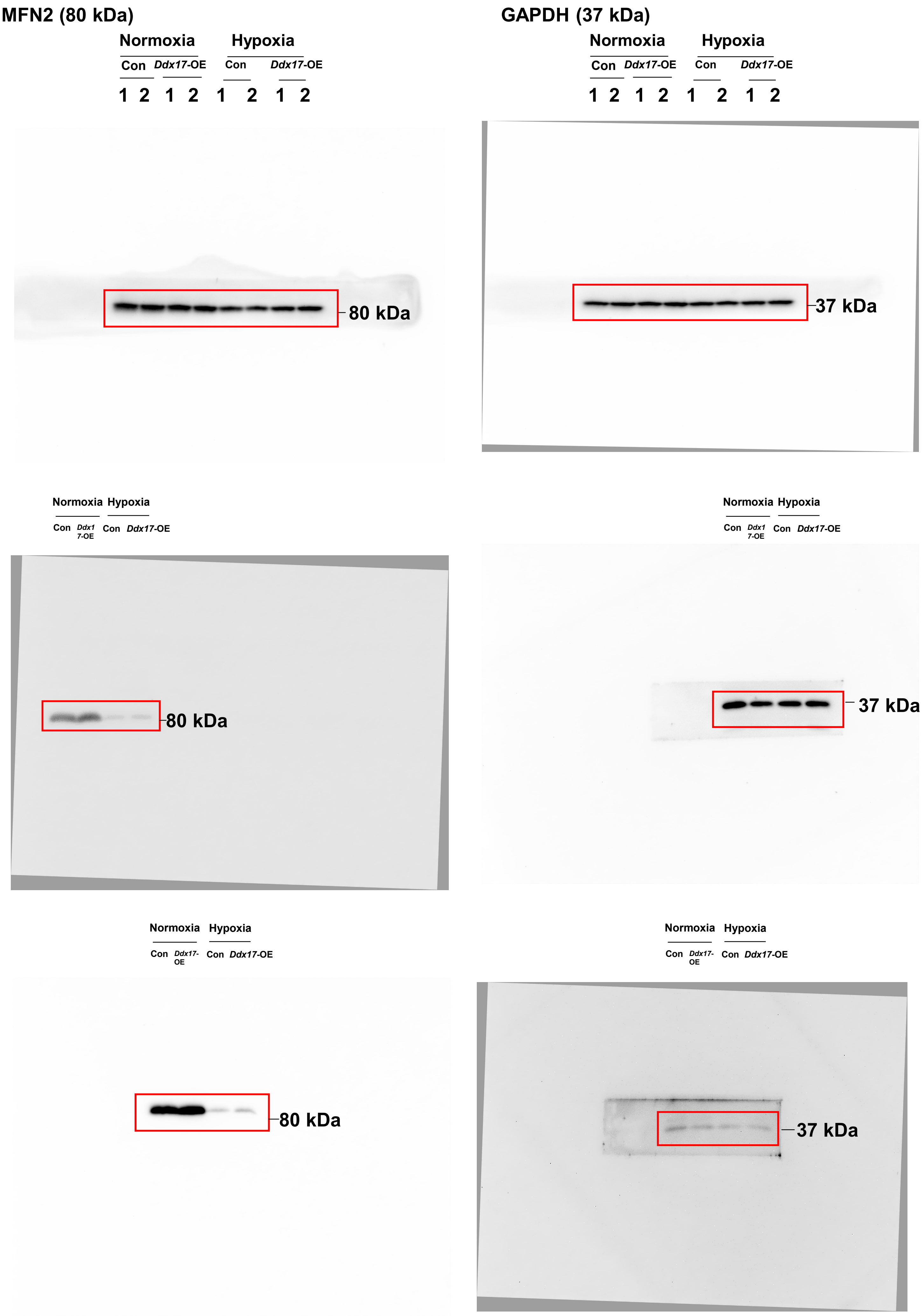

## Supplementary Figure 7g

### GAPDH (37 kDa)

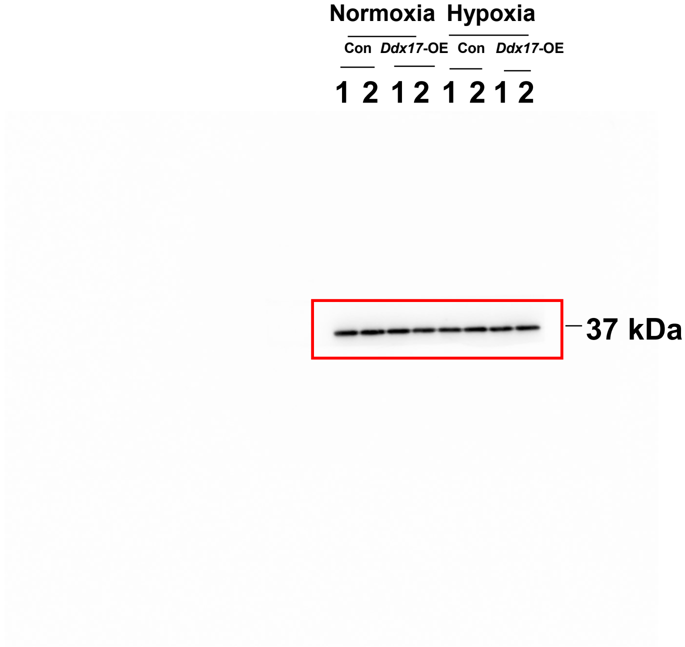


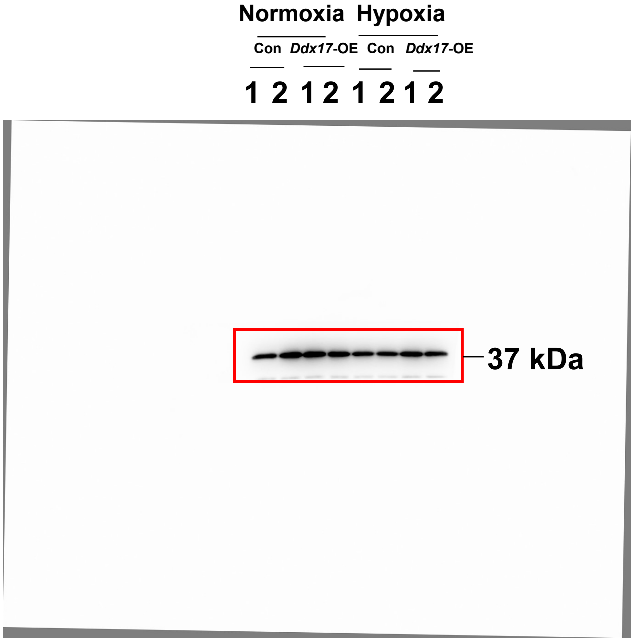


## Supplementary Figure 7k

### DRP1 (80 kDa)

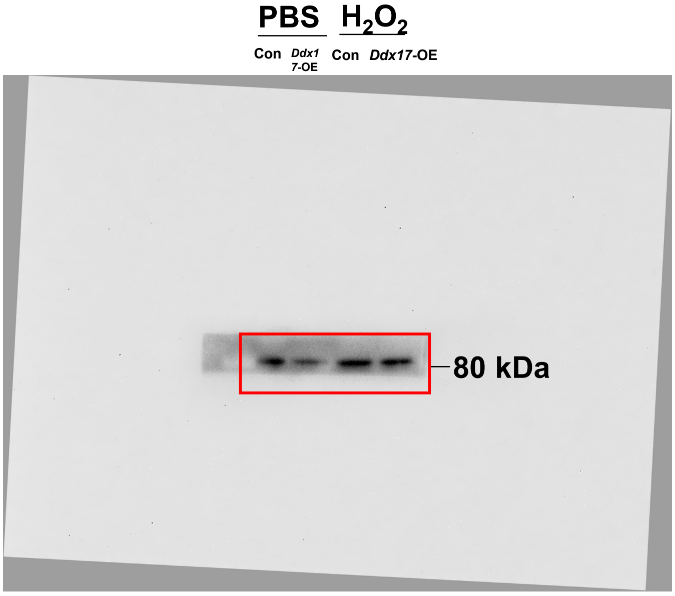


### MFN1 (86 kDa)

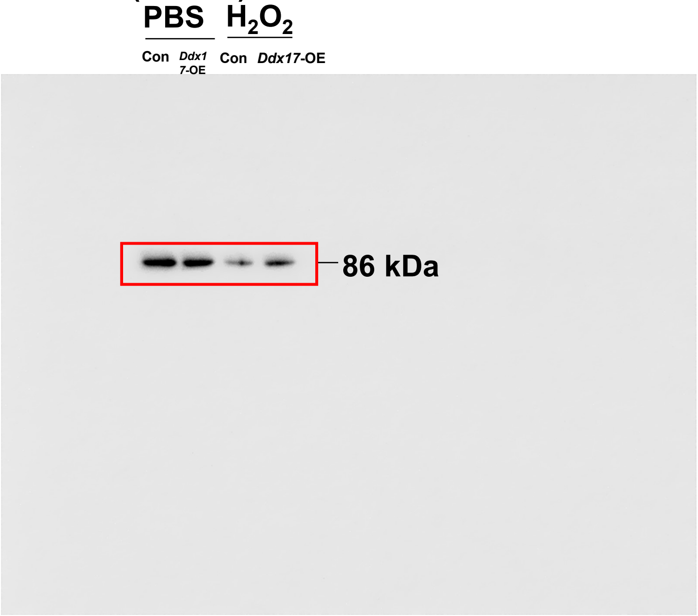


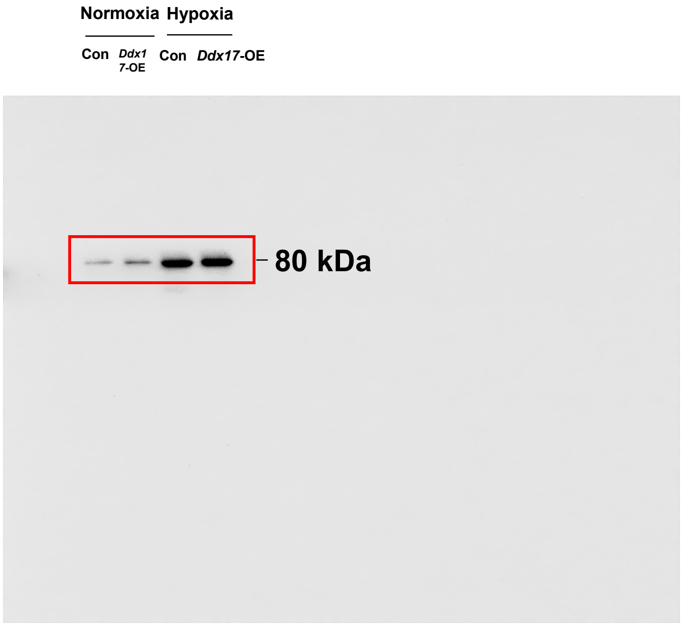


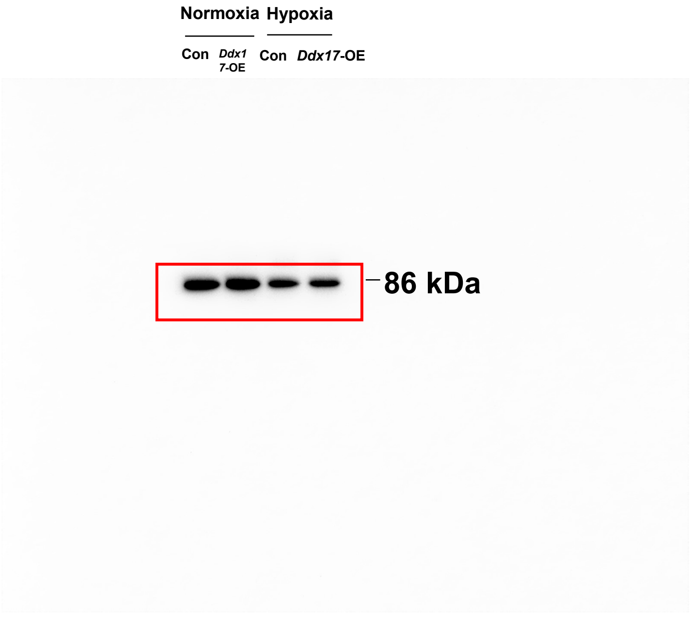

## Supplementary Figure 7k

### DRP1 (80 kDa)

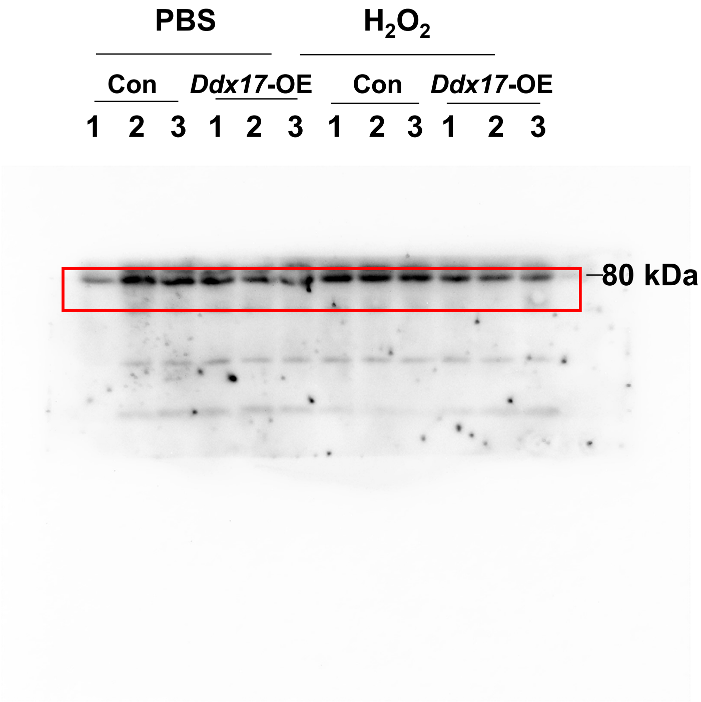


### MFN1 (86 kDa)

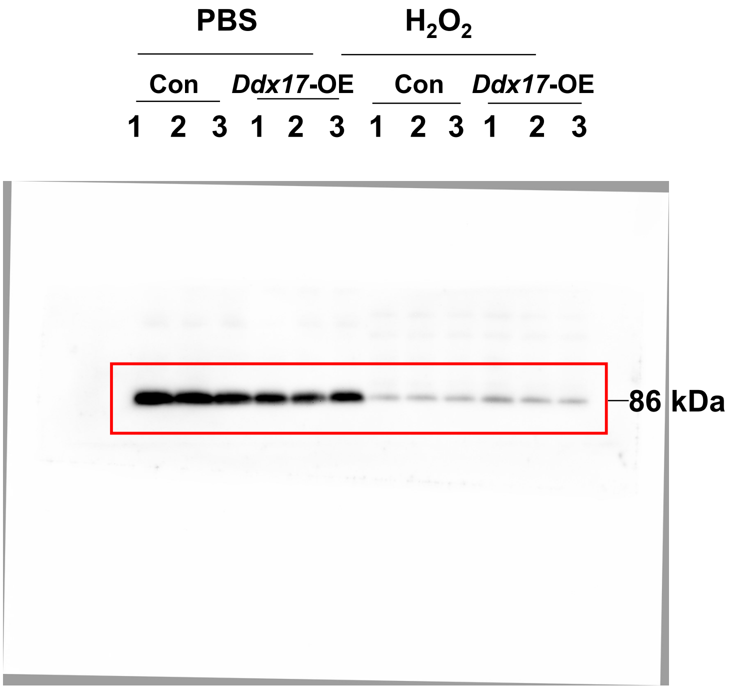


### MFN2 (80 kDa)

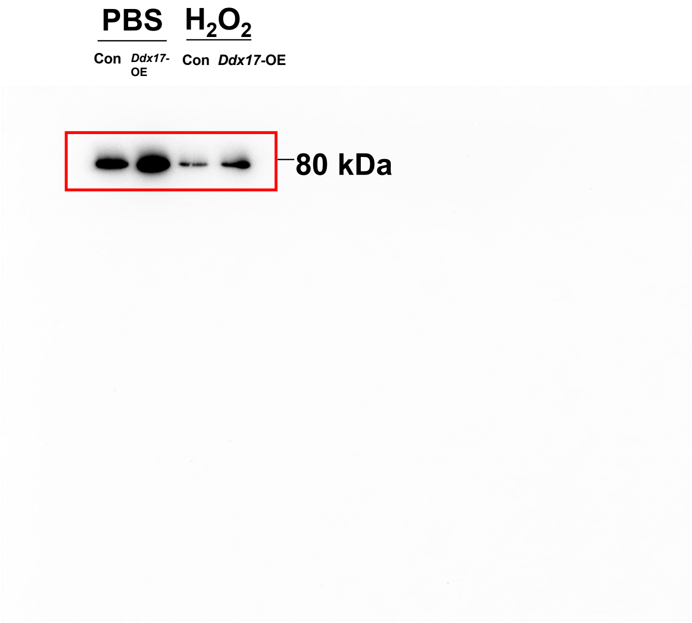


### GAPDH (37 kDa)

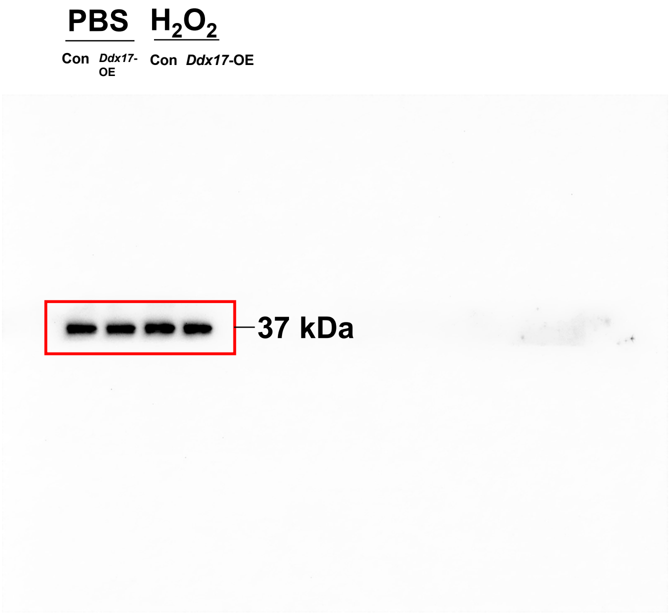


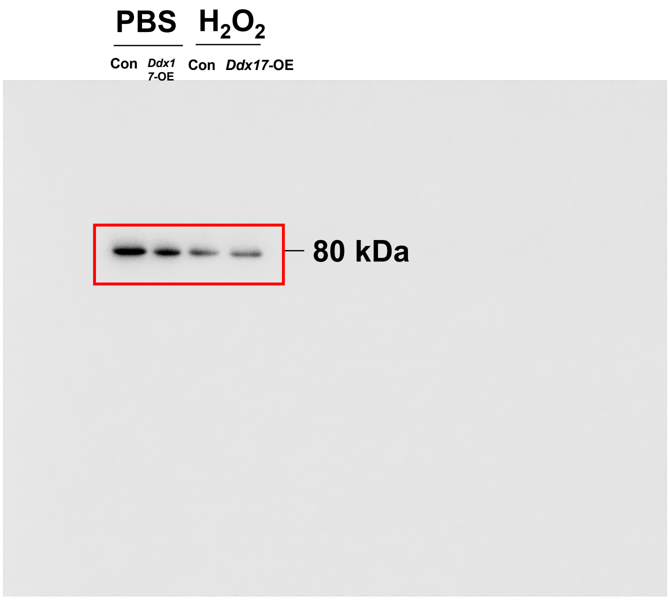


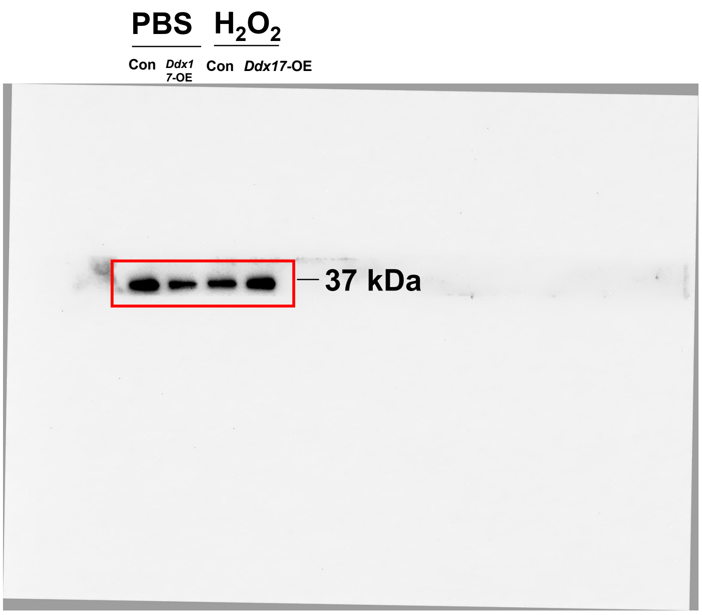

## Supplementary Figure 7k

### MFN2 (80 kDa)

PBS | $H_2O_2$

Con | *Ddx17*-OE | Con | *Ddx17*-OE

1 2 3 1 2 3 1 2 3 1 2 3

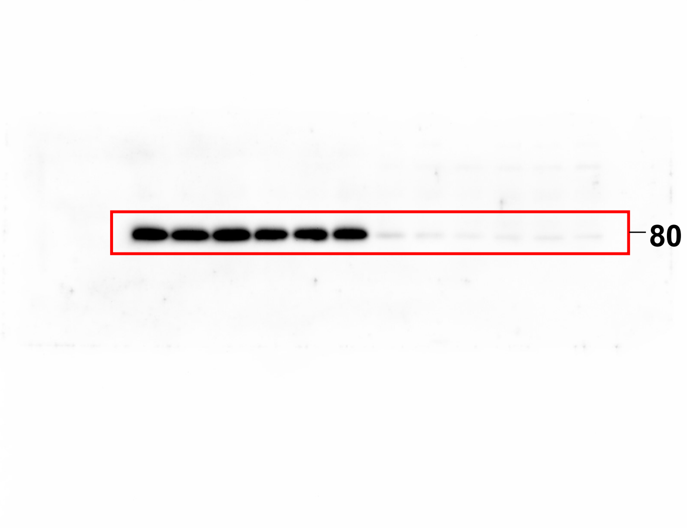


### GAPDH (37 kDa)

PBS | $H_2O_2$

Con | *Ddx17*-OE | Con | *Ddx17*-OE

1 2 3 1 2 3 1 2 3 1 2 3

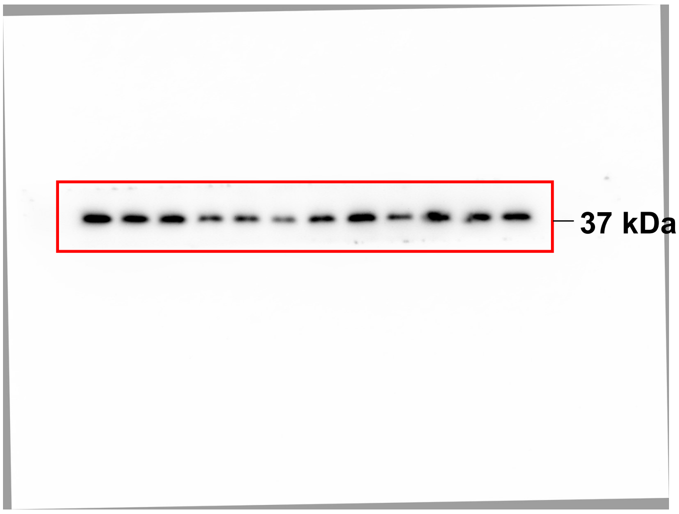


### GAPDH (37 kDa)

PBS $H_2O_2$

Con OE Con OE

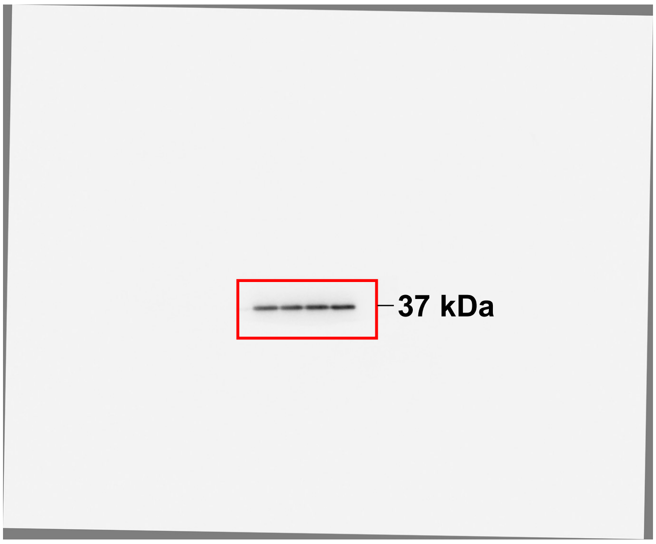


PBS $H_2O_2$

Con OE Con OE

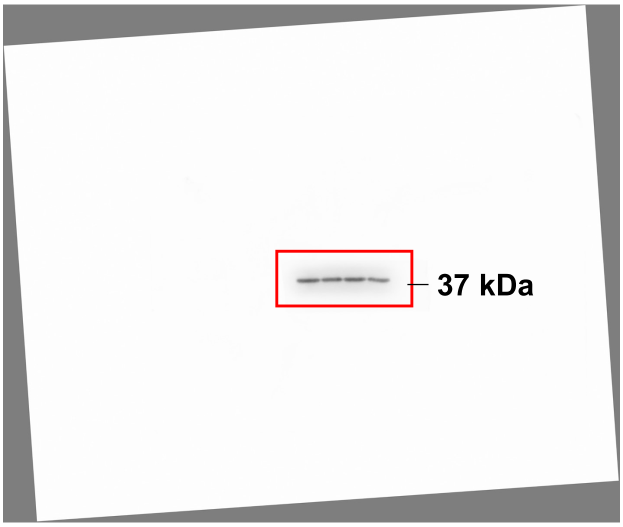


## Supplementary Figure 7o

### OPA1 (80-100 kDa)

Con | *Ddx17*-cKO

1 2 3 4 5 6 1 2 3 4 5 6

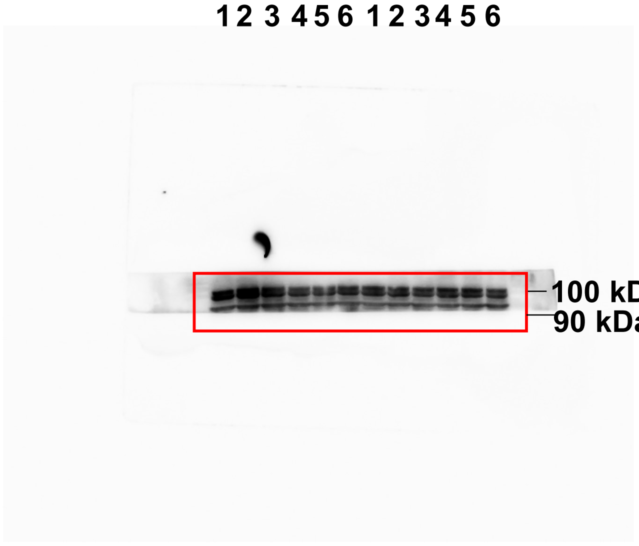


### MFF (35 and 38 kDa)

Con | *Ddx17*-cKO

1 2 3 4 5 6 1 2 3 4 5 6

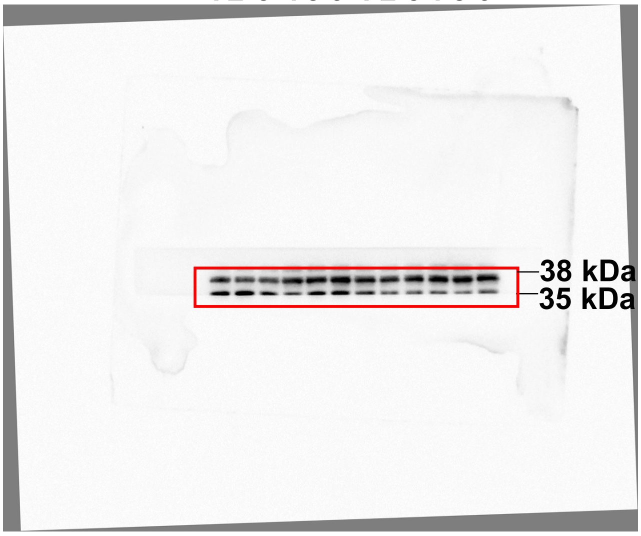

**Supplementary Figure 7o**

**FIS1 (17 kDa)**

Con *Ddx17*-cKO
1 2 3 4 5 6 1 2 3 4 5 6

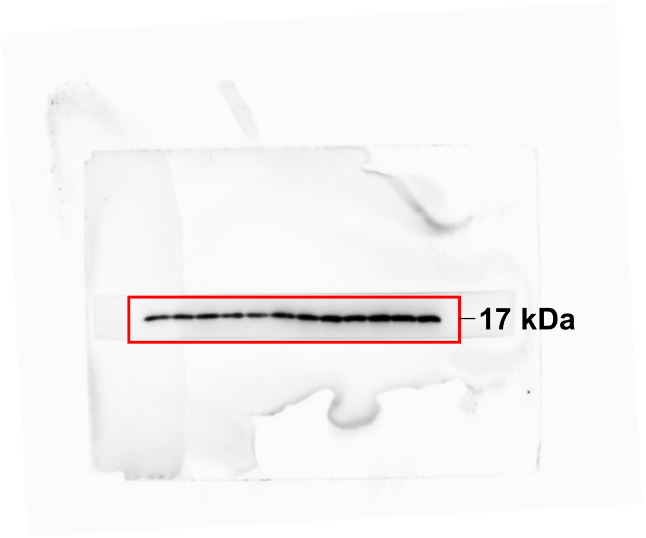


**Tubulin (55 kDa)**

Con *Ddx17*-cKO
1 2 3 4 5 6 1 2 3 4 5 6

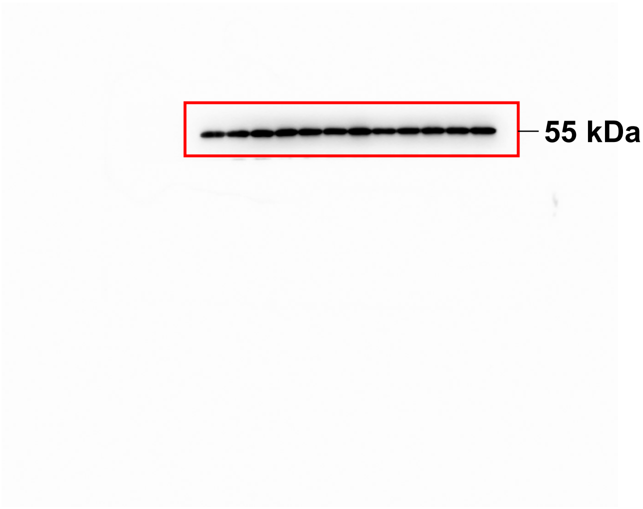


**Supplementary Figure 8c**

**DRP1 (80 kDa)**

Con *Ddx17*-cKO
1 2 3 4 5 6 1 2 3 4 5 6

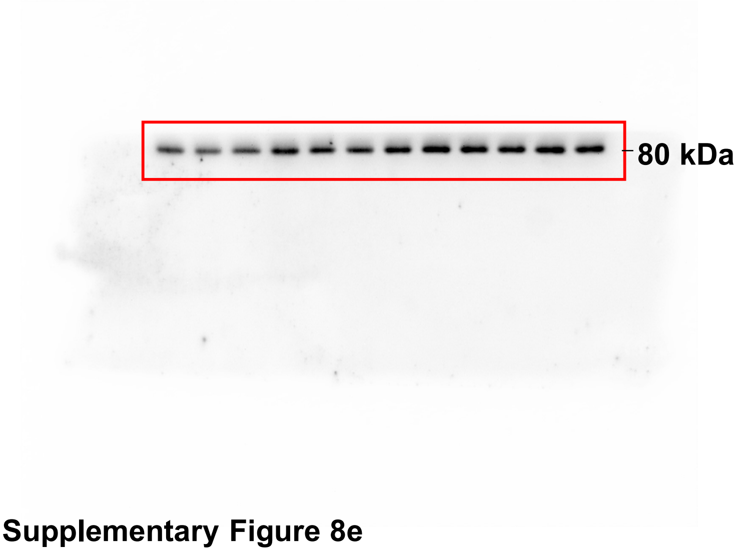


**GAPDH (37 kDa)**

Con *Ddx17*-cKO
1 2 3 4 5 6 1 2 3 4 5 6

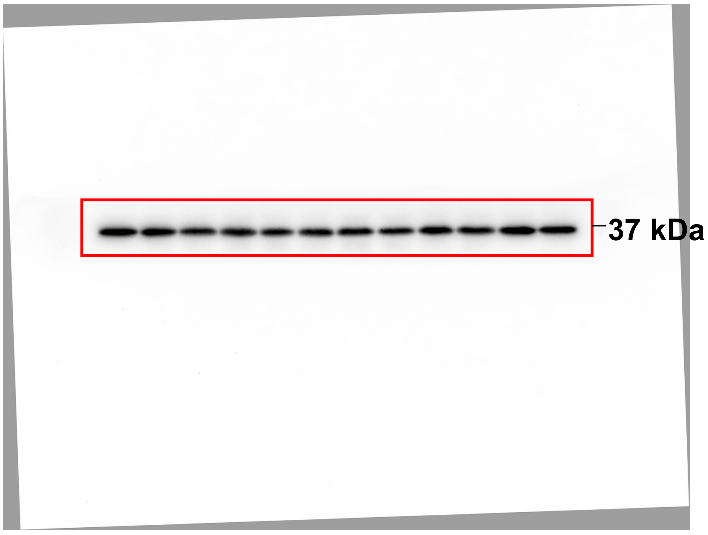


**Supplementary Figure 8e**

**DRP1 (80 kDa)**

Con *Ddx17*-Tg
1 2 3 4 5 6 1 2 3 4 5 6

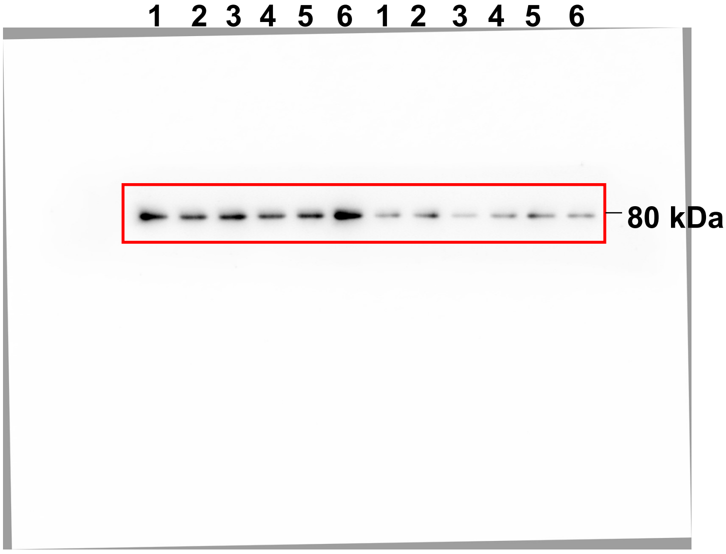


**GAPDH (37 kDa)**

Con *Ddx17*-Tg
1 2 3 4 5 6 1 2 3 4 5 6

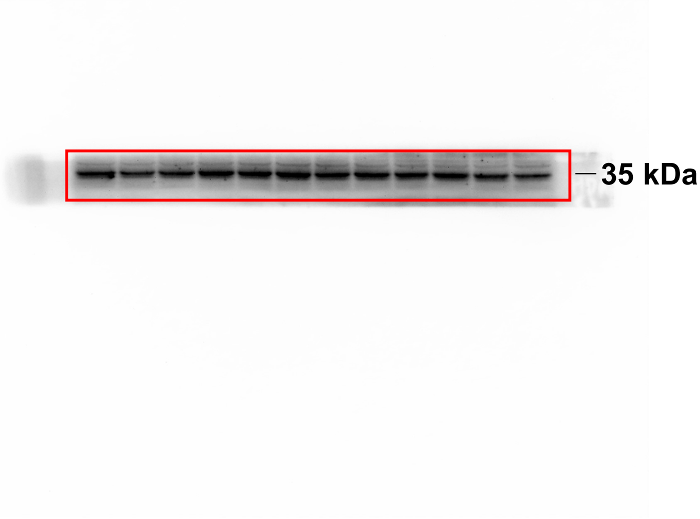

Supplement: Supplementary file 2 — Original image of Western blot [file 41392_2024_1831_MOESM2_ESM.pdf]
